# Whole-cell modeling of *E. coli* confirms that *in vitro* tRNA aminoacylation measurements are insufficient to support cell growth and predicts a positive feedback mechanism regulating arginine biosynthesis

**DOI:** 10.1093/nar/gkad435

**Published:** 2023-05-24

**Authors:** Heejo Choi, Markus W Covert

**Affiliations:** Department of Bioengineering, Stanford University, 443 Via Ortega, Stanford, CA 94305, USA; Department of Bioengineering, Stanford University, 443 Via Ortega, Stanford, CA 94305, USA

## Abstract

In *Escherichia coli*, inconsistencies between *in vitro* tRNA aminoacylation measurements and *in vivo* protein synthesis demands were postulated almost 40 years ago, but have proven difficult to confirm. Whole-cell modeling can test whether a cell behaves in a physiologically correct manner when parameterized with *in vitro* measurements by providing a holistic representation of cellular processes *in vivo*. Here, a mechanistic model of tRNA aminoacylation, codon-based polypeptide elongation, and N-terminal methionine cleavage was incorporated into a developing whole-cell model of *E. coli*. Subsequent analysis confirmed the insufficiency of aminoacyl-tRNA synthetase kinetic measurements for cellular proteome maintenance, and estimated aminoacyl-tRNA synthetase *k*_cat_s that were on average 7.6-fold higher. Simulating cell growth with perturbed k_cat_s demonstrated the global impact of these *in vitro* measurements on cellular phenotypes. For example, an insufficient *k*_cat_ for HisRS caused protein synthesis to be less robust to the natural variability in aminoacyl-tRNA synthetase expression in single cells. More surprisingly, insufficient ArgRS activity led to catastrophic impacts on arginine biosynthesis due to underexpressed N-acetylglutamate synthase, where translation depends on repeated CGG codons. Overall, the expanded *E. coli* model deepens understanding of how translation operates in an *in vivo* context.

## Graphical Abstract

**Graphical Abstract:**
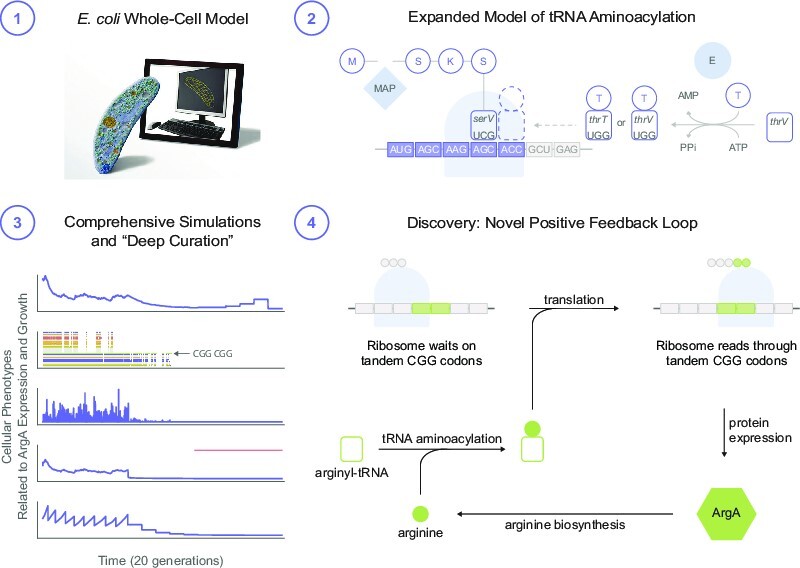

## INTRODUCTION

Since the introduction of the first tissue culture—established in principle by Wilhelm Roux in 1885 and later demonstrated by Ross Harrison in 1907 (1)—*in vitro* studies have enabled detailed investigations performed in controlled environments, leading to countless important discoveries and insights. That said, extrapolating such findings to living cellular contexts is challenged by the degree to which the *in vitro* environment reflects its *in vivo* counterpart. As such, consideration of the *in vivo* context is essential for determining the impact *in vitro* findings may have on the coordinated system of biological processes occurring inside living cells (2). Essential as it may be, the *in vivo* context is often not experimentally accessible. What is therefore needed are methods that enable us to accurately estimate *in vivo* properties—or evaluate *in vitro* measurements—in the background of a holistic cellular context.

In *Escherichia coli*, one important example of *in vitro* measurements being inconsistent with *in vivo* demands concerns tRNA aminoacylation and protein synthesis, as first identified by Jakubowski and Goldman almost forty years ago (3). Seeking to determine the turnover of aminoacyl-tRNAs *in vivo*, Jakubowski and Goldman pulse-labeled *E. coli* cultures with radioactive amino acids. By dividing their measurements of amino acid incorporation rates into protein (molecules per cell per second) by the amounts of aminoacyl-tRNA synthetase (molecules per cell), they were able to estimate the lower limits of aminoacyl-tRNA synthetase activities (s^-1^). Comparing these minimal *in vivo* activities with *in vitro* measurements of purified preparations of aminoacyl-tRNA synthetases led to a surprising inconsistency: with one exception (GluRS), *in vitro* reports were 3- to 240-fold lower than their most conservative estimates of *in vivo* activities. This study raised several critical questions that remain unanswered, such as: Why do these *in vitro* measurements underestimate cellular demands so dramatically? If the measured activities are truly too low to support the cell’s needs, how much higher must they be? And finally, could our reliance on the *in vitro* measurements cause us also to miss or misinterpret important cellular phenotypes?

Addressing these questions requires the use of computational approaches that can incorporate *in vitro* measurements into a simulation of the *in vivo* context. In particular, whole-cell modeling is an approach that takes into account all the genes and known functions of an organism to predict phenotypes—consolidating millions of data points into a dynamic representation of the intracellular system during the cell cycle. A major advantage of whole-cell modeling is that multiple biological processes—such as chromosome replication, transcription, transcriptional regulation, translation, metabolism, RNA and protein degradation, complexation, and cell division—are simulated simultaneously as the *in vivo* environment evolves over time. Accordingly, whole-cell models provide the *in vivo* context that tests whether the cell can behave in a physiologically correct manner when parameterized with measurements and reveals the propagating impact of these measured parameters throughout the intracellular system, thereby offering a rich environment for discovery.

Aminoacylation of tRNAs has posed an intriguing modeling challenge in previous large-scale models developed by our group, including *Mycoplasma genitalium* in 2012 (4) and *E. coli* in 2020 (5). Pools of tRNAs are known to turnover rapidly, with pulse-labeling measurements reporting that individual tRNA molecules undergo 1.8 to 8.1 aminoacylation cycles per second depending on the amino acid family (3). Within the whole-cell modeling framework, which assumes that time steps are short enough to consider biological submodels independently, these fast turnovers meant that the separation of tRNA aminoacylation from translation would cause tRNA pools to deplete during the simulated time step, which is typically set to 1–2 s. Several solutions were considered to overcome this obstacle: (i) shorter time steps, which would increase the runtime, (ii) overexpression of tRNAs, which helped to enable the *M. genitalium* model (4), and (iii) assuming a sufficient supply of aminoacyl-tRNAs and approximating their role by direct polymerization of amino acids, as implemented in the first version of the *E. coli* model (5). However, none of these approaches are able to address the central questions posed by Jakubowski and Goldman because they do not include the mechanistic representation or kinetic information that would be required.

Other groups have focused specifically on tRNAs in their models of translation. For example, Elf and colleagues modeled how aminoacylation levels of different tRNA isoacceptors respond when their cognate amino acids become growth-limiting, as informed by tRNA concentrations, codon usage frequencies, and codon specificities of different isoacceptors (6). Their work presented the theory of selective aminoacylation during amino acid limitation—meaning that different isoacceptors of the same amino acid will reach different steady state aminoacylation levels in response to limitation of their cognate amino acid—and predicted codon sensitivities to amino acid starvation. Levin and Tuller modeled the major components of translation—such as ribosomes, mRNAs and tRNAs—and competition for ribosomes and tRNAs at the codon resolution using a novel Multiple Pool State Machine Translation Model (MP-SMTM) approach (as opposed to a kinetics-based approach) to drive simulation dynamics (7). Their work showed that the MP-SMTM approach could predict the outcome of heterologous gene expression. Although other aspects of the cell related to translation were represented by Levin and Tuller, such as ribosome activity and mRNA pools reflecting the transcriptome, a complete representation of cellular behavior was not a focus of either of these studies.

Taking inspiration both from these studies and our previous work, we expanded our large-scale model of *E. coli* to describe tRNA aminoacylation rates kinetically. This enabled us to test whether the measured and Jakubowski and Goldman-predicted rate constants related to aminoacylation are sufficient for the cell’s normal function, and if not, to estimate what the required parameter values would need to be. We found that in fact, the measured aminoacyl-tRNA synthetase activities are insufficient to maintain the demands of the cellular proteome and that the required values are on average 7.6-fold higher. We also found that the higher, predicted *in vivo* activities enabled the cell to overcome the ribosome elongation rate’s sensitivity to the natural variability in aminoacyl-tRNA synthetase expression in single cells. Finally, we show that the *in vitro* measurements can lead to catastrophic impacts on cellular phenotype via a predicted regulatory feedback link between aminoacyl-tRNAs and ribosomal pausing at tandem cognate codons—in this case, arginine. In total, our findings suggest that computational modeling can represent a bridge between *in vitro* measurements and *in vivo* understandings.

## MATERIALS AND METHODS

Materials and methods can be categorized into three primary areas: model construction, parameter estimation and details of the specific simulations and follow-on analysis. These are detailed in the Supporting Materials, but are also described briefly below.

### Hybrid deterministic-stochastic model

To be compatible with both the deterministic and stochastic qualities of the tRNA aminoacylation cycle and the whole-cell modeling framework (described in Supporting Materials, Section 1), a three-step strategy was developed: (i) calculate the kinetic limitations of aminoacyl-tRNA synthetases by simulating the tRNA aminoacylation cycle as a deterministic ODE model described by Michaelis-Menten enzyme kinetics, (ii) process ribosomes along their mRNAs according to these kinetic limitations and the sequential order in which ribosomes encounter codons on mRNAs and (iii) stochastically reconcile any disagreements between the kinetic- and sequence-determined constraints. Derivation of the ODEs used, molecules represented, and codon-to-anticodon interactions modeled can be found in Supporting Materials, Section 1.

### Optimization of aminoacyl-tRNA synthetase kinetic parameters

Parameter estimation of the aminoacyl-tRNA synthetase kinetic parameters was described as objective minimization problems, where each aminoacyl-tRNA synthetase was described as an independent optimization problem that aims to minimize the differences between the rates of tRNA aminoacylation and aminoacyl-tRNA utilization. The rates of tRNA aminoacylation were described by Michaelis–Menten enzyme kinetics as in the hybrid deterministic-stochastic model. The rates of aminoacyl-tRNA utilization, or relatedly the codon reading rates, were derived from the principle that the protein content of the cell must grow exponentially and double at the measured doubling time. Design of the objective function, derivation of the codon reading rates, values of constant parameters, and generation of candidate solutions can be found in Supporting Materials, Section 2.

### Simulation and analysis

**Figure 1.**
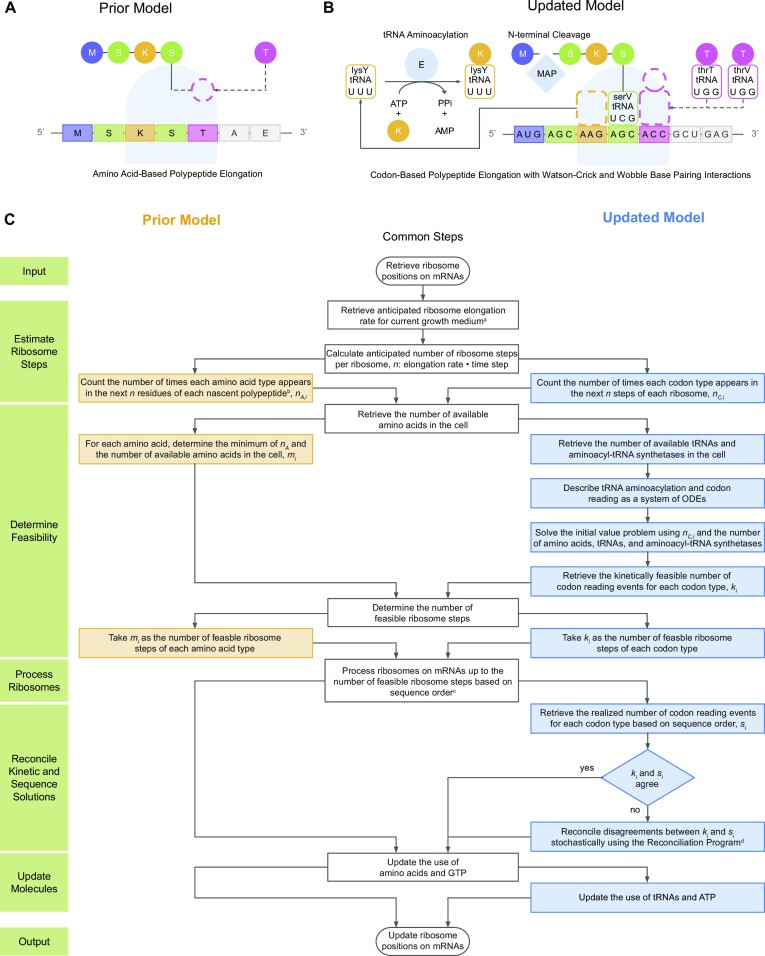
A mechanistic model of tRNA aminoacylation, codon-based polypeptide elongation, and N-terminal methionine cleavage is incorporated into the developing whole-cell model of *E. coli*. (**A**) Prior model of *E. coli* approximated the role of aminoacyl-tRNAs by direct polymerization of amino acids (circles) by ribosomes (light blue) according to the primary sequence of polypeptides (rectangles). With each elongation step, an amino acid (for example, threonine in pink) is directly incorporated (dashed circle) into the nascent polypeptide. Amino acids are labeled with their single-letter abbreviations. (**B**) Updated model (presented in the current study) expanded the translation model by representing the mechanisms of tRNA aminoacylation, codon-based polypeptide elongation, and N-terminal cleavage of initial methionines (Supporting Materials, Section 1). With each elongation step, an aminoacyl-tRNA (for example, a threonyl-*thrT* or threonyl-*thrV* tRNA in pink) interacts with the codon in the open A site (ACC) by Watson-Crick (such as the serine-cognate *serV* tRNA with anticodon 3’-UCG-5’ decoding codon 5’-AGC-3’) or Wobble base pairing (such as the lysine-cognate *lysY* tRNA with anticodon 3’-UUU-5’ decoding codon 5’-AAG-3) to facilitate incorporation (dashed rectangle connected to a dashed circle) of the next residue (threonine). After interaction with the ribosome (light blue), tRNAs (curved-edge rectangles) are available for successive rounds of aminoacylation by aminoacyl-tRNA synthetase enzymes (labeled ‘E’). Nascent polypeptides that undergo N-terminal cleavage of the initial methionine by Methionine Aminopeptidase (labeled ‘MAP’) are cleaved before termination. Colors and sequences are coordinated between panels A and B to aid comparison. (**C**) Comparison of implementations of translation in the prior model (with unlimited tRNA aminoacylation) and updated model (presented in the current study) (Supporting Materials, Section 3). Notes: a: Interpolated from Bremer and Dennis, 2008 (19). b: Uses the primary sequence of mature proteins. c: Ribosomes process sequentially along the amino acid (prior model) or codon (updated model) sequence, such that the most limiting amino acid or codon determines the number of ribosome steps. In the prior model, no further steps are required. However, in the updated model, due to the cyclic relationship between tRNA aminoacylation and codon reading, any excess estimation of codon reading events *k*_*i*_ are incorporated back into the calculation of tRNA aminoacylation events (from the mass action kinetics model) through the Reconciliation Program. d: The Reconciliation Program is detailed in Supplementary Figure S1 and Supporting Materials, Section 3.3.

The model presented in this study was implemented into the polypeptide elongation sub-model of the first version of the *E. coli* model (5). A comparison of the prior and updated models and a summary-level flowchart of the computational steps that take place within each time step is shown in Figure 1. The primary changes to the polypeptide elongation sub-model occur in the estimation of ribosome steps, determination of kinetic and codon sequence order feasibility, reconciliation of kinetic and sequence solutions, and update of molecular abundances. These changes are described in Supporting Materials, Section 3. Additionally, the total set of simulations and analyses performed are detailed in Supporting Materials, Section 4.

## RESULTS

### A mechanistic model of tRNA aminoacylation, codon-based polypeptide elongation, and N-terminal methionine cleavage is incorporated into the developing whole-cell model of *E. coli*

To better account for the tRNA-related translation mechanisms noted above, we expanded the translation model contained within the most recently published version of the *E. coli* Whole-Cell Modeling Project (5,8) in three primary areas: tRNA aminoacylation, codon-based polypeptide elongation, and N-terminal cleavage of initial methionines. A schematic of the prior model (5) is shown in Figure 1A, and the new, expanded model is shown in Figure 1B.

First, we incorporated the aminoacylation of tRNAs by aminoacyl-tRNA synthetases. As mentioned above, the prior model assumed that the supply of aminoacyl-tRNA synthetases and aminoacyl-tRNAs were sufficiently abundant and did not limit the elongation rate of ribosomes. In the current study, aminoacylation was represented according to Michaelis–Menten enzyme kinetics, which enabled the aminoacylation rates to respond to changes in abundances of aminoacyl-tRNA synthetase enzymes and their substrates (Supporting Materials, Section 1.1). This addition introduced the description of aminoacyl-tRNAs to our simulated cells.

Second, to facilitate amino acid transfer by elongating ribosomes, the amino acid-based polypeptide elongation design of the prior model was detailed to the codon level as part of a hybrid deterministic-stochastic model (Supporting Materials, Sections 1.2 and 1.3). Codon-to-anticodon interactions were designed to obey Watson-Crick base pairing rules (such as the serine-cognate *serV* tRNA with anticodon 3’-UCG-5’ decoding codon 5’-AGC-3’ in Figure 1B) and the Wobble Hypothesis (such as the lysine-cognate *lysY* tRNA with anticodon 3’-UUU-5’ decoding codon 5’-AAG-3’, also in Figure 1B)—with the exception of three codons for which experimental measurements indicated more specific interactions. These exceptions are: arginine codons 5’-CGA-3’ and 5’-CGC-3’, which were reported to be decoded by tRNA isoacceptors *argQ*, *argV*, *argY* and *argZ* with anticodon 3’-GCI-5’, where I is inosine—an adenosine derivative (9,10), and isoleucine codon 5’-AUA-3’, which was reported to be decoded by tRNA isoacceptors *ileX* and *ileY* with anticodon 3’-UAL-5’, where L is lysidine—a cytidine derivative (11,12).

Third, we incorporated the N-terminal cleavage of initial methionines by Methionine Aminopeptidase (MAP) (Supporting Materials, Section 1.4). Due to its use of the primary amino acid sequence of polypeptides, the prior model synthesized proteins in their mature form. In contrast, by incorporating codon-based polypeptide elongation in this study, we synthesized immature forms of polypeptides including the initial methionine residue of all nascent polypeptides. In turn, this development facilitated the description of N-terminal cleavage of initial methionines by MAP for its annotated substrates (13).

These expansions were implemented in the polypeptide elongation sub-model of the *E. coli* model (Figure 1C and Supporting Materials, Section 3). When estimating the number of steps ribosomes were anticipated to take during a time step, the prior model’s retrieval of the amino acid sequence of the mature polypeptide (*n*_*A*, *i*_) was replaced with the current model’s retrieval of the codon sequence of the mRNA transcript (*n*_*C*, *i*_). Consequently, the determination of the number of feasible ribosome steps was changed from a comparison of *n*_*A*, *i*_ with the number of available amino acids in the cell to a calculation of the kinetically-feasible number of codon reading events, as determined by solving a system of ODEs describing the tRNA aminoacylation cycle. Afterwards, ribosomes process along mRNAs according to the sequence order of codons while obeying the feasible number of elongation steps that can occur. Any disagreements between the kinetically-determined and sequence-determined number of ribosome steps are reconciled stochastically (Supplementary Figure S1 and Supporting Materials, Section 3.3).

In summary, these improvements introduced the functional representations of 22 synthetase subunits, all 85 tRNAs of the 20 canonical amino acids, the 61 sense codons, and MAP (Table 1).

**Table 1.** Functional representations introduced by the updated model. The functional representations of 22 synthetase subunits (forming 20 aminoacyl-tRNA synthetase species), 85 tRNAs (in their aminoacylated and unaminoacylated forms) for the 20 canonical amino acids, the 61 sense codons, and Methionine Aminopeptidase have been incorporated into the *E. coli* model (5) by this study

Functional representations introduced		
Translation components	Number of representations	Number of genes
Aminoacyl-tRNA synthetase	20 species	22
tRNA	85 species	85
Codons	61 sense codons	N/A
Methionine aminopeptidase	1 species	1

### Deep curation confirms and quantifies the insufficiency of aminoacyl-tRNA synthetase kinetic measurements for maintenance of the cellular proteome

**Figure 2.**
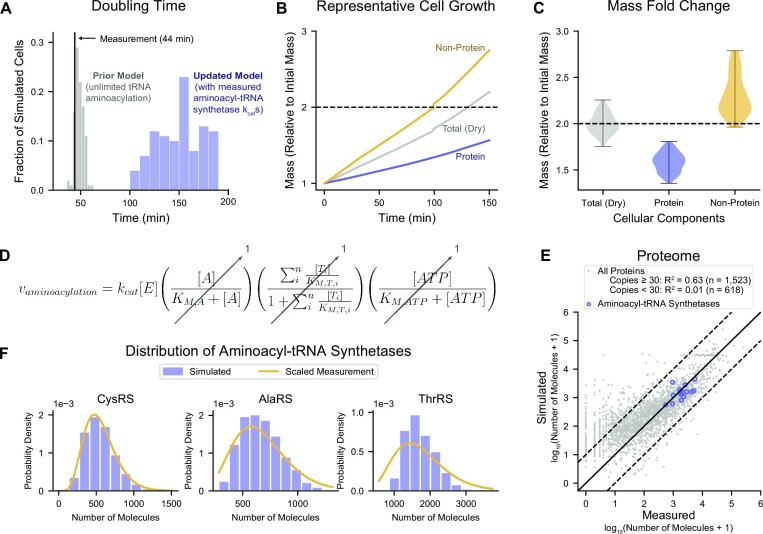
Deep curation confirms and quantifies the insufficiency of aminoacyl-tRNA synthetase kinetic measurements for maintenance of the cellular proteome. (**A**) Distribution of doubling times of cells simulated in the prior model (*n* = 100 cells, gray) and in the updated model when using the measured aminoacyl-tRNA synthetase *k*_cat_s (n=100 cells, blue)—compared to the experimentally measured doubling time (black). (**B**) Mass accumulation of total dry cell (gray), protein (blue), and non-protein (yellow) in a representative cell simulated in the updated model with the measured aminoacyl-tRNA synthetase *k*_cat_s. The representative cell (Variant 2, Seed 11, Generation 0) was chosen for exhibiting a doubling time that is closest to the population average. Black dashed line represents the expectation of mass doubling during exponential growth. (**C**) Distribution of mass fold changes of total dry cell (gray), protein (blue), and non-protein (yellow) in cells simulated in the updated model with the measured aminoacyl-tRNA synthetase *k*_cat_s (*n* = 100 cells). Error bars indicate the range of observed values. Black dashed line represents the expectation of mass doubling during exponential growth. (**D**) Rate of tRNA aminoacylation described by Michaelis–Menten enzyme kinetics representing random-ordered substrate-binding and competition between tRNA isoacceptors for the aminoacyl-tRNA synthetase. Maximal rates were assumed by setting the fractional components to 1. Notations are: [*E*] = aminoacyl-tRNA synthetase concentration, [*A*] = amino acid concentration, [*T*_*i*_] = *i*th tRNA isoacceptor (unaminoacylated form), [*ATP*] = ATP concentration, *k*_cat_ = rate of catalysis, *K*_M,A_ = Michaelis–Menten constant describing the affinity between amino acids and aminoacyl-tRNA synthetase, *K*_M,T,i_ = Michaelis–Menten constant describing the affinity between unaminoacylated tRNAs and aminoacyl-tRNA synthetase, *n* = number of tRNA isoacceptors. (**E**) Correlation of proteome abundance between the measurement (16) and simulation using the prior model (*n* = 100 cells). Each protein is represented by a gray dot. Aminoacyl-tRNA synthetases are highlighted with blue circles. Solid black diagonal represents the *y* = *x* line, and the dashed black lines indicate one order of magnitude above and below the diagonal. (**F**) Comparison of distribution of protein abundance for three representative aminoacyl-tRNA synthetases—CysRS (left), AlaRS (middle), and ThrRS (right)—shown as probability densities. Simulated abundances are from the prior model (*n* = 100 cells, blue) and the measured distributions were scaled from (17) (yellow). Full set of comparisons is shown in Supplementary Figure S2. All cells were simulated in aerobic growth in M9 Minimal Media supplemented with 0.4% glucose at 37°C. Simulations of the prior model represent 10-generation long lineages initialized at 10 random seeds (total of *n* = 100 cells). Simulations in the updated model using the measured aminoacyl-tRNA synthetase *k*_cat_s represent 2-generation long lineages initialized at 50 random seeds (total of *n* = 100 cells). Descriptions of the analyses performed in this figure can be found in Supporting Materials, Section 4.

As mentioned above, Jakubowski and Goldman proposed that measured tRNA aminoacylation rates are not high enough to adequately support cell growth (3). However, this proposal was never fully explored. Thus, we curated 131 *k*_cat_ measurements from 81 studies representing all 20 aminoacyl-tRNA synthetases (Supplementary Table S1) to inform the simulated rates of tRNA aminoacylation in our model. To make a conservative first estimate, we selected the largest measured *k*_cat_s from our data compilation and described the aminoacyl-tRNA synthetase enzymes as fully saturated so that the maximum enzyme flux could be calculated as *v*_max_ = *k*_cat_[*E*], where [*E*] is the concentration of aminoacyl-tRNA synthetases and is retrieved from the cellular content of the simulated cell at each time step. We then ran 50 randomly-seeded simulations in the updated model introduced by the current study, each two generations long (100 simulations in total), representing aerobic growth in M9 Minimal Media supplemented with 0.4% glucose at 37°C. This condition has previously been measured to have a 44-min doubling time (14,15). We found that, in contrast to the prior model (with unlimited tRNA aminoacylation), which showed an average doubling time of 48.8 min and agreed well with the 44-min measurement (two-tailed *P*-value = 0.26 calculated from the *z*-score for the 44-minute measurement = 1.12), the simulations that incorporated the measured *k*_cat_s resulted in a 3.1-fold increase in average doubling time to 150 minutes (two-tailed *P*-value = 1.2 × 10^−6^, *z*-score = 4.86) (Figure 2A). Moreover, 9% of the cell simulations reached the 3-h upper limit of simulation time, at which point our model automatically halts simulations as a control on computational resources. Thus, in agreement with Jakubowski and Goldman’s assertion, the whole-cell model simulations confirmed that measured *k*_cat_s were incompatible with measured doubling times.

We hypothesized that the increased doubling times were associated with decreased protein biomass. Accordingly, we examined the accumulation of protein versus non-protein mass over the cell cycle in a representative simulation (Figure 2B). Although the cell nearly doubled its mass (2.2-fold increase in total dry mass) during its 150.1-min cell cycle, the cellular components did not double in a balanced manner: protein mass lagged behind at a 1.6-fold increase while non-protein mass (DNA, RNA, and small molecules) advanced ahead at a 2.7-fold increase. Expanding our examination to the full set of simulations revealed a consistent under-production of protein (1.6-fold average increase in protein mass, two-tailed *P*-value = 6.6 × 10^−5^ calculated from the *z*-score for the 2-fold expectation for cell doubling = –3.99) and over-production of non-protein components (2.3-fold average increase in non-protein mass, two-tailed *P*-value = 0.16, *z*-score = 1.41), resulting in roughly doubled total dry cell mass (2.0-fold average increase in dry mass, two-tailed *P*-value = 0.99, *z*-score = 0.01) (Figure 2C). This confirmed that the tRNA aminoacylation rates were insufficient for the maintenance of the cellular proteome but sufficient for non-protein components, at least in the short term.

To further investigate the source of the observed growth insufficiency, we considered the mathematical representation of the tRNA aminoacylation reaction (Figure 2D). For this specific analysis, we assumed that tRNAs were aminoacylated at their maximal reaction rates in our prototype, meaning that only two parameters—the rate of catalysis *k*_cat_ and the aminoacyl-tRNA synthetase concentration [*E*]—could be directly responsible for the low protein mass accumulation we observed. We considered each of these parameters in turn. With regard to the [*E*] term, we compared the simulated proteome against a proteomic dataset (16) that was not used in the construction of the model (Figure 2E). With the exception of proteins with low copies per cell (for which small differences appear amplified on a log-log plot), we observed good agreement between the overall simulated proteome and the measurement (coefficient of determination, *R*^2^ = 0.63 for proteins existing at 30 or more copies per cell). In particular, the 17 aminoacyl-tRNA synthetase subunits that were measured appeared within one order of magnitude above and below the diagonal, indicating satisfactory agreement with the proteomic measurements. Having verified the *average* abundance of the aminoacyl-tRNA synthetases with experimental measurements, we next sought to verify their *distributions* against a single-cell protein profiling dataset with single-molecule sensitivity (17), which was also not used in the construction of the model. To be directly comparable with our faster growing cells (44 minutes doubling time, versus 150 minutes in the dataset (17)), we scaled up the reported gamma distribution by aligning the measured means to our simulated means while preserving the shape of the measured distributions (representatives in Figure 2F, full set in Supplementary Figure S2). For all 13 aminoacyl-tRNA synthetases in the measured dataset, their simulated distributions agreed well with corresponding measured distributions. Notably, none of the aminoacyl-tRNA synthetases showed a broader distribution than their measured counterpart, indicating that the simulated number of aminoacyl-tRNA synthetases molecules were not ‘too low’ (which would be the direction of incompatibility that would lead to insufficient tRNA aminoacylation). Considering that separate experimental measurements supported both the averages and distributions of aminoacyl-tRNA synthetases, we concluded that the measured *k*_cat_s must be the primary cause of insufficient tRNA aminoacylation rates that were unable to support maintenance of the cellular proteome, in agreement with Jakubowski and Goldman (3).

### Optimization of aminoacyl-tRNA synthetase kinetic parameters yields quantitative estimates of *k*_cat_s that adequately support cell growth

**Figure 3.**
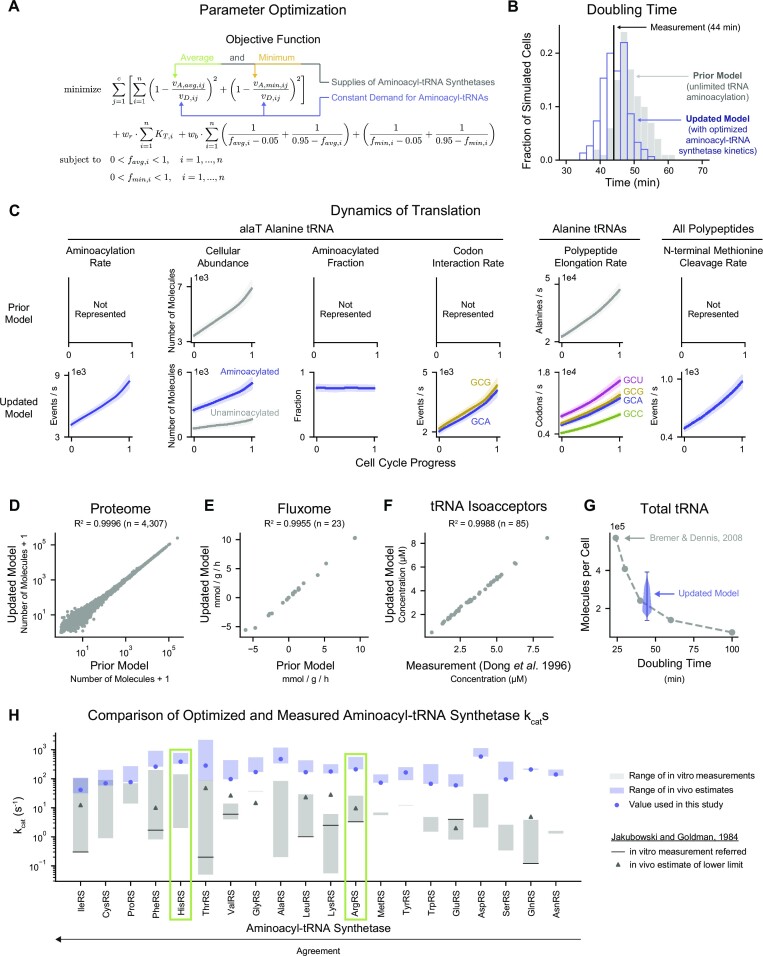
Optimization of aminoacyl-tRNA synthetase kinetic parameters yields quantitative estimates of *k*_cat_s that adequately support cell growth. (**A**) Objective function used to perform optimization of aminoacyl-tRNA synthetase kinetic parameters. The three main components are: steady-state errors (first line), regularization of kinetic parameters (first summation on the second line), and bounds penalties for aminoacylation levels that are too close to either extreme—0 or 1 (second summation on the second line). Each aminoacyl-tRNA synthetase was treated as an independent optimization problem. Notations are: *v*_A,avg,*ij*_ = rate of aminoacylation of *i*th tRNA isoacceptor at average aminoacyl-tRNA synthetase concentrations, *v*_A,min,*ij*_ = rate of aminoacylation of *i*th tRNA isoacceptor at minimum aminoacyl-tRNA synthetase concentrations, *v*_D,*ij*_ = rate of amino acid transfer and release of *i*th tRNA isoacceptor, *K*_T*,i*_ = Michaelis–Menten constant describing the affinity between the unaminoacylated form of the *i*th tRNA isoacceptor and the aminoacyl-tRNA synthetase, *f*_avg,*i*_ = fraction of *i*th tRNA isoacceptor in the unaminoacylated form at average aminoacyl-tRNA synthetase concentrations, *f*_min,*i*_ = fraction of *i*th tRNA isoacceptor in the unaminoacylated form at minimum aminoacyl-tRNA synthetase concentrations, *n* = number of tRNA isoacceptors, *c* = number of growth conditions, *w*_r_ = weight of regularization term, *w*_b_ = weight of bounds penalty. Description of the parameter optimization approach can be found in Supporting Materials, Section 2. (**B**) Distribution of doubling times of cells simulated in the prior model (*n* = 100 cells, gray) and in the updated model when using the optimized aminoacyl-tRNA synthetase kinetic parameters (*n* = 150 cells, blue)—compared to the experimentally measured doubling time (black). (**C**) Comparison of translation dynamics between the prior model (*n* = 100 cells) and updated model when using the optimized aminoacyl-tRNA synthetase kinetic parameters (n=150 cells) during the cell cycle for alanine. Aminoacylation rate, aminoacylated fraction, rate of tRNA-codon interaction, and N-terminal initial methionine cleavage are new representations introduced by the current study. The cellular abundance of tRNAs have been divided into aminoacylated and unaminoacylated fractions in the updated model. The direct polymerization of alanine in the previous model has been replaced with codon-based polypeptide elongation (the four codons of alanine: GCU, GCG, GCA and GCC). Solid lines represent mean behavior and shaded regions indicate one standard deviation above and below the mean. (**D**) Correlation of average protein abundance between the prior model and the updated model when using the optimized aminoacyl-tRNA synthetase kinetic parameters. Each protein (*n* = 4307 total) is represented by a gray dot. (**E**) Correlation of average flux through central carbon metabolism between the prior model and the updated model when using the optimized aminoacyl-tRNA synthetase kinetic parameters. Each reaction (*n* = 23 total) is represented by a gray dot. (**F**) Correlation between the simulated (in the updated model when using the optimized aminoacyl-tRNA synthetase kinetic parameters) and measured (as reported by Dong and colleagues (18)) average concentrations of tRNA isoacceptors. Each tRNA isoacceptor is represented by a gray dot; the mapping between tRNA isoacceptors reported by Dong and colleagues and our simulations is reported in Supporting Materials, Section 4.2.2. (**G**) Distribution of the total number of simulated tRNA molecules per cell in blue (in the updated model when using the optimized aminoacyl-tRNA synthetase kinetic parameters) compared to a report by Bremer and Dennis (19) in gray. **(H)** Comparison of the ranges of aminoacyl-tRNA synthetase activities observed from measurements (gray) and from the parameter optimization (blue). The estimations of the lower limit of aminoacyl-tRNA synthetase activities by Jakubowski and Goldman (gray triangles) and the particular measurements their study compared to (black lines) are also indicated (3). For each aminoacyl-tRNA synthetase optimization problem, the best solution (blue circle) was used for the remainder of this study. HisRS and ArgRS (green boxes) are investigated further in this study. Aminoacyl-tRNA synthetases are ranked by their degree of agreement between the optimized and measured activities. All cells were simulated in aerobic growth in M9 Minimal Media supplemented with 0.4% glucose at 37°C. All simulations in this figure represent 10-generation long lineages initialized at 10 (prior model, total of *n* = 100 cells) or 15 (updated model when using the optimized aminoacyl-tRNA synthetase kinetic parameters, total of n=150 cells) random seeds. Descriptions of the analyses performed in this figure can be found in Supporting Materials, Section 4.

Having determined that the measured aminoacyl-tRNA synthetase *k*_cat_s are primarily responsible for insufficient protein production, we sought to calculate *k*_cat_ values that would support the cell’s demand for protein synthesis. We developed a parameter optimization strategy that treated the kinetics of each aminoacyl-tRNA synthetase as an independent optimization problem that aimed to minimize differences between the rates of tRNA aminoacylation and aminoacyl-tRNA utilization—at both the average and minimum intracellular abundances of aminoacyl-tRNA synthetases—while holding to the principle that the protein content of the cell must grow exponentially and double at roughly the measured doubling time (Figure 3A). The average and minimum concentrations of aminoacyl-tRNA synthetases were estimated from sample simulations produced from our prior model (with unlimited tRNA aminoacylation) (5). During optimization, the estimated average aminoacyl-tRNA synthetase concentrations were held fixed, while the minimum concentrations—due to their variable nature between cells—were allowed to range between the estimated minimum and 0 μM (which is the lowest feasible value) in a 4-value parameter sweep (Supplementary Figure S3) (described more fully in Supporting Materials, Section 2). For each aminoacyl-tRNA synthetase, this process produced a range of *k*_cat_ solutions, of which the candidate *k*_cat_ corresponding to the minimum objective value was identified as the best solution (reported in Supplementary Table S2 and compared to the average measured *k*_cat_ in Supplementary Table S3) and used for the remainder of this study.

Next, we wanted to assess the impact of the optimized *k*_cat_s in the virtual *in vivo* context of our simulations. We therefore replaced the measured *k*_cat_s with their optimized counterparts, relaxed our assumptions about saturation in amino acids and tRNAs shown in Figure 2D (the saturation assumption for ATP was left intact), and performed 150 simulations of 10-generation long lineages (initialized at 15 random seeds) representing aerobic growth in M9 Minimal Media supplemented with 0.4% glucose at 37°C. In contrast to our previous simulations, the average doubling time was found to be to 44.4 min (two-tailed *P*-value=0.90, *z*-score = 0.12) and the population distribution (standard deviation of 3.6 min) returned to levels observed from the prior model (standard deviation of 4.3 min) (Figure 3B).

We also sought to compare the resulting simulation output to our prior model—in particular, as related to translation. Considering our set of 150 simulations further, we compared outputs with the prior model outputs at the tRNA, amino acid and protein levels (Figure 3C). Our comparison identified several improvements in simulation output. First, the prior model was unable to represent tRNA aminoacylation rates, aminoacylated fraction, rates of interactions between aminoacyl-tRNAs and codons (e.g. alanyl-*alaT* tRNA interacts with two codons: GCA and GCG), and N-terminal methionine cleavage rates of nascent polypeptides. Second, representing the tRNA aminoacylation reactions explicitly enabled the allocation of tRNAs into aminoacylated and unaminoacylated forms, in contrast to the prior model, which only represented the unaminoacylated form. Finally, the inclusion of codon-based polypeptide elongation facilitated the observation of the codon reading rate (in contrast to the amino acid reading rate, as represented in the prior model).

Our new simulation outputs were also consistent with the experimental validation benchmarks—the molecular count of each protein (Figure 3D) and the fluxes through reactions in central carbon metabolism (Figure 3E)—that we used in our prior model. We also found that the simulated tRNA abundances, both the isoacceptor-specific concentrations (Figure 3F) and the total number of all tRNAs (Figure 3G), recapitulated their estimates taken from Dong and colleagues (18) and Bremer and Dennis (19), which were respectively used as model inputs. The model improvements also enabled us to compare across tRNA species with greater detail regarding their forms (aminoacylated or not), isoacceptor group, and the full range of their intracellular abundance (Supplementary Figure S4A). This enabled us to compare simulations to a further class of datasets that was not used to parameterize the model: aminoacylated percentage (Supplementary Figure S4B). Four published studies were compared in total; these studies did not uniformly agree with each other (possibly due to variation in strains or experimental conditions), which precludes agreement between the model and the entirety of the data. That said, we observed good agreement with two different studies including Sørensen, in which the charged fraction of *argV*, *leuP*, *leuQ*, *leuT*, *leuV*, and *thrV* in particular matched well with simulations, as did the range of values suggested for *hisR* (20); and Kruger *et al.*, in which *glnU*, *glnW*, *gltT*, *gltU*, *gltV*, *gltW* are inside the simulated ranges, and lysine tRNAs’ aminoacylated percentage measurements overlapped with the upper simulated end (21). A third study (22) exhibited agreement with some of our predictions and not others, but also differed from the other studies in two significant ways: (i) overall the aminoacylated percentages measured tended to be lower than those measured in any other study, often by a substantial amount; and (ii) in some cases the distributions for the aminoacylated percentages ranged higher than 100%, which our model is unable to simulate. The last study (23) was also mixed, with aminoacylated percentages for all tRNAs in the alanine, arginine, glycine, histidine, proline, threonine and tyrosine amino acid families predicted well, for aspartate, isoleucine, and serine not predicted well, and for the rest somewhere in between. In sum, we found that our simulation predictions agreed with at least one of these datasets in most cases, and that our calculated average aminoacylated percentage of 78.8% for tRNAs overall agrees well with all other studies except for the third mentioned. As a result, we concluded that the optimized aminoacyl-tRNA synthetase *k*_cat_s were able to produce simulation outputs that recapitulated typical cell growth.

Equipped with the kinetics-optimized tRNA aminoacylation model that was compatible with the *in vivo* context represented by our simulations, we were able to further pursue the questions raised by the inconsistency between *in vitro* measurements and *in vivo* estimates of aminoacyl-tRNA synthetase activities (3). Jakubowski and Goldman presented their *k*_cat_ estimates as lower limits, and so for their hypothesis to be correct, our estimates would have to be equal or higher—not only than their values, but also than most or all of the experimental measurements. To test this assertion, we compared our estimated *k*_cat_s to the curated experimental data and Jakubowski and Goldman estimates (Figure 3H). For the 10 aminoacyl-tRNA synthetase enzymes Jakubowski and Goldman estimated, our *k*_cat_ estimates (median of 174 s^–1^, blue circles) were on average 9.4-fold (median) higher than their estimates of the lower limit of *in vivo* activity (median of 13.6 s^–1^, gray circles). We also noted that many of the *in vitro* measurements referred to by Jakubowski and Goldman (black lines) fall in the lower range of total curated measurements (gray bars)—and are the absolute minimum measurements reported for IleRS, LeuRS, ArgRS and GlnRS—suggesting that the measurements that have been reported since the 1980s may be higher than older measurements. Taken together, these observations support Jakubowski and Goldman’s assertion that their estimates were indeed lower limits of *k*_cat_s.

We also compared our own estimates to the *in vitro* measurements from our curation, and found that our *k*_cat_ estimations were on average 7.6-fold higher (median) than the highest measurements. Ranking the degree of agreement between our *k*_cat_ estimations (Figure 3H) with either the highest measurement from our curation or the lower limit of *in vivo* activity estimated by Jakubowski and Goldman revealed that 12 of the aminoacyl-tRNA synthetases fall within one order of magnitude of these benchmarks, indicating fair agreement for these enzymes; the remaining eight aminoacyl-tRNA synthetases fell within two orders of magnitude. We chose two representative synthetases (HisRS and ArgRS, highlighted in green) to examine more closely as part of this overall study; the details can be found below.

### Higher, optimized *k*_cat_ values confer robustness in ribosome elongation rate to variability in aminoacyl-tRNA synthetase availability

Pairing two of our previous findings—first, that the optimized *k*_cat_s we calculated were on average 7.6-fold higher than the greatest measurements (Figure 3H), and second, that the intracellular abundance of aminoacyl-tRNA synthetases can be quite variable (Figure 2F and Supplementary Figure S2)—we hypothesized that the calculations made by Jakubowski and Goldman may have been impacted by their reliance on an average aminoacyl-tRNA synthetase concentration rather than the entire range of enzyme concentrations experienced by the cell. In this context, we note two orthogonal factors that can limit protein production. First, the expression of synthetase enzymes is known to be a noisy and stochastic process (17) and can lead to variable enzyme counts that rise and fall, sometimes dramatically, over the course of a cell cycle. Second, the elongation rate of a ribosome on mRNA is thought to have a physical upper limit even when resources are abundant. Taken together, these characteristics suggest that if the aminoacyl-tRNA synthetase *k*_cat_ is not sufficiently high, and the corresponding enzyme counts are lower than the average, then the cell could enter a period of lower protein production. The impact of this lower protein production could be mitigated by a period of higher production at a different time as long as the physical limit on ribosome procession is not surpassed. Thus, we hypothesized that a higher *k*_cat_ confers robustness to variability in aminoacyl-tRNA synthetase enzyme expression.

**Figure 4.**
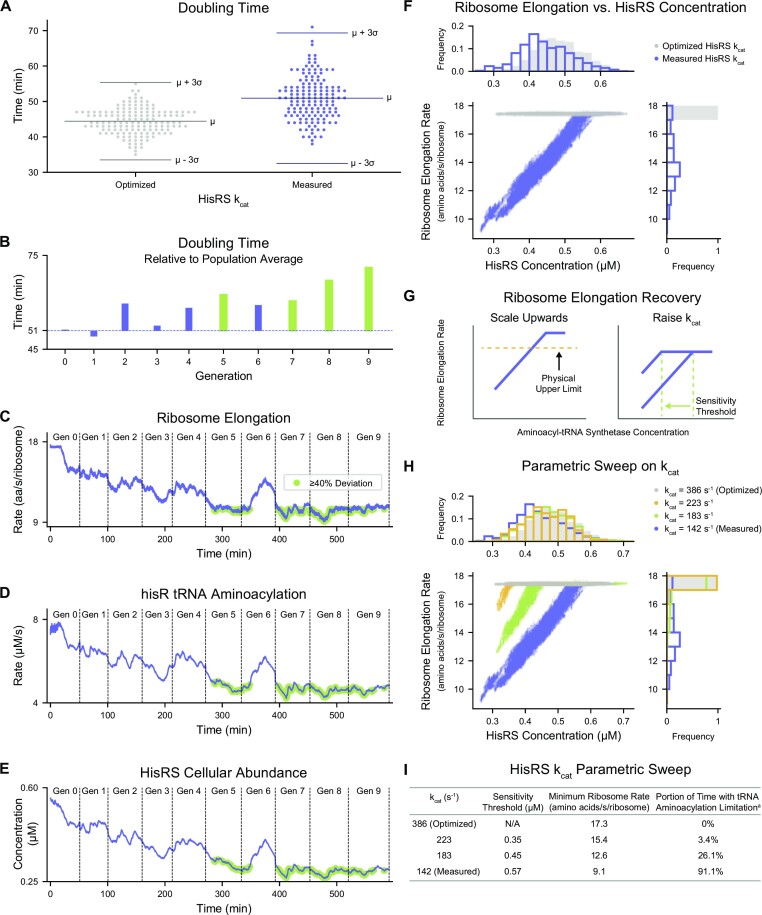
Higher, optimized *k*_cat_ values confer robustness in ribosome elongation rate to variability in aminoacyl-tRNA synthetase availability. (**A**) Distribution of doubling times (rounded to the nearest minute) of cells simulated in the updated model when using the optimized (*n* = 150 cells, gray) and measured (*n* = 150 cells, blue) HisRS *k*_cat_s. The mean (μ) and 3 standard deviations away from the mean (μ ± 3σ) are indicated by the horizontal lines. (**B**) Doubling times of each cell in the 10-generation lineage that includes the cell with the longest doubling time (Variant 3, Seed 12, Generation 9) in the updated model when using the measured HisRS *k*_cat_ in panel A. Doubling times are shown relative to the average of all simulations with the measured HisRS *k*_cat_ (*n* = 150 cells). The top four longest doubling times in this lineage, which are also within the top 10 outliers identified in panel A, are highlighted in green. (**C**) Ribosome elongation rate, (**D**) *hisR* tRNA aminoacylation rate, and (**E**) HisRS concentration during the same 10-generation lineage as panel B. Regions highlighted in green indicate times when the ribosome elongation rate deviated by more than 40% from the expected value for the simulated growth condition (17.5 amino acids per second per ribosome). Vertical dashed lines indicate cell division events. (**F**) Comparison of the relationship between the ribosome elongation rate and HisRS concentration from simulations in the updated model when using the optimized (*n* = 150 cells, gray) and with the measured (*n* = 150 cells, blue) HisRS *k*_cat_s. In the scatter plot, each time step is represented by a dot. The top histogram shows the distribution of HisRS concentrations. The right histogram shows the distribution of ribosome elongation rates. (**G**) Schematic of the two approaches for recovering the average ribosome elongation rate: scaling the relationship upwards (left) or raising the *k*_cat_ (right). The anticipated relationship between ribosome elongation rate and aminoacyl-tRNA synthetase is depicted in blue. The physical upper limit of ribosome procession given the growth condition is depicted in yellow, and the anticipated change in sensitivity threshold is indicated in green. (**H**) Comparison of the relationship between ribosome elongation rate and HisRS concentration from simulations in the updated model when using the optimized HisRS *k*_cat_ = 386 s^–1^ (*n* = 150 cell, gray), *k*_cat_ = 223 s^–1^ (*n* = 100 cells, yellow), *k*_cat_ = 183 s^–1^ (*n* = 100 cells, green), and the measured HisRS *k*_cat_ = 142 s^–1^ (*n* = 150 cells, blue). In the scatter plot, each time step is represented by a dot. The top histogram shows the distribution of HisRS concentrations. The right histogram shows the distribution of ribosome elongation rates. (**I**) Table of characteristics of HisRS *k*_cat_ parametric sweep performed in panel H. (a) The portion of time with tRNA aminoacylation limitation was calculated as the portion of time steps displaying a ribosome elongation rate that was less than the minimum observed ribosome elongation rate in the simulations performed in the Updated Model when using the optimized aminoacyl-tRNA synthetase kinetic parameters (gray). All cells were simulated in aerobic growth in M9 Minimal Media supplemented with 0.4% glucose at 37°C. All simulations in this figure represent 10-generation long lineages initialized at 15 (when using the optimized and measured HisRS *k*_cat_, total of *n* = 150 cells each) or 10 (when using the intermediate HisRS *k*_cat_ values 223 s^–1^ and 183 s^–1^, total of 100 cells each) random seeds in the updated model. Descriptions of the analyses performed in this figure can be found in Supporting Materials, Section 4.

To test this hypothesis, we focused on histidine and performed simulations to determine whether the full range of possible HisRS concentrations can adequately support cell growth when the *k*_cat_ corresponds to measured values. HisRS was chosen for having an estimated *k*_cat_ that showed relatively fair agreement with the highest measurement (ranked fifth for agreement in Figure 3H), and for being a relatively simple system, aminoacylating a single tRNA isoacceptor that reads two codons. We therefore reduced the HisRS *k*_cat_ 2.7-fold from its optimized value (386 s^–1^) to the highest measured value (142 s^–1^) while all other parameters were held constant, and performed 150 simulations (of 10-generation long lineages initialized at 15 random seeds). We found that overall, simulating with the measured HisRS *k*_cat_ led to a slightly increased average doubling time (mean = 50.9 min, standard deviation = 6.1 min) compared to simulations using the optimized *k*_cat_ (mean = 44.4 minutes, standard deviation = 3.6 min) (Figure 4A). Despite the broader distribution, we also observed the presence of a rare (1 out of 150) slower-growing cell with a doubling time that exceeded three standard deviations beyond the mean (where the empirical rule of statistics regards three standard deviations to account for 99.7% of the data in a normal distribution). It therefore seemed that the relatively modest decrease in HisRS *k*_cat_ (compared to other aminoacyl-tRNA synthetases) generally led to slowed growth, but could also lead to rare instances of extremely slowed growth.

To determine whether the rare slower-growing cell was simply a statistical anomaly, or more importantly whether the reduced *k*_cat_ was impacting our simulations, we examined the specific lineage that produced the top outlier—a cell that took 71.1 minutes to complete its cell cycle (Figure 4B–E). In the context of its 10-generation long lineage, the outlier cell occurred in Generation 9 and was preceded by mother cells with increasingly lengthening doubling times (Figure 4B) relative to the population average of 50.9 min (Figure 4A). We thus determined that the lineage experienced disruptions to typical growth, particularly during Generations 5, 7, 8 and 9 (which are all members of the top 10 outliers identified in Figure 4A), that required further examination.

Following our overall hypothesis as described above, we speculated that the translation machinery and its resources might be associated with the disruptions to typical growth. To investigate the productivity of the translation machinery, we examined the ribosome elongation rate (Figure 4C), the aminoacylation rate of *hisR* tRNA (Figure 4D), and the abundance of HisRS (Figure 4E), all in the same lineage. While ribosome elongation began at a stable rate of 17.5 amino acids per second, it also deviated by more than 40% during Generations 5 through 9 (highlighted in green), and reached a minimum of 9.1 amino acids per second in Generation 8 (Figure 4C). During the same window of time (highlighted in green), *hisR* tRNA aminoacylation was nearly halved from an average starting rate of 7.6 μM/s to a minimum of 4.2 μM/s at Generation 7 and reached a comparably low value of 4.3 μM/s at Generation 8 (Figure 4D). Similarly, HisRS concentration visited the lowest 23.5% of its dynamic range (0.26–0.33 μM) during this time, with the minimum occurring in Generation 8 at 478 min (Figure 4E). The coordinated quality of these disruptions throughout the translation machinery suggested a propagation of inadequate capacity to charge *hisR* tRNAs with histidines, and that this insufficiency was originating from HisRS visiting the lower extreme of its dynamic range.

We next examined the correlation between HisRS concentrations and ribosome elongation rates during each time step of all simulated cells (Figure 4F). Whereas the optimized HisRS *k*_cat_ simulations demonstrated stable rates of ribosome elongation, independent of HisRS concentration, the measured HisRS *k*_cat_ led to a dramatic drop in the ribosome elongation rate for HisRS concentrations below 0.59 μM, reaching the previously observed (Figure 4C) minimum of 9.1 amino acids per second per ribosome. This region of tRNA aminoacylation limitation (taken to be when the ribosome elongation rate was less than 17.3 amino acids per second per ribosome, which is the minimum observed in the optimized simulations) accounted for 91.1% of the time steps. These trends suggested two predominant characteristics of the relationship between aminoacyl-tRNA synthetase concentrations and ribosome elongation rates: the previously described physical upper limit on the procession of ribosomes (as informed by the availability of cellular resources in the particular growth condition) and a sensitivity threshold at low aminoacyl-tRNA synthetase concentrations in the measured *k*_cat_ case at which the system switches from maximum to lower elongation rates.

In considering how to ensure that the average ribosome elongation rate is maintained across the entire dynamic range of aminoacyl-tRNA synthetases, we identified two potential approaches, both of which are illustrated schematically in Figure 4G. One approach would be to scale the entire relationship curve between the ribosome elongation rate and aminoacyl-tRNA synthetase concentration upwards, including—and most importantly—the maximum elongation rate (Figure 4G, left panel). We recognized that this approach would compromise the physical upper limit of ribosome procession, which is thought to be well-established (19), and that obeying the physical upper limit would make it difficult for the average ribosome elongation rate to ‘catch up’ after experiencing a significant decrease. The second approach would be to raise the *k*_cat_ (Figure 4G, right panel)—which is the sole term in Figure 2D that can counteract low aminoacyl-tRNA synthetase concentrations, [*E*]. We anticipated that this approach would decrease the sensitivity threshold so that the entire dynamic range of the aminoacyl-tRNA synthetase can be supported by the *k*_cat_ (Figure 4G, right panel).

To test this hypothesis, we performed a parametric sweep on the HisRS *k*_cat_ between the experimentally measured (142 s^–1^) and our optimized (386 s^–1^) values, and performed 100 simulations (of 10-generation long lineages initialized at 10 random seeds) at each new sweep value (Figure 4H and I). As the *k*_cat_ increased, the sensitivity threshold gradually decreased from 0.57 μM (measured *k*_cat_), to 0.45 μM (*k*_cat_ = 183 s^–1^), to 0.35 μM (*k*_cat_ = 223 s^–1^) to nonexistent at the optimized *k*_cat_. Simultaneously, the minimum ribosome elongation rate increased from 9.1 to 17.3 amino acids per second per ribosome and the portion of time spent with tRNA aminoacylation limitation decreased from 91.1% to 0%. We thus concluded that the higher, optimized *k*_cat_ confers robustness of ribosome elongation rate—and correspondingly, to normal cellular physiology and growth—with respect to variability in aminoacyl-tRNA synthetase expression.

### Insufficient ArgRS kinetic capacity leads to catastrophic impacts on cellular phenotype via arginine biosynthesis due to abrogated expression of ArgA

**Figure 5.**
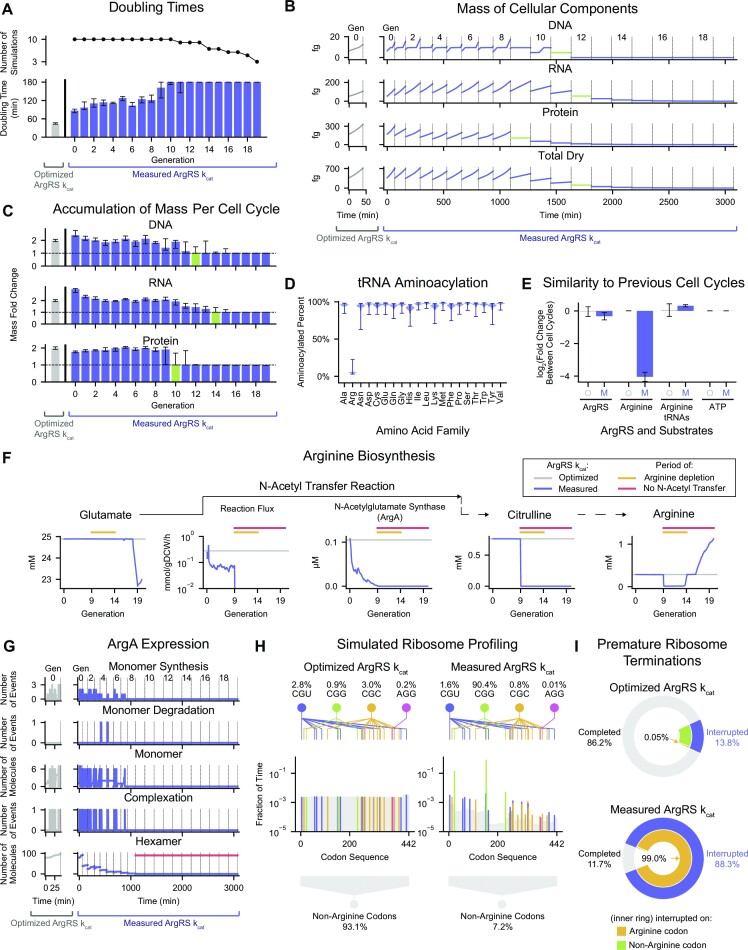
Insufficient ArgRS kinetic capacity leads to catastrophic impacts on cellular phenotype via arginine biosynthesis due to abrogated expression of ArgA. (**A**) Number of viable simulations (top) and doubling times (bottom) at each generation. Doubling times of cells simulated in the updated model when using the measured ArgRS *k*_cat_ are shown in blue and compared to the population average when using the optimized *k*_cat_ in gray (*n* = 150 cells). Error bars indicate the interquartile range. (**B**) Mass of DNA, RNA, protein and total dry cell mass in a representative 20-generation lineage simulated in the updated model when using the measured ArgRS *k*_cat_ (Variant 4, Seed 8) are shown in blue and compared to a representative cell simulated using the optimized *k*_cat_ (Variant 0, Seed 8, Generation 0) in gray. Green highlights indicate cell cycles when cessation of mass accumulation occurred. Vertical dashed lines indicate cell division events. Values across the top indicate the generation number. (**C**) Accumulation of DNA, RNA and protein mass during each cell cycle. The fold change in mass (calculated as the final mass divided by the initial mass of each cell cycle) in the updated model when using the measured ArgRS *k*_cat_ is shown in blue and compared to the population average using the optimized *k*_cat_ in gray (*n* = 150 cells). Error bars indicate the interquartile range. Green highlights indicate cell cycles when cessation of mass accumulation occurred. Horizontal dashed lines represent the expectation of mass doubling at each cell cycle when experiencing exponential growth. (**D**) Distribution of tRNA aminoacylated percent prior to cessation of protein mass accumulation for each amino acid family in simulations in the updated model when using the measured ArgRS *k*_cat_ (*n* = 106 cells). Error bars indicate the full range of observed values. (**E**) Similarity of the intracellular abundances of ArgRS, arginine, unaminoacylated arginine tRNAs, and ATP to previous cell cycles in simulations in the updated model when using the optimized (labeled ‘O’, gray) and measured (labeled ‘M’, blue) ArgRS *k*_cat_s. Fold change was calculated by dividing the mean concentration of a given cell cycle by the mean concentration of the previous cell cycle. For simulations using the measured ArgRS *k*_cat_, only cell cycles immediately preceding and following division events exhibiting the cessation of protein mass accumulation were included in this analysis (*n* = 20 cells). For simulations using the optimized ArgRS *k*_cat_, which exhibited no protein cessation events, all cell cycles were included (*n* = 150 cells). Error bars indicate one standard deviation above and below the mean. (**F**) Characteristics of key small molecules, enzymes, and reaction fluxes in the arginine biosynthesis pathway in simulations in the updated model when using the optimized (gray) and measured (blue) ArgRS *k*_cat_s in the same lineage as panel B. For simulations using the measured ArgRS *k*_cat_, blue lines represent the moving average calculated using a window size of 10 min. For simulations using the optimized ArgRS *k*_cat_, gray lines represent the median value calculated from all 10 generations of the lineage (Variant 0, Seed 8). On all subplots, the period of arginine depletion is indicated by the yellow line, and the period of absence of the *N*-acetyl transfer reaction is indicated by the red line. The solid arrow indicates that glutamate is directly consumed by the *N*-acetyl transfer reaction, while the dashed arrows indicate the existence of intermediary reactions that are not shown. (**G**) Synthesis, degradation, complexation, and hexamer abundance of ArgA in simulations in the updated model when using the optimized (gray) and measured (blue) ArgRS *k*_cat_s in the same lineage as panel B. Monomer synthesis, monomer degradation, and complexation are shown as the number of events per (2-s) time step. Monomer and hexamer abundances are shown as the number of molecules at each time step. The period of ArgA hexamer absence is indicated by the red line. Vertical dashed lines indicate cell division events. Values across the top indicate the generation number. (**H**) Simulated ribosome profiling experiment of codon identities observed on open A sites of ribosomes on *argA* mRNAs in the updated model when using the optimized (n=150 cells, left) and measured (*n* = 171 cells, right) ArgRS *k*_cat_s. The codon sequence of *argA* is numbered from 0 (start codon) to 442 (the final sense codon). Colors indicate arginine codons (CGU in blue, CGG in green, CGC in yellow, and AGG in pink) and gray indicates non-arginine codons. Visualizations above and below summarize the fractions of all ribosome observations that were located on arginine (top) and non-arginine (bottom) codons. (**I**) Fraction of ribosomes initiated on *argA* mRNAs that were terminated prematurely in the updated model when using the optimized (*n* = 150 cells, top) and measured (*n* = 171 cells, bottom) ArgRS *k*_cat_s. Outer ring compares the percentage of ribosomes that successfully completed translation of *argA* mRNAs (gray) to those that were prematurely interrupted (blue). Inner ring compares the percentage of interruptions that occurred on arginine codons (yellow) to those that occurred on non-arginine codons (green). All cells were simulated in aerobic growth in M9 Minimal Media supplemented with 0.4% glucose at 37°C. Simulations of the updated model when using the optimized ArgRS *k*_cat_ represent 10-generation long lineages initialized at 15 random seeds (total of *n* = 150 cells). Simulations of the updated model when using the measured ArgRS *k*_cat_ were initialized as 20-generation long lineages from 10 random seeds, of which *n* = 171 viable cells were studied. Descriptions of the analyses performed in this figure can be found in Supporting Materials, Section 4.

Having considered the measured *k*_cat_ in the case of HisRS, we next performed a similar analysis with a more complicated synthetase example, ArgRS, which aminoacylates seven tRNA isoacceptors that collectively read six codons. We reduced the ArgRS *k*_cat_ 8.1-fold from its optimized value (210 s^–1^) to the highest measured value (26 s2^–1^) while all other parameters were held constant, and attempted to perform 10 lineage simulations, each of which were 20-generations long. We found that 7 of them encountered prematurely terminated cell cycles before all generations were simulated, resulting in fewer viable simulations as the generation number increased (Figure 5A, top). Even in the viable simulations, the lower (measured) ArgRS *k*_cat_ led to a nearly 2-fold increase in doubling time in the first generation compared to simulations performed using the optimized ArgRS *k*_cat_ (Figure 5A, bottom). The doubling time continued to increase in subsequent generations until our three hour doubling time limit was reached. Thus, using the measured ArgRS *k*_cat_ was more detrimental to cell growth than using the measured HisRS *k*_cat_.

To investigate the cause of the increased doubling times, which suggested a slowed accumulation of cellular material, we examined the production of cell mass at the DNA, RNA, and protein levels in a single lineage from our simulation set (Figure 5B). Although we anticipated (and saw) a slight reduction in protein production (compared to the optimized ArgRS *k*_cat_ case) and an increase in RNA production (consistent with Figure 2B and C) starting at Generation 0, we were surprised to see a complete absence of protein accumulation starting at Generation 9, followed by cessation of DNA (Generation 11) and RNA (Generation 12) accumulation as well. Once production had stopped, the mass of these cellular components halved at each subsequent cell division event, as would be expected. To determine whether our observations in this single simulation extended to the entire set of simulations, we calculated the fold change in cell mass as a function of the cell cycle number (Figure 5C). While the optimized ArgRS *k*_cat_ simulations exhibited a two-fold increase in all major cellular components, the measured ArgRS *k*_cat_ simulations revealed a consistent trend of lineages that were unable to accumulate protein mass starting at Generation 10, then DNA at Generation 12, and then RNA at Generation 14 (green bars).

Since the factor(s) limiting protein production at, for most lineages, Generation 10 may have been related to an insufficient supply of aminoacyl-tRNAs, it seemed that the translation machinery required further attention. To assess whether aminoacyl-tRNAs were limiting the production of protein, we considered the distribution of aminoacylated fractions of tRNAs grouped by amino acid family for all lineages up to the generation when protein mass accumulation halts (Figure 5D). For almost all of the amino acid families, the average aminoacylated percent varied from 91.3% (histidyl-tRNAs) to 97.8% (methionyl-tRNAs). In contrast, arginine tRNAs showed a much lower average aminoacylation at 5.1%, strongly suggesting that low aminoacylation of arginine tRNAs was the consistent failure mode of using the measured ArgRS *k*_cat_.

This was a surprise: although decreasing the ArgRS *k*_cat_ from its optimized to measured value was the original perturbation responsible for the reduced aminoacylation of arginine tRNAs, the model *k*_cat_ was held at a constant value for each simulation after being set. This means that the *k*_cat_ value, although low in these simulations, cannot directly explain the sudden halt of protein accumulation in Generation 10 (Figure 5C). To unearth the mechanism of this cessation, we examined other potential causative factors, beginning with the four main concentration variables in our aminoacylation rate equation (Figure 2D): ArgRS, arginine, unaminoacylated arginine tRNAs, and ATP. We compared the concentrations of these four molecules immediately preceding and following the cell cycle division event when protein mass accumulation halts (Figure 5E). We observed that the log_2_ ratios of cellular abundance pre- and post-cessation of protein mass accumulation for ArgRS, arginine tRNAs and ATP were all close to zero, and agreed with simulations performed using the optimized ArgRS k_cat_. In contrast, the pool of free arginine showed a 16.2-fold decrease in cellular abundance (two-tailed *P*-value < 1 × 10^−6^ calculated from the z-score for the optimized ArgRS *k*_cat_ average = 81.5). These results indicated that the ArgRS *k*_cat_’s impact on protein synthesis was in fact not due directly to translation, but instead related to a previously unknown connection to arginine metabolism.

Thus, to investigate the decreased cellular abundance of arginine, we first examined the arginine biosynthesis pathway for enzyme and small molecule concentrations as well as metabolic fluxes (Figure 5F) for the same lineage as Figure 5B. Consistent with the previously observed cessation in protein mass accumulation in Figure 5B, arginine depletion began at Generation 9 and continued to the start of Generation 14 (highlighted by the yellow line above the trace in all subplots of Figure 5F). In contrast, glutamate did not exhibit a change during the arginine depletion period, suggesting that the cause of arginine depletion was downstream of glutamate. Indeed, we saw that the N-acetyl transfer reaction that consumes glutamate showed a sudden decrease at Generation 9 (indicated by the red line on this and subsequent panels) corresponding to the start of arginine depletion. Consequently, citrulline, a downstream metabolite, exhibited a depletion period also beginning at Generation 9 and continuing through the rest of the simulation. Since the flux through the *N*-acetyl transfer reaction is impacted by the abundance of the *N*-acetylglutamate synthase (ArgA) enzyme, we examined the cellular abundance of *N*-acetylglutamate synthase and observed a gradual depletion from 0.11 μM at the start of the simulation to 0 μM at Generation 9, after which it remained absent from the lineage for several generations. (We note that the recovery of arginine at Generation 14 is caused by the ribosome elongation rate decreasing to zero, which causes amino acids that had experienced depletions to begin re-accumulating.)

The impact of the ArgRS *k*_cat_ on the expression of ArgA was a further surprise, and begged the question: how often is ArgA being expressed in the reduced ArgRS *k*_cat_ simulations? We investigated the synthesis, degradation, cellular abundance, and complexation of the ArgA monomer subunits in addition to the number of complete N-acetylglutamate synthase hexamers in the cell (Figure 5G). Whereas simulating with the optimized ArgRS k_cat_ exhibited robust expression of *argA* into ArgA monomers, simulations using the measured ArgRS *k*_cat_ showed only one to two synthesis events per time step during Generation 0 through 7. At both ArgRS *k*_cat_ values, degradation played a minimal role and complexation generally occurred when the cell had accumulated six subunits. Consequently, although both simulations began with around 80 copies per cell, cells simulated with the measured ArgRS *k*_cat_ did not express *argA* sufficiently and quickly depleted their supply of N-acetylglutamate synthase hexamers. Although the low cellular abundance of *N*-acetylglutamate synthase hexamers was tolerable up through Generation 8, by Generation 9 the number of N-acetylglutamate synthase hexamers fell to zero (red line above the trace), causing the inability to accumulate protein observed in Figure 5B.

To investigate the source of low ArgA expression in our measured ArgRS k_cat_ simulations, we simulated a ribosome profiling experiment. We monitored the identity of the unoccupied A site codon of ribosomes processing on *argA* mRNAs (Figure 5H). The simulations performed with the optimized ArgRS *k*_cat_ showed an even distribution of time spent across the transcript, suggesting a steady procession of ribosomes (left panel of Figure 5H). In contrast, simulations using the measured ArgRS *k*_cat_ showed a highly irregular ribosome profile, suggesting frequent interruptions to ribosome procession. Indeed, decreasing the ArgRS *k*_cat_ from its optimized to measured value caused the percentage of ribosomes found on arginine codons to increase from 6.9% to 92.8% (90.4% on CGG, 1.6% on CGU, 0.8% on CGC, and 0.01% on AGG). We found that this increased pausing on arginine codons led to premature termination of ribosomes and reduction of protein products (Figure 5I): whereas 86.2% of ribosomes initiated on *argA* mRNAs completed in simulations using the optimized ArgRS *k*_cat_, only 11.7% did so in the measured ArgRS *k*_cat_ case. Furthermore, of these interrupted ribosomes, 98.97% were interrupted while waiting on arginine codons (whereas only 0.05% of ribosomes interrupted in the optimized ArgRS *k*_cat_ simulations experienced those interruptions on arginine codons). These results indicated that the greater number of ribosomal pauses on arginine codons led to higher rates of interruption (and equivalently lower rates of completion and thereby protein synthesis) of ArgA monomers.

Prompted by these findings, we investigated where the ribosomes were located on the *argA* mRNA when they were prematurely terminated, and found two regions of high frequency with regard to premature ribosome termination (Supplementary Figure S5A). First, the tandem CGG codons at codon positions 153 and 154 (0-indexed positions) accounted for 76.9% of the premature ribosome terminations, indicating that the tandem CGG codons correspond to the site where the majority of missed ArgA expression opportunities occur. The second highest site of premature ribosome termination occurred at a CGG codon at position 23, which accounted for 13.5% of the terminations. Examination of the *argA* sequence showed that these were the first three CGG codons encountered by the ribosome during translation (Supplementary Figure S5B). These findings suggest that the first (or only) instances of CGG codons along an mRNA’s translatable codon sequence may be sites that are prone to premature ribosome termination—and especially so if the CGG codons are in tandem.

This result prompted us to examine whether other proteins with tandemly arranged CGG codons also experienced a reduction in expression. First, we searched for tandem CGGs or other rare arginine codons (AGA or AGG) in all of the proteins in *E. coli*. Of 4,307 total proteins, tandem CGGs were found in 114 proteins (listed in Supplementary Table S4), tandem AGAs were found in 35 proteins, and tandem AGGs were found in 5 proteins (Supplementary Figure S5C). Three proteins were found to contain both tandem CGGs and AGAs, 62 proteins contained no arginine codons, and the remaining 4,094 proteins contained arginine codons that were not tandem arrangements of CGGs, AGAs or AGGs. To investigate whether the presence of tandemly arranged rare arginine codons in a protein’s codon sequence impacts its expression, we compared the expression of these genes in simulations using the optimized ArgRS *k*_cat_ to those using the measured ArgRS *k*_cat_ (which would reduce the availability of arginine-charged arginyl-tRNAs, Supplementary Figure S5D). We found that proteins containing tandem CGG codons in particular were significantly under-expressed, with a reduction of roughly 16.7-fold (two-tailed *P*-value = 4.7 × 10^−3^ calculated from the *z*-score for the median (0.06) observed in the ‘CGG, CGG’ = –2.83; treats ‘No Arginines’ group as the reference distribution). As a final test, we performed simulations in which the CGG tandem in *argA* was changed to a CGU tandem, and again ran simulations using either the optimized or experimentally measured ArgRS k_cat_s (Supplementary Figure S5E). These sequence changes had no significant impact on ArgA expression with respect to the optimized parameter strain simulations, but increased ArgA expression by more than 20-fold when the experimentally-measured parameters were used (two-tailed p-value = 2.6 × 10^−52^ calculated from the z-score for the mean (276.4) observed in the ‘CGU, Measured ArgRS *k*_cat_’ distribution = 15.22; treats ‘CGG, Measured ArgRS *k*_cat_’ as the reference distribution, Supplementary Figure S5E). Taken together, these results strongly supported a causal relationship between the CGG tandem codon and downstream gene expression—not only for *argA*, but also other genes.

**Figure 6.**
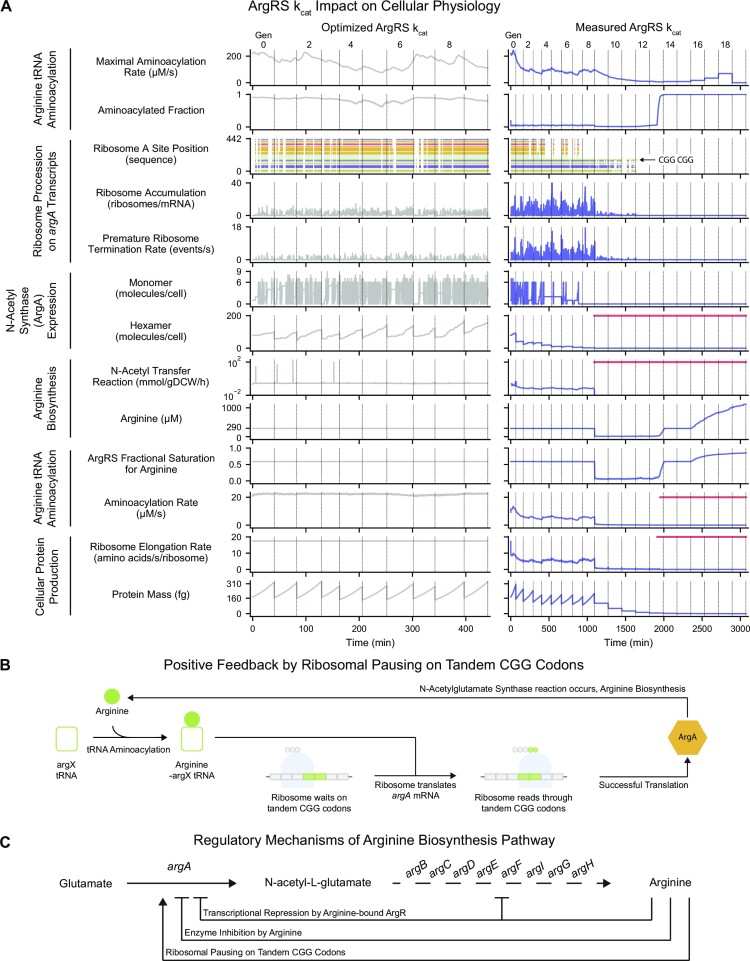
The link between arginine tRNA pools and ArgA expression predicts a positive feedback mechanism regulating the arginine biosynthesis pathway. (**A**) Holistic view of the impact of ArgRS aminoacylation capacity on cell growth in representative lineages simulated in the updated model when using the optimized (Variant 0, Seed 8, all 10 generation on the left) and measured (Variant 6, Seed 8, all 20 generations on the right) ArgRS *k*_cat_s. From top to bottom: 1) maximal aminoacylation rate of arginine tRNAs, 2) aminoacylated fraction of arginine tRNAs, 3) position (and codon identities by color: CGU in blue, CGG in green, CGC in yellow, AGG in pink, and non-arginine codons in gray) of ribosomes on *argA* mRNAs, 4) number of ribosomes per *argA* mRNA, 5) premature termination rate of ribosomes from *argA* mRNAs, 6) number of ArgA monomers, 7) number of ArgA hexamers (*N*-acetylglutamate synthase), 8) flux through the N-acetyl transfer reaction in the arginine biosynthesis pathway, 9) concentration of arginine, 10) fractional saturation of ArgRS for its substrate arginine, calculated as $\frac{[A]}{(K_{M,A} + [A])}$, where [*A*] is the concentration of arginine and *K*_M,A_ is the Michaelis–Menten constant describing the affinity between arginine and ArgRS, 11) aminoacylation rate of arginine tRNAs, 12) ribosome elongation rate, and 13) total protein mass. Vertical dashed lines indicate cell division events. Values across the top indicate the generation number. Red lines in rows 7 (ArgA hexamers), 8 (*N*-acetyl transfer reaction), 11 (aminoacylation rate of arginine tRNAs), and 12 (ribosome elongation rate) indicate the period of time when the quantity shown (number of molecules, reaction flux, or rate) had a value of 0. All cells were simulated in aerobic growth in M9 Minimal Media supplemented with 0.4% glucose at 37°C. (**B**) Positive feedback by ribosomal pausing on tandem CGG codons on *argA* mRNAs. Arginine availability impacts the aminoacylation of *argX* tRNAs with arginine, which in turn influences the ability of ribosomes to read through tandem CGG codons on *argA* mRNAs. Successful translation of *argA* mRNAs leads to the production of ArgA, the first enzyme in the arginine biosynthesis pathway. (**C**) Regulatory mechanisms characterizing the arginine biosynthesis pathway. The propagating impact of using the lower, measured ArgRS *k*_cat_ on cell physiology suggests the presence of a regulatory positive feedback link through ribosomal pausing on tandem CGG codons when translating the mRNA transcript for the arginine biosynthetic enzyme ArgA. Descriptions of the analyses performed in this figure can be found in Supporting Materials, Section 4.

More broadly, these observations served to build a holistic picture of the reduced ArgRS *k*_cat_’s unanticipated impact on cell growth via arginine biosynthesis—a picture that connects aminoacylation, the pausing of individual ribosomes, translational interruption to the synthesis of *N*-acetylglutamate synthase, arginine biosynthesis, back to aminoacylation (via the arginine saturation fraction, rather than the *k*_cat_) and finally to cessation in protein mass accumulation (Figure 6A). Compared to the optimized ArgRS *k*_cat_, the maximal aminoacylation rate of arginine tRNAs reduced 1.6-fold from 154 μM/s (average of all 10 generations) to 98.0 μM/s (average of Generations 0 through 8) then dwindled to 8.4 μM/s during Generations 12 through 15. The aminoacylated fraction, which maintained stable levels at 84.2% in the optimized ArgRS *k*_cat_, dropped to near zero values in the measured ArgRS *k*_cat_ until Generation 13 when the replenished arginine pool and slowed ribosome elongation rate (depicted in downstream rows in Figure 6A) caused tRNA pools to accumulate in their aminoacylated forms. Simultaneously, ribosomes translating *argA* mRNAs were observed to terminate prematurely during their procession, as indicated by the ribosomes on the beginning portion of the *argA* mRNA frequently being unable to translate beyond the tandem CGG codons at sequence positions 153 and 154 compared to the optimized ArgRS *k*_cat_ case (also apparent in Figure 5H), where the vast majority of ribosomes continued to translate beyond position 154 to the end of the transcript. Accordingly, the accumulation of ribosomes on *argA* mRNAs increased from an average of 1.9 ribosomes per transcript in the optimized ArgRS case to 3.5 (average of Generations 0 through 8)—and a maximum of 40 in the beginning of Generation 5—ribosomes per transcript in the measured ArgRS case. This trend extended into the premature ribosome termination rate, which increased from an average rate of 2.5 × 10^−2^ s^–1^ in the optimized ArgRS simulations to 3.6 × 10^−2^ s^–1^ (average of Generations 0 through 8)—and a maximum of 17.5 s^–1^ in the beginning of Generation 8—in the measured ArgRS case. Consequently, the cellular abundance of ArgA monomers reduced 1.7-fold from an average of 2.8 monomers when using the optimized ArgRS *k*_cat_ to 1.7 (average of Generations 0 through 7) in the measured ArgRS *k*_cat_ case, followed by a complete absence of ArgA monomers for the remainder of the simulation. These trends carried over to the number of hexamers: an average of 21.4 copies of N-acetylglutamate synthase per cell in the measured ArgRS *k*_cat_ case (average of Generations 0 through 8), compared to the average abundance of 92.1 hexamers in the optimized case. Correspondingly, not only were N-acetylglutamate synthase hexamers unable to accumulate but the hexamer count also halved at each division event, diminishing to just two copies in Generation 8 and zero copies by Generation 9 and onwards (indicated with a red line)—a stark contrast to the optimized ArgRS *k*_cat_ simulations, which showed regular exponential accumulation of the hexamer that resulted in an average of 2.0-fold increases during each generation. The absence of N-acetylglutamate synthase enzymes directly impacted the rate of the N-acetyl transfer reaction: decreasing from an average of 0.31 mmol per gram dry cell weight per hour in the optimized ArgRS *k*_cat_ simulations to 7.8 × 10^−2^ (average of Generations 0 through 8)—and zero flux in Generations 9 and onwards (indicated with a red line)—in the measured ArgRS k_cat_ simulations. As a result, the cellular pool of arginine experienced a period of depletion, decreasing from its steady level of 286.5 μM when using the optimized ArgRS *k*_cat_ to 21.4 μM during Generations 9 through 13 in the measured ArgRS *k*_cat_ simulations. In turn, the decreased arginine pool impacted fractional saturation of the ArgRS enzyme for arginine, which decreased from 0.59 in the optimized ArgRS *k*_cat_ case to 8.7 × 10^−2^ during Generations 9 through 13 in the measured ArgRS *k*_cat_ case. The original perturbation (decreasing the ArgRS *k*_cat_ from its optimized to measured value) and the decreased fractional saturation, together, influenced the aminoacylation rate of arginine tRNAs: decreasing from the steady rate of 22.2 μM/s in the optimized ArgRS *k*_cat_ simulations to 5.8 μM/s during Generations 0 through 8, 0.2 μM/s during Generations 9 through 13, and zero thereafter. At the cellular scale, the reduced aminoacylation rate of arginine tRNAs directly impacted the ribosomal elongation rate, which decreased from a steady rate of 17.5 amino acids per second per ribosome when using the optimized ArgRS *k*_cat_ to 5.6 amino acids per second per ribosome in the measured ArgRS *k*_cat_ case during Generations 0 through 8, 0.3 amino acids per second per ribosome during Generations 9 through 13, and zero thereafter. Finally, the functional consequence of this limiting arginine biosynthetic enzyme presented itself as the cessation of protein mass accumulation starting at Generation 9, first noted in Figure 5B, that exacerbated the slowed growth observed in Figure 5A.

## DISCUSSION

In summary, we investigated the previously reported enigma of aminoacyl-tRNA synthetases: that their measured kinetic capacities may not be sufficient to support protein synthesis in the cell (3). By incorporating a detailed and mechanistic representation of tRNA aminoacylation and codon-based polypeptide elongation into the *E. coli* model, we could examine the measured aminoacyl-tRNA synthetase *k*_cat_s within a simulated context of the living cell. We found that the required *k*_cat_s were up on average 7.6-fold higher than the highest measurements from our curation, and we identified through two aminoacyl-tRNA synthetase case studies—HisRS and ArgRS—that both a modest and substantial decrease in *k*_cat_ from the optimized to measured values can have a significant impact on cell growth. In the case of HisRS, we discovered that ribosome elongation rates were sensitive to aminoacyl-tRNA synthetase variability when using the measured *k*_cat_ and that the higher, optimized *k*_cat_ overcame this sensitivity. The ArgRS study led us to a more complex situation which linked several processes that impair cell growth via a non-intuitive, emergent mechanism.

Overall, our work strongly supports Jakubowski and Goldman’s original assessment that the measured activities of aminoacyl-tRNA synthetases are too low to be compatible with cellular demands for protein synthesis. In fact, we calculated an even higher overall *k*_cat_ requirement, as they anticipated in their original paper. We found that the primary source of this difference between estimates was the dynamic range of aminoacyl-tRNA synthetase concentrations in the single-cell context. While the calculations performed by Jakubowksi and Goldman were informed by average measurements, we found that examining the lowest aminoacyl-tRNA synthetase concentration during the cell’s life is of critical importance, for the simple reason that cell growth must be robust to variance in protein expression.

Other related studies provide insight as to the mechanism of how this higher activity might be achieved. Notably, the phenomenon of macromolecular crowding has been reported to impact the dynamics of molecules in living cells (24–26). For example, Maheshwari and colleagues built a detailed, physics-based model of the *E. coli* cytoplasm and reported that the improved proximity between ternary complexes and ribosomes facilitated by a crowded environment reduced the search distance between ternary complexes and ribosomes, which could enable individual ribosomes to become more productive than widely believed (27). Along these same lines, the use of co-solute crowders has enabled crowded environments to be replicated in *in vitro* settings and has been reported to increase protein synthesis rates when added to cell-free expression systems (28). Spectrophotometric methods have also emerged alongside the traditional method of detecting radiolabeled molecules to determine aminoacyl-tRNA synthetase activities (29,30). One such technique (31) indicated that the addition of tRNA—which is typically absent in traditional PPi exchange assays (32)—promoted continuous amino acid activation and thereby greater detection of PPi release. The role of post-transcriptional modifications of tRNAs has also been noted to impact the identity elements that determine aminoacyl-tRNA synthetase specificity (33). As such, the differences between native (modified) and *in vitro* transcribed (unmodified) tRNAs (34–37) may influence recognition of tRNA substrates by their aminoacyl-tRNA synthetase enzymes. Indeed, Clifton *et al.* found that replacement of m^1^G37-modified tRNA with G37-unmodified tRNA resulted in a 17-fold reduction in aminoacylation (38). Given such findings, we consider the optimized *k*_cat_s presented in this study to be initial estimates; we anticipate that the *E. coli* model will produce more precise predictions as additional measurements are made and subsequently incorporated, and the model thereby develops further over time.

Our most striking result was that the consequence of low ArgRS *k*_cat_ reproducibly routed to the arginine biosynthesis pathway. We anticipated insufficient aminoacyl-tRNA synthetase activities would impact protein synthesis—and that this in turn would reach a variety of functions based on the stochastic nature of gene expression in the cell. This agrees with what we saw in the HisRS case, which showed that a modest (2.7-fold) decrease of aminoacyl-tRNA synthetase activity led to decreases in global ribosome elongation rate that generated simulations of cells that could complete their cell cycles, albeit slowly. In the case of ArgRS, however, the more dramatic 8.1-fold decrease of activity caused a prolonged insufficiency that caused decreased expression of the arginine biosynthetic enzyme ArgA through ribosome pausing at tandem CGG arginine codons, suggesting a regulatory positive feedback link between arginine tRNA pools and the synthesis of arginine itself (Figure 6B).

Reminiscent of the mechanism of ribosome-mediated transcriptional attenuation, the presence of tandem CGG arginine codons in the coding region of ArgA (and possibly other genes listed in Supplementary Table S4) may serve as a sensor of arginyl-tRNA abundance. A potential reason for our simulated cell’s particular sensitivity to the CGG codon identity may be that the CGG codon is only read by its cognate tRNA, *argX* (with anticodon 5’-CCG-3’). Since the *argX* tRNA is the lowest abundant arginine tRNA, both in our simulations and by experimental measurement (18), it may be the most sensitive tRNA to reduced ArgRS k_cat_. In contrast, the CGU codon can be read by 4 different arginine tRNAs—*argQ*, *argV*, *argY* and *argZ*—which are the top 4 most abundant arginine tRNAs with a total mean abundance of 2635 molecules per cell (11.9-fold greater than *argX*).

Interestingly, a corroborative finding was reported by McNulty and colleagues in their study of tandem triplet CGG codons occurring in the coding region for a p27 protease from Herpes Simplex Virus 2 (HSV-2) (39). When expressed in *E. coli*, +1 frameshift events were observed from both the second and third CGG codons in the triplet cluster. Additionally, significant levels of glutamine misincorporation for arginine were reported, which the authors suggest resulted from second base misreading of CGG as CAG. However, coexpression of the *argX* gene eliminated the frameshifts and misincorporation events, and increased expression by up to 7-fold.

Taken together, these findings suggest that the presence of tandem CGG codons located at positions 153 and 154 in the *argA* codon sequence is sensitive to reduced availability of arginine-charged arginyl-tRNAs. As a result, the tandem CGG codons may be able to serve as a site of regulation that responds to reduced arginine availability by increasing the chance of premature ribosome termination. This behavior can be classified as a positive feedback mechanism that responds to high arginine availability by increasing the chance of successful translation events. The predicted presence of this positive feedback mechanism, together with the previously known negative feedback mechanisms provided by both transcriptional repression by arginine-bound ArgR and direct inhibition of ArgA by arginine, prompt us to re-classify the regulation of the arginine biosynthesis pathway as a combination positive-and-negative feedback loop (Figure 6C). Pfeuty and Kaneko have reported that such combination feedback loops confer rapid and reversible responses to changing environments (40), which may be applicable to the cell’s response to arginine depletion.

The model reported in this work could be further strengthened by incorporating richer representations of the translation machinery. Aminoacyl-tRNAs have been reported to bind elongation factor Tu with uniform binding affinities (41). Incorporating a mathematical model of these interactions (such as in (42)) may further elucidate how the delivery of aminoacyl-tRNAs to ribosomes informs the activities of aminoacyl-tRNA synthetases *in vivo*. In addition, representing ribosome procession to a finer resolution would enhance the analyses presented in this study. For example, volume exclusion of polysomes on shared transcripts was captured by Levin and Tuller (7), but is not currently modeled here. Additionally, incorporating measurements of ribosome residence times on codons (43), estimations of translation times at the codon resolution (44), the influence of mRNA and nascent-peptide sequences on elongation dynamics (45), and a mechanistic model of ribosomal frameshifting (46) may further characterize the relationship between aminoacyl-tRNA synthetase kinetic capacities and ribosome elongation rates. Furthermore, the *E. coli* model does not yet represent a detailed kinetic scheme of codon reading by tRNA and release factors, such as demonstrated by Ieong and colleagues (47). Incorporating their model may improve the granularity of ribosome elongation dynamics. Additionally, representing ribosome pausing (at Proline-Proline motifs (48) and at Shine-Dalgarno like sequences (49)) and rescue (by alternative ribosome-rescue factor A (50,51)) may enable new insights regarding the impact of insufficient aminoacyl-tRNAs on ribosome procession. Moving forward, we note that the (p)ppGpp-mediated stringent response in *E. coli* is known to be activated by the presence of non-aminoacylated tRNAs in the ribosome A site (52,53). Integrating this work with a model of growth rate control via ppGpp that was recently implemented in our lab (54) would give us the opportunity to explore overall growth responses in amino acid-limited media, building on work such as that of Elf *et al.* (6).

Finally, we recognize that the detailed and holistic investigations performed here were only made possible by embedding the tRNA aminoacylation system into a large-scale model that already accounts for millions of data points reported in thousands of studies by hundreds of labs over the past several decades (5). Taken together, the expansion to the *E. coli* model reported here advances the depth of mechanistic detail incorporated into the translational machinery and enhances the breadth of potential for generating predictions and accelerating biological discovery. We anticipate that as other functionalities are incorporated into the *E. coli* Whole Cell Modeling Project (8), similarly remarkable and unexpected phenotypes will continue to emerge and catalyze the accompanying biochemical and/or cell biological measurements that lead to new discoveries.

## DATA AVAILABILITY

The code and data used in this study have been archived to Zenodo with the permanent Digital Object Identifier (DOI) 10.5281/zenodo.7859480. Additionally, the E. coli Whole Cell Modeling Project is an open source collaborative initiative available in the GitHub repository: https://github.com/CovertLab/WholeCellEcoliRelease/releases/tag/v3.0.1.

## Supplementary Material

gkad435_Supplemental_File
