# Supplementary material for: Whole-cell modeling of E. coli confirms that in vitro tRNA aminoacylation measurements are insufficient to support cell growth and predicts a positive feedback mechanism regulating arginine biosynthesis
Source: Nucleic Acids Res. 2023 May 24;51(12):5911–30. doi: 10.1093/nar/gkad435 (PMC10325894; doi:10.1093/nar/gkad435)
Supplement: gkad435_Supplemental_File [file gkad435_supplemental_file.pdf]

Supporting Material to Whole-cell modeling of E. coli confirms that in vitro tRNA aminoacylation measurements are insufficient to support cell growth and predicts a positive feedback mechanism regulating arginine biosynthesis

Heejo Choi, Markus Covert

This document serves as Supporting Material to **Whole-cell modeling of E. coli confirms that in vitro tRNA aminoacylation measurements are insufficient to support cell growth and predicts a positive feedback mechanism regulating arginine biosynthesis**. Here, we describe the methods of the study in four sections: Model Design (Section 1), its realization during Optimization of Aminoacyl-tRNA Synthetase Kinetic Parameters (Section 2) and during Simulation (Section 3), and Analysis (Section 4), which details how the work in the main study was performed. This study builds upon the E. coli Model published in 2020 [1], where details of the E. coli Model beyond the scope of this study are described.

Contents

1 Model Design **4**

1.1 tRNA Aminoacylation by Aminoacyl-tRNA Synthetases 5

1.1.1 Represented Molecules 6

1.2 Codon-to-tRNA Anticodon Interactions 7

1.2.1 Represented Codons 11

1.3 Utilization of Aminoacyl-tRNAs by Ribosomes 11

1.4 N-terminal Cleavage of Initial Methionines 12

2 Optimization of Aminoacyl-tRNA Synthetase Kinetic Parameters **19**

2.1 Approach 19

2.2 Objective Function 19

2.2.1 Steady-State Error 20

2.2.2 Regularization 27

2.2.3 Bounds Penalty 27

2.2.4 Complete Form 28

2.3 Generation of Solutions 29

2.3.1 Random Initialization 29

2.3.2 Parametric Sweep on the Minimum Aminoacyl-tRNA Synthetase Concentration . . . 30

2.3.3 Comprehensive Algorithm 32

3 Simulation **33**

3.1 Estimation of Ribosome Steps 33

3.2 Determination of Feasibility 33

3.3 Reconciliation of Kinetic and Sequence Solutions 35

3.3.1 Motivation 35

3.3.2 Tools 35

3.3.3 Strategy 37

3.4 Update of Molecules 40
3.5 N-terminal Cleavage of Initial Methionines 41

4 Analysis **42**
4.1 Simulations 42
4.2 Methods 43
4.2.1 Analysis Methods for Panels in Figure 2 43
4.2.2 Analysis Methods for Panels in Figure 3 44
4.2.3 Analysis Methods for Panels in Figure 4 46
4.2.4 Analysis Methods for Panels in Figure 5 47
4.2.5 Analysis Methods for Panels in Figure 6 49
50subsubsection.4.2.6
50subsubsection.4.2.7
50subsubsection.4.2.8
51subsubsection.4.2.9
51subsubsection.4.2.10**5 Supporting Figures and Tables** **53**

1 Model Design

The cycle of tRNA usage – aminoacylation by aminoacyl-tRNA synthetases and tRNA release after amino acid transfer by elongating ribosomes – is a system that consists of both deterministic and stochastic features.

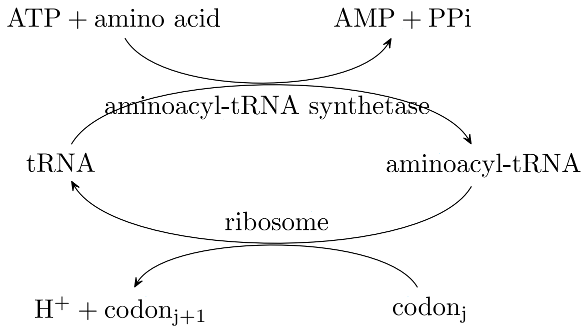

Figure 1: **The tRNA aminoacylation and cycle.** Unaminoacylated tRNAs are aminoacylated by aminoacyl-tRNA synthetases. As aminoacyl-tRNAs are used by ribosomes during translation elongation, tRNAs are returned to their unaminoacylated forms.

While tRNA aminoacylation can be modeled deterministically as mass action kinetics with ordinary differential equations (ODEs), the ribosome's utilization of aminoacyl-tRNAs for synthesizing polypeptides depends on the identity of the codon at the ribosome A site, which necessarily depends on the mRNA sequence and may be more appropriately represented by a stochastic model such as the Gillespie algorithm.

For example, aminoacyl-tRNA utilization by ribosomes can be represented as the following one-step reaction:

$$\text{aminoacyl-tRNA}_i + \text{ribosome}_j \rightarrow \text{tRNA}_i + \text{ribosome}_{j+1} + H^+ \tag{1}$$

where aminoacyl-tRNA$_i$ is the aminoacylated form of the i-th tRNA isoacceptor and ribosome$_j$ is an active ribosome with an open A site at the j-th codon on the mRNA transcript. Notably, i indicates the identity of the tRNA isoacceptor whereas j indicates a position along the mRNA transcript. Consequently, as the ribosome processes along the mRNA, the identity of the codon at the j-th position changes and the tRNA isoacceptors that are eligible to interact with the j-th codon also change.

In addition to the tRNA aminoacylation cycle, the whole-cell modeling framework also consists of qualities that lends itself well to both deterministic and stochastic modeling approaches. On one hand, the simulation time step (which is on the order of seconds) enables the translation process to read 2 to 5×10^5 codons per second by 1 to 3×10^4 active ribosomes, each processing at approximately 17.5 amino acids per second – a fast reaction rate that can be appropriately modeled deterministically as bulk changes. On the other hand, the positions of active ribosomes on mRNA transcripts are detailed to the codon residing at the A site (previously detailed to the residue of the nascent polypeptide), and the sequence order of codons on mRNAs is obeyed – meaning that ribosomes cannot progress to the next codon until the current one has been successfully read. This codon-by-codon procession of ribosomes along mRNAs, which depends on the identity of the j-th codon and the availability of the i-th aminoacyl-tRNA, can be suitably modeled by a stochastic simulation approach.

Thus, to be compatible with both the deterministic and stochastic qualities of the tRNA aminoacylation cycle and the whole-cell modeling framework, the following three-step strategy was developed for use during simulations:

1. Calculate the kinetic limitations of aminoacyl-tRNA synthetases, assuming a constant codon reading rate, during the time Δt:
   (a) Estimate the codon reading rate as a bulk change during Δt, based on the upcoming codon sequences of mRNAs being translated by active ribosomes and the anticipated ribosome elongation rate.
   (b) Simulate the tRNA aminoacylation cycle as a deterministic ODE model during time Δt, where tRNA aminoacylation and aminoacyl-tRNA utilization by ribosomes (estimated as the codon reading rate in step 1(a)) are modeled by mass action kinetics.
   (c) Retrieve the number of times each codon type was read during Δt from the ODE solution.
2. Process ribosomes along mRNAs according to the kinetic limitations and the sequence order:
   (a) Process ribosomes codon-by-codon – so as to obey the sequence order of codons on mRNAs – as far as possible without exceeding the number of codons read in step 1(c).
   (b) Retrieve the number of times each codon type was read after all ribosomes are processed as far as possible.
3. Reconcile disagreements, if any, between the number of codons read in step 1(c) and step 2(b):
   (a) Perform a random search for the optimal combination of ribosome positions along mRNAs that minimize disagreements while obeying the kinetic limitation determined in step 1(c).
   (b) Any disagreements that remain by this point are kinetic over-estimations (where the ODE model estimated more codon reading events than the sequence order permitted); reconcile these disagreements by undo-ing the appropriate number of codon reading events in the ODE solution (ie. returning an unaminoacylated tRNA to its aminoacylated form), which may require undo-ing the number of aminoacylation events (ie. returning an aminoacyl-tRNA to its unaminoacylated form) if the pool of aminoacyl-tRNAs is too small.

The result of these steps is a representation of tRNA aminoacylation cycles and codon-by-codon ribosome procession that obeys both the kinetic constraints of aminoacyl-tRNA synthetases and the sequence order of codons encountered by ribosomes along mRNAs.

1.1 tRNA Aminoacylation by Aminoacyl-tRNA Synthetases

Aminoacylation of each tRNA isoacceptor was represented as a one-step reaction:

$$\text{tRNA} + \text{amino acid} + \text{ATP} \rightarrow \text{aminoacyl-tRNA} + \text{AMP} + \text{PPi} \tag{2}$$

By assuming 1) ATP saturation (a high affinity between the aminoacyl-tRNA synthetase and ATP, such that ATP binding is not limiting), 2) random-ordered substrate-binding, and 3) competition between tRNA isoacceptors that share the same aminoacyl-tRNA synthetase, the following reaction scheme can be described for an aminoacyl-tRNA synthetase with two competing tRNA isoacceptors:

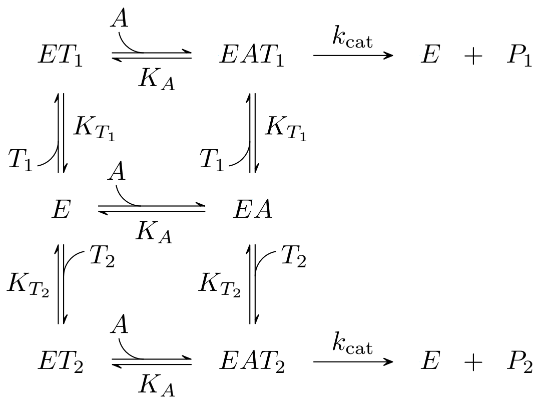

Figure 2: **tRNA aminoacylation described using Michaelis-Menten enzyme kinetics.** tRNA aminoacylation reaction scheme assumes ATP saturation, random-ordered substrate-binding, and competition between two tRNA isoacceptors sharing the same aminoacyl-tRNA synthetase.

where E is the aminoacyl-tRNA synthetase, A is the amino acid, T is the unaminoacylated tRNA, and P is the aminoacyl-tRNA. The Michaelis-Menten constants K_A and K_T describe the affinity between the aminoacyl-tRNA synthetase and its substrates – amino acid and tRNA, respectively.

According to Michaelis-Menten enzyme kinetics, the rate of formation of the i-th aminoacyl-tRNA can be described as:

$$v_{aminoacylation,i} = \frac{dP_i}{dt} = k_{cat}[E]\frac{[A]}{K_A + [A]}\frac{\frac{[T_i]}{K_{T,i}}}{1 + \frac{[T_1]}{K_{T,1}} + \frac{[T_2]}{K_{T,2}}} \quad (3)$$

where k_{cat} is the rate of catalysis of the aminoacyl-tRNA synthetase.

Convenience kinetics [2] can be used to generalize the rate of aminoacyl-tRNA formation for aminoacyl-tRNA synthetases with n tRNA isoacceptors:

$$v_{aminoacylation,i} = k_{cat}[E]\frac{[A]}{K_A + [A]}\frac{\frac{[T_i]}{K_{T,i}}}{1 + \sum_{j=1}^{n}\frac{[T_j]}{K_{T,j}}} \quad (4)$$

1.1.1 Represented Molecules

E. coli K-12 MG1655 has 23 documented aminoacyl-tRNA synthetase genes [3], of which 22 are modeled and 1 (*lysU*) is excluded. Lysine tRNAs are reported to be aminoacylated by two aminoacyl-tRNA synthetases: *lysS* – which is modeled – and *lysU*, which is expressed at elevated temperatures and was thereby excluded from our model of cell growth at 37°C.

Aminoacyl-tRNA Synthetase	Gene(s)	Subunit Stoichiometry
AlaRS	alaS	2
ArgRS	argS	1
AsnRS	asnS	2
AspRS	aspS	2
CysRS	cysS	1
GluRS	gltX	1
GlnRS	glnS	1
GlyRS	glyS	2
	glyQ	2
HisRS	hisS	2
IleRS	ileS	1
LeuRS	leuS	1
LysRS	lysS	2
MetRS	metG	2
PheRS	pheS	2
	pheT	2
ProRS	proS	2
SerRS	serS	2
ThrRS	thrS	2
TrpRS	trpS	2
TyrRS	tyrS	2
ValRS	valS	1

Table 1: Aminoacyl-tRNA synthetases represented in this study. Data were retrieved from EcoCyc [3].

Of the 86 documented tRNAs [3], 85 are mechanistically modeled in this study and 1 (*selC*, the sole tRNA for Selenocysteine) is assumed to be not limiting, as was done in the prior E. coli Model [1].

Amino Acid	tRNA Genes
Alanine	alaT
	alaU
	alaV
	alaW
	alaX
Arginine	argQ
	argU
	argV
	argW
	argX
	argY
	argZ
Asparagine	asnT
	asnU
	asnV
	asnW
Aspartate	aspT
	aspU
	aspV
Cysteine	cysT
Glutamine	glnU
	glnV
	glnW
	glnX
Glutamate	gltT
	gltU
	gltV
	gltW
Glycine	glyT
	glyU
	glyV
	glyW
	glyX
	glyY
Histidine	hisR
Isoleucine	ileT
	ileU
	ileV
	ileX
	ileY
Leucine	leuP
	leuQ
	leuT
	leuU
	leuV
	leuW
	leuX
	leuZ
Lysine	lysQ
	lysT
	lysV
	lysW
	lysY
	lysZ
Methionine	metT
	metU
	metV
	metW
	metY
	metZ
Phenylalanine	pheU
	pheV
Proline	proK
	proL
	proM
Serine	serT
	serU
	serV
	serW
	serX
Threonine	thrT
	thrU
	thrV
	thrW
Tryptophan	trpT
Tyrosine	tyrT
	tyrU
	tyrV
Valine	valT
	valU
	valV
	valW
	valX
	valY
	valZ

Table 2: tRNAs represented in this study. Data were retrieved from EcoCyc [3].

1.2 Codon-to-tRNA Anticodon Interactions

The interactions of mRNA codons to tRNA anticodons were described by:

1. Watson-Crick Base-Pairing Rules
   - Adenine (A) with Uracil (U)

- Guanine (G) with Cytosine (C)

2. Wobble Hypothesis [4]
   - Guanine (G) with Uracil (U)
   - Wobble base-pairing only occurs in the wobble position: the tRNA 5' anticodon base with the mRNA 3' codon base.

3. Initiator and Elongator Methionine tRNAs
   - *E. coli* has two types of methionine tRNAs: the initiator methionyl-tRNA used for translation initiation and the elongator methionyl-tRNA used for translation elongation. Initiator methionyl-tRNAs are formylated by Fmt, and are thereby distinguished from elongator methionyl-tRNAs by the cell's translation machinery [3].
   - The four reported initiator methionine tRNAs are: metV, metW, metY, and metZ; the two reported elongator methionine tRNAs are: metT and metU [3].

4. Experimental Evidence
   - Arginine codons 5'-CGA-3' and 5'-CGC-3' were reported to be decoded by the tRNA isoacceptors argQ, argV, argY, and argZ, which contain the anticodon 3'-GCI-5', where I is inosine, an adenosine derivative [5, 6].
   - Isoleucine codon 5'-AUA-3' was reported to be decoded by the tRNA isoacceptors ileX and ileY, which contain the anticodon 3'-UAL-5', where L is lysidine, a cytidine derivative [7, 8].

To enable matrix operations, the codon-to-tRNA anticodon interactions for each amino acid family have been summarized into the following Boolean matrices:

$$\boldsymbol{M}_{\text{Alanine}} = \begin{array}{r} \\ alaT(UGC) \\ alaU(UGC) \\ alaV(UGC) \\ alaW(GGC) \\ alaX(GGC) \end{array} \begin{array}{c} \begin{array}{cccc} GCA & GCC & GCG & GCU \end{array} \\ \begin{bmatrix} 1 & 0 & 1 & 0 \\ 1 & 0 & 1 & 0 \\ 1 & 0 & 1 & 0 \\ 0 & 1 & 0 & 1 \\ 0 & 1 & 0 & 1 \end{bmatrix} \end{array} \tag{5}$$

$$\boldsymbol{M}_{\text{Arginine}} = \begin{array}{r} \\ argQ(ICG) \\ argU(UCU) \\ argV(ICG) \\ argW(CCU) \\ argX(CCG) \\ argY(ICG) \\ argZ(ICG) \end{array} \begin{array}{c} \begin{array}{cccccc} AGA & AGG & CGA & CGC & CGG & CGU \end{array} \\ \begin{bmatrix} 0 & 0 & 1 & 1 & 0 & 1 \\ 1 & 1 & 0 & 0 & 0 & 0 \\ 0 & 0 & 1 & 1 & 0 & 1 \\ 0 & 1 & 0 & 0 & 0 & 0 \\ 0 & 0 & 0 & 0 & 1 & 0 \\ 0 & 0 & 1 & 1 & 0 & 1 \\ 0 & 0 & 1 & 1 & 0 & 1 \end{bmatrix} \end{array} \tag{6}$$

$$\boldsymbol{M}_{\text{Asparagine}} = \begin{array}{r} \\ asnT(GUU) \\ asnU(GUU) \\ asnV(GUU) \\ asnW(GUU) \end{array} \begin{array}{c} \begin{array}{cc} AAC & AAU \end{array} \\ \begin{bmatrix} 1 & 1 \\ 1 & 1 \\ 1 & 1 \\ 1 & 1 \end{bmatrix} \end{array} \tag{7}$$

$$\boldsymbol{M}_{\text{Aspartate}} = \begin{array}{r} \\ aspT(GUC) \\ aspU(GUC) \\ aspV(GUC) \end{array} \begin{array}{c} \begin{array}{cc} GAC & GAU \end{array} \\ \begin{bmatrix} 1 & 1 \\ 1 & 1 \\ 1 & 1 \end{bmatrix} \end{array} \tag{8}$$

$$\boldsymbol{M}_{\text{Cysteine}} = \begin{array}{c} \\ cysT(GCA) \end{array} \begin{array}{c} \begin{array}{cc} UGC & UGU \end{array} \\ \begin{bmatrix} 1 & 1 \end{bmatrix} \end{array} \tag{9}$$

$$\boldsymbol{M}_{\text{Glutamine}} = \begin{array}{r} \\ glnU(UUG) \\ glnV(CUG) \\ glnW(UUG) \\ glnX(CUG) \end{array} \begin{array}{c} \begin{array}{cc} CAA & CAG \end{array} \\ \begin{bmatrix} 1 & 1 \\ 0 & 1 \\ 1 & 1 \\ 0 & 1 \end{bmatrix} \end{array} \tag{10}$$

$$\boldsymbol{M}_{\text{Glutamate}} = \begin{array}{r} \\ gltT(UUC) \\ gltU(UUC) \\ gltV(UUC) \\ gltW(UUC) \end{array} \begin{array}{c} \begin{array}{cc} GAA & GAG \end{array} \\ \begin{bmatrix} 1 & 1 \\ 1 & 1 \\ 1 & 1 \\ 1 & 1 \end{bmatrix} \end{array} \tag{11}$$

$$\boldsymbol{M}_{\text{Glycine}} = \begin{array}{r} \\ glyT(UCC) \\ glyU(CCC) \\ glyV(GCC) \\ glyW(GCC) \\ glyX(GCC) \\ glyY(GCC) \end{array} \begin{array}{c} \begin{array}{cccc} GGA & GGC & GGG & GGU \end{array} \\ \begin{bmatrix} 1 & 0 & 1 & 0 \\ 0 & 0 & 1 & 0 \\ 0 & 1 & 0 & 1 \\ 0 & 1 & 0 & 1 \\ 0 & 1 & 0 & 1 \\ 0 & 1 & 0 & 1 \end{bmatrix} \end{array} \tag{12}$$

$$\boldsymbol{M}_{\text{Histidine}} = \begin{array}{c} \\ hisR(GUG) \end{array} \begin{array}{c} \begin{array}{cc} CAC & CAU \end{array} \\ \begin{bmatrix} 1 & 1 \end{bmatrix} \end{array} \tag{13}$$

$$\boldsymbol{M}_{\text{Isoleucine}} = \begin{array}{r} \\ ileT(GAU) \\ ileU(GAU) \\ ileV(GAU) \\ ileX(LAU) \\ ileY(LAU) \end{array} \begin{array}{c} \begin{array}{ccc} AUA & AUC & AUU \end{array} \\ \begin{bmatrix} 0 & 1 & 1 \\ 0 & 1 & 1 \\ 0 & 1 & 1 \\ 1 & 0 & 0 \\ 1 & 0 & 0 \end{bmatrix} \end{array} \tag{14}$$

$$\boldsymbol{M}_{\text{Leucine}} = \begin{array}{r} \\ leuP(CAG) \\ leuQ(CAG) \\ leuT(CAG) \\ leuU(GAG) \\ leuV(CAG) \\ leuW(UAG) \\ leuX(CAA) \\ leuZ(UAA) \end{array} \begin{array}{c} \begin{array}{cccccc} CUA & CUC & CUG & CUU & UUA & UUG \end{array} \\ \begin{bmatrix} 0 & 0 & 1 & 0 & 0 & 0 \\ 0 & 0 & 1 & 0 & 0 & 0 \\ 0 & 0 & 1 & 0 & 0 & 0 \\ 0 & 1 & 0 & 1 & 0 & 0 \\ 0 & 0 & 1 & 0 & 0 & 0 \\ 1 & 0 & 1 & 0 & 0 & 0 \\ 0 & 0 & 0 & 0 & 0 & 1 \\ 0 & 0 & 0 & 0 & 1 & 1 \end{bmatrix} \end{array} \tag{15}$$

$$\boldsymbol{M}_{\text{Lysine}} = \begin{array}{r} \\ lysQ(UUU) \\ lysT(UUU) \\ lysV(UUU) \\ lysW(UUU) \\ lysY(UUU) \\ lysZ(UUU) \end{array} \begin{array}{c} \begin{array}{cc} AAA & AAG \end{array} \\ \begin{bmatrix} 1 & 1 \\ 1 & 1 \\ 1 & 1 \\ 1 & 1 \\ 1 & 1 \\ 1 & 1 \end{bmatrix} \end{array} \tag{16}$$

$$\boldsymbol{M}_{\text{Methionine}} = \begin{array}{r} \\ metT(CAU) \\ metU(CAU) \\ metV(CAU) \\ metW(CAU) \\ metY(CAU) \\ metZ(CAU) \end{array} \begin{array}{c} \begin{array}{cc} start & AUG \end{array} \\ \begin{bmatrix} 0 & 1 \\ 0 & 1 \\ 1 & 0 \\ 1 & 0 \\ 1 & 0 \\ 1 & 0 \end{bmatrix} \end{array} \tag{17}$$

$$\boldsymbol{M}_{\text{Phenylalanine}} = \begin{array}{r} \\ pheU(GAA) \\ pheV(GAA) \end{array} \begin{array}{c} \begin{array}{cc} UUC & UUU \end{array} \\ \begin{bmatrix} 1 & 1 \\ 1 & 1 \end{bmatrix} \end{array} \tag{18}$$

$$\boldsymbol{M}_{\text{Proline}} = \begin{array}{r} \\ proK(CGG) \\ proL(GGG) \\ proM(UGG) \end{array} \begin{array}{c} \begin{array}{cccc} CCA & CCC & CCG & CCU \end{array} \\ \begin{bmatrix} 0 & 0 & 1 & 0 \\ 0 & 1 & 0 & 1 \\ 1 & 0 & 1 & 0 \end{bmatrix} \end{array} \tag{19}$$

$$\boldsymbol{M}_{\text{Serine}} = \begin{array}{r} \\ serT(UGA) \\ serU(CGA) \\ serV(GCU) \\ serW(GGA) \\ serX(GGA) \end{array} \begin{array}{c} \begin{array}{cccccc} AGC & AGU & UCA & UCC & UCG & UCU \end{array} \\ \begin{bmatrix} 0 & 0 & 1 & 0 & 1 & 0 \\ 0 & 0 & 0 & 0 & 1 & 0 \\ 1 & 1 & 0 & 0 & 0 & 0 \\ 0 & 0 & 0 & 1 & 0 & 1 \\ 0 & 0 & 0 & 1 & 0 & 1 \end{bmatrix} \end{array} \tag{20}$$

$$\boldsymbol{M}_{\text{Threonine}} = \begin{array}{r} \\ thrT(GGU) \\ thrU(UGU) \\ thrV(GGU) \\ thrW(CGU) \end{array} \begin{array}{c} \begin{array}{cccc} ACA & ACC & ACG & ACU \end{array} \\ \begin{bmatrix} 0 & 1 & 0 & 1 \\ 1 & 0 & 1 & 0 \\ 0 & 1 & 0 & 1 \\ 0 & 0 & 1 & 0 \end{bmatrix} \end{array} \tag{21}$$

$$\boldsymbol{M}_{\text{Tryptophan}} = trpT(CCA) \begin{array}{c} UGG \\ \begin{bmatrix} 1 \end{bmatrix} \end{array} \tag{22}$$

$$\boldsymbol{M}_{\text{Tyrosine}} = \begin{array}{r} \\ tyrT(GUA) \\ tyrU(GUA) \\ tyrV(GUA) \end{array} \begin{array}{c} \begin{array}{cc} UAC & UAU \end{array} \\ \begin{bmatrix} 1 & 1 \\ 1 & 1 \\ 1 & 1 \end{bmatrix} \end{array} \tag{23}$$

$$\boldsymbol{M}_{\text{Valine}} = \begin{array}{r} \\ valT(UAC) \\ valU(UAC) \\ valV(GAC) \\ valW(GAC) \\ valX(UAC) \\ valY(UAC) \\ valZ(UAC) \end{array} \begin{array}{c} \begin{array}{cccc} GUA & GUC & GUG & GUU \end{array} \\ \begin{bmatrix} 1 & 0 & 1 & 0 \\ 1 & 0 & 1 & 0 \\ 0 & 1 & 0 & 1 \\ 0 & 1 & 0 & 1 \\ 1 & 0 & 1 & 0 \\ 1 & 0 & 1 & 0 \\ 1 & 0 & 1 & 0 \end{bmatrix} \end{array} \tag{24}$$

1.2.1 Represented Codons

Of the 64 possible codons, all 61 sense codons were mechanistically modeled and the role of the 3 stop codons were assumed by instantaneously disassembling active ribosomes and releasing the finished protein product after the final sense codon is processed.

Additionally, start codons were distinguished from elongating AUG codons in order to assign initiator and elongator Methionine tRNAs to their corresponding codons.

1.3 Utilization of Aminoacyl-tRNAs by Ribosomes

The utilization of aminoacyl-tRNAs by ribosomes (representing the release of tRNAs after amino acid transfer by elongating ribosomes) was represented as a one-step reaction:

$$\text{aminoacyl-tRNA}_i + \text{ribosome}_j \rightarrow \text{tRNA}_i + \text{ribosome}_{j+1} + H^+ \tag{25}$$

where aminoacyl-tRNA$_i$ is the aminoacylated form of the i-th tRNA isoacceptor, and ribosome$_j$ is an active ribosome with an open A site at the j-th codon on the mRNA transcript.

For codons that can be read by multiple aminoacyl-tRNA isoacceptors, the distribution of codon reading load across the different aminoacyl-tRNA isoacceptors was assumed to match the relative abundances of those aminoacyl-tRNAs. To do so, an $(n \times m)$ matrix $\boldsymbol{T}$ was described for each amino acid family:

$$\boldsymbol{T} = \begin{bmatrix} T_1^* & T_1^* & — & T_1^* \\ T_2^* & T_2^* & — & T_2^* \\ \vdots & \vdots & & \vdots \\ T_n^* & T_n^* & — & T_n^* \end{bmatrix} \tag{26}$$

where n is the number of tRNA isoacceptors, m is the number of codons, and T_i^* is the abundance of the i-th aminoacyl-tRNA isoacceptor in the amino acid family.

Then, the aminoacyl-tRNA abundances were masked by codon-to-tRNA anticodon interactions via element-wise multiplication:

$$\boldsymbol{D} = \boldsymbol{M} \otimes \boldsymbol{T} \tag{27}$$

where $\boldsymbol{M}$ is one of the matrices described by Equations 5 through 24 (depending on the amino acid family).

Finally, the columns of $\boldsymbol{D}$ were normalized to yield the codon reading load distribution $\bar{\boldsymbol{D}}$, which has elements:

$$\bar{\boldsymbol{D}}_{\boldsymbol{ij}} = \frac{\boldsymbol{D}_{\boldsymbol{ij}}}{\sum_{i=1}^{n} \boldsymbol{D}_{\boldsymbol{ij}}} \tag{28}$$

During optimization of aminoacyl-tRNA synthetase kinetic parameters (Section 2) and simulation (Section 3.1), a vector describing the codon reading rate, v_{codon} is prepared. The dot product of $\bar{\boldsymbol{D}}$ and v_{codon} yields the utilization rate of each aminoacyl-tRNA isoacceptor by active ribosomes.

$$v_{utilization} = \bar{\boldsymbol{D}} \cdot v_{codon} \tag{29}$$

1.4 N-terminal Cleavage of Initial Methionines

The removal of the N-terminal methionine residue of nascent polypeptides by methionine aminopeptidase (MAP) was modeled by Michaelis-Menten enzyme kinetics assuming maximal reaction rates:

$$v_{MAP,max} = k_{cat}[MAP] \tag{30}$$

where k_{cat} is the rate of catalysis and takes the value 6 s^{-1} [9], and [E] is the cellular concentration of MAP molecules. Table 3 lists the 385 documented substrates of MAP retrieved from EcoCyc [3], and Section 3.5 and Algorithm 5 detail the model of N-terminal cleavage of initial methionines by MAP during simulation.

Protein	Gene
acetyl-CoA carboxyltransferase subunit α	accA
isocitrate lyase	aceA
pyruvate dehydrogenase E1 component	aceE
pyruvate dehydrogenase, E2 subunit	aceF
isocitrate dehydrogenase kinase/phosphatase	aceK
aconitate hydratase 1	acnA
acyl carrier protein	acpP
holo-[acyl-carrier-protein] synthase	acpS
fused acetaldehyde-CoA dehydrogenase and iron-dependent alcohol dehydrogenasealdehyde/alcohol dehydrogenase AdhE	adhE
alkyl hydroperoxide reductase, AhpC component	ahpC
alanine—tRNA ligase/DNA-binding transcriptional repressor	alaS
aldehyde dehydrogenase A	aldA
ribulokinase	araB
acetylglutamate kinase	argB
N-acetylornithine aminotransferase / N-succinyldiaminopimelate aminotransferase	argD
ornithine carbamoyltransferase	argF
argininosuccinate synthetase	argG
ornithine carbamoyltransferase	argI
chorismate synthase	aroC
shikimate kinase 1	aroK
shikimate kinase 2	aroL
asparagine synthetase B	asnB
asparagine—tRNA ligase	asnS
ATP synthase F_1 complex subunit ε	atpC

Table 3

Protein	Gene
ATP synthase F_1 complex subunit β	atpD
ATP synthase accessory factor AtpI	atpI
FMN dependent NADH:quinone oxidoreductase	azoR
betaine aldehyde dehydrogenase	betB
dethiobiotin synthetase	bioD
8-amino-7-oxononanoate synthase	bioF
crotonobetainyl-CoA hydratase	caiD
carbamoyl-phosphate synthetase large subunit	carB
type I-E CRISPR system Cascade subunit CasC	casC
pre-CRISPR RNA endonuclease	casE
cyclopropane fatty acyl phospholipid synthase	cfa
monoacetylchitobiose-6-phosphate hydrolase	chbF
chemotaxis protein CheY	cheY
ClpX ATP-dependent protease specificity component and chaperone	clpX
cytidylate kinase	cmk
cytosine/isoguanine deaminase	codA
RNA polymerase holoenzyme assembly factor Crl	crl
DNA-binding transcriptional dual regulator CRP	crp
Enzyme IIA^{Glc}	crr
cold shock protein CspA	cspA
transcription antiterminator and regulator of mRNA stability CspC	cspC
transcription antiterminator and regulator of RNA stability CspE	cspE
adenylyl-sulfate kinase	cysC
phosphoadenosine phosphosulfate reductase	cysH
sulfite reductase, hemoprotein subunit	cysI
sulfite reductase, flavoprotein subunit complex	cysJ
cysteine synthase A	cysK
peptidyl-dipeptidase Dcp	dcp
D-cysteine desulfhydrase, PLP-dependent / 3-chloro-D-alanine dehydrochlorinase	dcyD
D-alanine—D-alanine ligase B	ddlB
ATP-dependent RNA helicase DeaD	deaD
peptide deformylase	def
phosphopentomutase	deoB
deoxyribose-phosphate aldolase	deoC
purine nucleoside phosphorylase	deoD
fused 4'-phosphopantothenoylcysteine decarboxylase and phosphopantothenoylcysteine synthetase	dfp
diacylglycerol kinase	dgkA
dGTP triphosphohydrolase	dgt
quinone-dependent D-lactate dehydrogenase	dld
dimethyl sulfoxide reductase subunit B	dmsB
redox enzyme maturation protein DmsD	dmsD
chaperone protein DnaJ	dnaJ
chaperone protein DnaK	dnaK
primosomal protein DnaT	dnaT
stationary phase nucleoid component that sequesters iron and protects DNA from damage	dps
1-deoxy-D-xylulose-5-phosphate synthase	dxs
protein chain elongation factor EF-P	efp
enolase	eno
enterobactin synthase component B	entB
2,3-dihydroxybenzoate-[aryl-carrier protein] ligase	entE
D-erythrose-4-phosphate dehydrogenase	epd
30S ribosomal subunit maturation GTPase Era	era
energy-dependent translational throttle protein EttA	ettA
β-hydroxyacyl-acyl carrier protein dehydratase/isomerase	fabA
[acyl-carrier-protein] *S*-malonyltransferase	fabD
3-oxoacyl-[acyl carrier protein] synthase 2	fabF
enoyl-[acyl-carrier-protein] reductase	fabI
2,4-dienoyl-CoA reductase	fadH
DNA-binding transcriptional dual regulator FadR	fadR
fructose-bisphosphate aldolase class II	fbaA
fructose-bisphosphate aldolase class I	fbaB
reduced ferredoxin	fdx
RNA polymerase sigma factor FecI	fecI
peptidyl-prolyl *cis-trans* isomerase FklB	fklB
peptidyl-prolyl *cis-trans* isomerase FkpB	fkpB

Table 3

Protein	Gene
flavodoxin 1	fldA
flagellar hook protein FlgE	flgE
flagellar hook-filament junction protein 1	flgK
flagellar filament structural protein	fliC
flagellar filament capping protein	fliD
flagellar protein FliE	fliE
10-formyltetrahydrofolate:L-methionyl-tRNA^{fMet} N-formyltransferase	fmt
bifunctional methylenetetrahydrofolate dehydrogenase / methenyltetrahydrofolate cyclohydrolase	folD
GTP cyclohydrolase 1	folE
2-amino-4-hydroxy-6-hydroxymethyldihydropteridine diphosphokinase	folK
dihydroneopterin triphosphate 2'-epimerase	folX
flavodoxin/ferredoxin-NADP$^+$ reductase	fpr
fumarate reductase flavoprotein subunit	frdA
fumarate reductase iron-sulfur protein	frdB
NAD(P)H-flavin reductase	fre
signal recognition particle receptor	ftsY
L-fucose:H+ symporter	fucP
fumarase A	fumA
DNA-binding transcriptional dual regulator Fur	fur
elongation factor G	fusA
galactokinase	galK
galactose-1-phosphate uridylyltransferase	galT
UTP—glucose-1-phosphate uridylyltransferase	galU
glyceraldehyde-3-phosphate dehydrogenase	gapA
glyoxylate carboligase	gcl
glycine cleavage system H protein	gcvH
glycine decarboxylase	gcvP
putative transcriptional regulator GcvR	gcvR
aminomethyltransferase	gcvT
Protein	Gene
malate synthase G	glcB
glucose-1-phosphate adenylyltransferase	glgC
phosphoglucosamine mutase	glmM
L-glutamine—D-fructose-6-phosphate aminotransferase	glmS
glutamine synthetase	glnA
glutamine—tRNA ligase	glnS
glycerol kinase	glpK
glutamate synthase subunit GltD	gltD
glycine—tRNA ligase subunit β	glyS
D-*glycero*-β-D-*manno*-heptose-1,7-bisphosphate 7-phosphatase	gmhB
D-gluconate kinase, thermostable	gntK
2,3-bisphosphoglycerate-dependent phosphoglycerate mutase	gpmA
xanthine-guanine phsophoribosyltransferase	gpt
chaperonin GroEL	groL
nucleotide exchange factor GrpE	grpE
reduced glutaredoxin 3	grxC
glutaredoxin 4	grxD
fused glutathionylspermidine amidase / glutathionylspermidine synthetase	gss
D-glucarate dehydratase	gudD
D-arabinose 5-phosphate isomerase GutQ	gutQ
DNA gyrase subunit A	gyrA
DNA gyrase subunit B	gyrB
protein/nucleic acid deglycase 1	hchA
porphobilinogen synthase	hemB
RNA-binding protein Hfq	hfq
histidinol dehydrogenase	hisD
histidine—tRNA ligase	hisS
hemolysin E	hlyE
DNA-binding transcriptional dual regulator H-NS	hns
iron-sulfur cluster biosynthesis chaperone HscA	hscA
HslV hexamer	hslV
hydrogenase 2 large subunit	hybC
hydrogenase 3 maturation protease	hycI
hydrogenase maturation factor HypC	hypC
isoaspartyl dipeptidase proenzyme	iaaA

Table 3

Protein	Gene
integration host factor subunit α	ihfA
isoleucine—tRNA ligase	ileS
ketol-acid reductoisomerase ($NADP^+$)	ilvC
dihydroxy-acid dehydratase	ilvD
branched-chain-amino-acid aminotransferase	ilvE
translation initiation factor IF-1	infA
translation initiation factor IF-3	infC
scaffold protein for iron-sulfur cluster assembly	iscU
2-*C*-methyl-D-erythritol 4-phosphate cytidylyltransferase	ispD
K^+ binding protein	kbp
3-deoxy-*manno*-octulosonate cytidylyltransferase	kdsB
3-deoxy-D-*manno*-octulosonate 8-phosphate phosphatase KdsC	kdsC
galactoside *O*-acetyltransferase	lacA
β-galactosidase	lacZ
2-isopropylmalate synthase	leuA
3-isopropylmalate dehydrogenase	leuB
3-isopropylmalate dehydratase subunit LeuC	leuC
3-isopropylmalate dehydratase subunit LeuD	leuD
lipoprotein release complex - inner membrane subunit	lolE
Lon protease	lon
lipoamide dehydrogenase	lpd
lipoate—protein ligase A	lplA
lipopolysaccharide transport system ATP binding protein LptB	lptB
UDP-3-*O*-(3-hydroxymyristoyl)glucosamine *N*-acyltransferase	lpxD
DNA-binding transcriptional dual regulator Lrp	lrp
S-ribosylhomocysteine lyase	luxS
aspartate kinase III	lysC
lysine:H+ symporter	lysP
lysine—tRNA ligase, constitutive	lysS
lysine—tRNA ligase [multifunctional]	lysU
maltose *O*-acetyltransferase	maa
maltodextrin phosphorylase	malP
mannose-specific PTS enzyme IIAB component	manX
macrodomain Ori protein	maoP
methionine aminopeptidase	map
NADPH:quinone oxidoreductase MdaB	mdaB
melibiose:H^+/Na^+/Li^+ symporter	melB
homoserine *O*-succinyltransferase	metA
cystathionine β-lyase / L-cysteine desulfhydrase / alanine racemase	metC
cobalamin-independent homocysteine transmethylase	metE
methionine—tRNA ligase	metG
cobalamin-dependent methionine synthase	metH
DNA-binding transcriptional repressor MetJ	metJ
methionine adenosyltransferase	metK
fused aspartate kinase/homoserine dehydrogenase 2	metL
2-hydroxy-6-ketonona-2,4-dienedioate hydrolase	mhpC
Z-ring positioning protein MinD	minD
flavoprotein MioC	mioC
GTP 3',8'-cyclase	moaA
protein MoaB	moaB
cyclic pyranopterin monophosphate synthase	moaC
molybdopterin synthase catalytic subunit	moaE
molybdopterin-guanine dinucleotide biosynthesis adaptor protein	mobB
methionine sulfoxide reductase A	msrA
methionine sulfoxide reductase B	msrB
UDP-*N*-acetylmuramoyl-L-alanine—D-glutamate ligase	murD
UDP-*N*-acetylmuramoyl-L-alanyl-D-glutamate—2,6-diaminopimelate ligase	murE
N-acetylglucosaminyl transferase	murG
DNA mismatch repair protein MutH	mutH
DNA-formamidopyrimidine glycosylase	mutM
quinolinate phosphoribosyltransferase (decarboxylating)	nadC
N-acetylneuraminate lyase	nanA
nitrate reductase A subunit α	narG
nitrate reductase Z subunit α	narZ
NADH:quinone oxidoreductase II	ndh

Table 3

Protein	Gene
nucleoside diphosphate kinase	ndk
endonuclease VIII	nei
ribonucleoside-diphosphate reductase 1, β subunit dimer	nrdB
ribonucleoside-diphosphate reductase 2, α subunit dimer	nrdE
NADH:quinone oxidoreductase subunit CD	nuoC
NADH:quinone oxidoreductase subunit G	nuoG
transcription termination/antitermination factor NusG	nusG
oligoribonuclease	orn
osmotically inducible peroxiredoxin OsmC	osmC
trehalose-6-phosphate synthase	otsA
phenylacetyl-CoA thioesterase	paaI
protein-L-isoaspartate *O*-methyltransferase	pcm
pyridoxine 5'-phosphate oxidase / pyridoxamine 5'-phosphate oxidase	pdxH
pyridoxine 5'-phosphate synthase	pdxJ
peptidase D	pepD
aminopeptidase N	pepN
Xaa-Pro aminopeptidase	pepP
pyruvate formate-lyase activating enzyme	pflA
pyruvate formate-lyase (inactive)	pflB
phosphoglycerate kinase	pgk
phosphatidylglycerophosphatase B	pgpB
CDP-diacylglycerol—glycerol-3-phosphate 3-phosphatidyltransferase	pgsA
glycerol-3-phosphate 1-*O*-acyltransferase	plsB
nicotinate phosphoribosyltransferase	pncB
DNA polymerase II	polB
inorganic pyrophosphatase	ppa
peptidyl-prolyl *cis-trans* isomerase C	ppiC
polyphosphate kinase	ppk
phosphoenolpyruvate synthetase	ppsA
tautomerase PptA	pptA
exopolyphosphatase	ppx
peptide chain release factor RF3	prfC
primosomal replication protein N	priB
primosomal replication protein N''	priC
glutamate 5-kinase	proB
2-methylisocitrate lyase	prpB
2-methylcitrate dehydratase	prpD
ribose-phosphate diphosphokinase	prs
phage shock protein A	pspA
phosphate ABC transporter ATP binding subunit	pstB
phosphate acetyltransferase	pta
adenylosuccinate synthetase	purA
N^5-carboxyaminoimidazole ribonucleotide mutase	purE
amidophosphoribosyltransferase	purF
phosphoribosylformylglycinamide cyclo-ligase	purM
DNA-binding transcriptional repressor PurR	purR
phosphoribosylglycinamide formyltransferase 2	purT
fused DNA-binding transcriptional repressor / proline dehydrogenase / 1-pyrroline-5-carboxylate dehydrogenase PutA	putA
pyruvate kinase 2	pykA
aspartate carbamoyltransferase catalytic subunit	pyrB
dihydroorotase	pyrC
orotate phosphoribosyltransferase	pyrE
orotidine-5'-phosphate decarboxylase	pyrF
CTP synthetase	pyrG
UMP kinase	pyrH
aspartate carbamoyltransferase, regulatory subunit	pyrI
ribosome-associated inhibitor A	raiA
RNA polymerase-binding ATPase and RNAP recycling factor	rapA
30S ribosome binding factor	rbfA
DNA-binding transcriptional dual regulator RbsR	rbsR
DNA recombination/repair protein RecA	recA
exodeoxyribonuclease V subunit RecB	recB
recombination mediator protein RecF	recF
ATP-dependent DNA helicase RecQ	recQ

Table 3

Protein	Gene
ATP-dependent RNA helicase RhlB	rhlB
enamine/imine deaminase, redox-regulated chaperone	ridA
23S rRNA m^{5}U1939 methyltransferase	rlmD
23S rRNA pseudouridine^{746} and tRNA pseudouridine^{32} synthase	rluA
23S rRNA pseudouridine$^{1911/1915/1917}$ synthase	rluD
RNase G	rng
RNase R	rnr
50S ribosomal subunit protein L1	rplA
50S ribosomal subunit protein L2	rplB
50S ribosomal subunit protein L5	rplE
50S ribosomal subunit protein L6	rplF
50S ribosomal subunit protein L10	rplJ
50S ribosomal subunit protein L11	rplK
50S ribosomal subunit protein L7/L12 dimer	rplL
50S ribosomal subunit protein L19	rplS
50S ribosomal subunit protein L20	rplT
50S ribosomal subunit protein L24	rplX
50S ribosomal subunit protein L27	rpmA
50S ribosomal subunit protein L28	rpmB
50S ribosomal subunit protein L30	rpmD
50S ribosomal subunit protein L32	rpmF
50S ribosomal subunit protein L33	rpmG
50S ribosomal subunit protein L35	rpmI
RNA polymerase subunit ω	rpoZ
30S ribosomal subunit protein S2	rpsB
30S ribosomal subunit protein S3	rpsC
30S ribosomal subunit protein S4	rpsD
30S ribosomal subunit protein S5	rpsE
30S ribosomal subunit protein S7	rpsG
30S ribosomal subunit protein S8	rpsH
30S ribosomal subunit protein S9	rpsI
30S ribosomal subunit protein S11	rpsK
30S ribosomal subunit protein S12	rpsL
30S ribosomal subunit protein S13	rpsM
30S ribosomal subunit protein S14	rpsN
30S ribosomal subunit protein S15	rpsO
30S ribosomal subunit protein S17	rpsQ
30S ribosomal subunit protein S18	rpsR
30S ribosomal subunit protein S19	rpsS
30S ribosomal subunit protein S20	rpsT
30S ribosomal subunit protein S21	rpsU
16S rRNA m$^6_2$A1518,m$^6_2$A1519 dimethyltransferase	rsmA
16S rRNA m^{2}G1207 methyltransferase	rsmC
crossover junction endodeoxyribonuclease RuvC	ruvC
phosphoglycerate dehydrogenase	serA
phosphoserine/phosphohydroxythreonine aminotransferase	serC
SsrA-binding protein	smpB
superoxide dismutase (Mn)	sodA
superoxide dismutase (Fe)	sodB
DNA-binding transcriptional dual regulator SoxS	soxS
spermidine synthase	speE
spermidine N-acetyltransferase	speG
ssDNA-binding protein	ssb
3-mercaptopyruvate sulfurtransferase	sseA
stringent starvation protein A	sspA
serine/threonine:Na^+ symporter	sstT
$FMNH_2$-dependent alkanesulfonate monooxygenase	ssuD
soluble pyridine nucleotide transhydrogenase	sthA
dihydrolipoyltranssuccinylase	sucB
succinyl-CoA synthetase subunit α	sucD
Fe-S cluster scaffold complex subunit SufB	sufB
Fe-S cluster scaffold complex subunit SufD	sufD
transaldolase B	talB
trans-aconitate 2-methyltransferase	tam
twin arginine protein translocation system - TatC protein	tatC

Table 3

Protein	Gene
α-ketoglutarate-dependent taurine dioxygenase	tauD
acyl-CoA thioesterase II	tesB
RNA 2',3'-cyclic phosphodiesterase	thpR
transketolase 2	tktB
lipid hydroperoxide peroxidase	tpx
tryptophan synthase, β subunit dimer	trpB
anthranilate synthase subunit TrpD	trpD
DNA-binding transcriptional repressor TrpR	trpR
tRNA pseudouridine$^{38-40}$ synthase	truA
thioredoxin 1	trxA
thioredoxin reductase	trxB
protein chain elongation factor EF-Ts	tsf
translation elongation factor Tu 1	tufA
translation elongation factor Tu 2	tufB
tyrosine—tRNA ligase	tyrS
chorismate lyase	ubiC
uridine phosphorylase	udp
glycerophosphodiester phosphodiesterase UgpQ	ugpQ
uracil-DNA glycosylase	ung
universal stress protein A	uspA
universal stress protein E	uspE
UvrABC excision nuclease subunit B	uvrB
DNA mismatch endonuclease Vsr	vsr
NAD(P)H:quinone oxidoreductase	wrbA
exodeoxyribonuclease VII subunit XseB	xseB
putative glutamine amidotransferase YafJ	yafJ
nucleotide binding protein YajQ	yajQ
PF13993 family protein YccJ	yccJ
redox-responsive ATPase YchF	ychF
quinate/shikimate dehydrogenase	ydiB
genome maintenance protein	yedK
nucleoid-associated protein YejK	yejK
ferredoxin-like diferric-tyrosyl radical cofactor maintenance protein YfaE	yfaE
2,3-diaminopropionate ammonia-lyase	ygeX
folate-binding protein	ygfZ
putative Fe^{2+}-trafficking protein	yggX
protein/nucleic acid deglycase 2	yhbO
putative oxidoreductase YnfG	ynfG

Table 3: Substrates of methionine aminopeptidase (MAP). Data were retrieved from EcoCyc [3].

2 Optimization of Aminoacyl-tRNA Synthetase Kinetic Parameters

Parameter optimization of the aminoacyl-tRNA synthetase kinetic parameters was described as objective minimization problems, where each aminoacyl-tRNA synthetase was treated as an independent optimization problem.

The developments related to optimization of aminoacyl-tRNA synthetase kinetic parameters occurred in the following files of the E. coli Model repository:

wcEcoli Path	File
reconstruction/ecoli/	fit_sim_data_1.py
reconstruction/ecoli/dataclasses	relation.py
reconstruction/ecoli/flat	trna_charging_kinetics.tsv
reconstruction/ecoli/flat/optimization	trna_charging_kinetics_constants.tsv
reconstruction/ecoli/flat/optimization	trna_charging_kinetics_solutions.tsv
reconstruction/ecoli/flat/optimization	trna_synthetase_dynamic_range_sweep.tsv

Table 4: Table of files for optimization of aminoacyl-tRNA synthetase kinetic parameters.

The optimized aminoacyl-tRNA synthetase kinetic parameters are listed in Table S2.

2.1 Approach

For each aminoacyl-tRNA synthetase E, the rate of tRNA aminoacylation v of the i-th tRNA was described by Michaelis-Menten enzyme kinetics as described in Section 1.1, Equation 4.

$$v = k_{cat}[E]\frac{[A]}{K_A + [A]}\frac{\frac{f_i \cdot [T_i]}{K_{T,i}}}{1 + \sum_{j=1}^{n} \frac{f_j \cdot [T_j]}{K_{T,j}}} \tag{31}$$

where $[E]$ is the concentration of the aminoacyl-tRNA synthetase, $[A]$ is the concentration of the amino acid, $[T]$ is the concentration of total tRNA (ie. both aminoacylated and unaminoacylated forms), f is the fraction of $[T]$ that exists in the unaminoacylated form, K_A and K_T are the Michaelis-Menten constants describing the binding affinity between the aminoacyl-tRNA synthetase and its substrates – amino acid and tRNA – respectively, and n is the number of tRNA isoacceptors that aminoacyl-tRNA synthetase E interacts with.

During optimization, the free parameters that were solved for were: k_{cat}, K_A, K_T, and f. The constant parameters were: v, $[E]$, $[A]$, and $[T]$.

2.2 Objective Function

The objective function for each aminoacyl-tRNA synthetase problem takes the following form:

$$\text{minimize} \quad e_{ss} + (w_r \cdot R) + (w_b \cdot B) \tag{32}$$

where e_{ss} is the steady-state error, R is the regularization term, and B is the bounds penalty. The weights, w_r and w_b, describe the significance of R and B, respectively.

2.2.1 Steady-State Error

The steady-state error, e_{ss}, describes the relative error between the rates of tRNA aminoacylation v_A and aminoacyl-tRNA utilization by ribosomes v_D, which are anticipated to be equal at steady state according to the tRNA aminoacylation cycle depicted in Figure 1.

The rate of aminoacylation of the i-th tRNA isoacceptor is described by Equation 31 (reproduced below):

$$v_{A,i} = k_{cat}[E]\frac{[A]}{K_A + [A]}\frac{\frac{f_i \cdot [T_i]}{K_{T,i}}}{1 + \sum_{j=1}^{n} \frac{f_j \cdot [T_j]}{K_{T,j}}} \tag{33}$$

where the constants $[E]$, $[A]$, and $[T_i]$ were obtained from the result of the Parameter Calculator (Parca) in the E. coli Model using the Parca's estimate of the concentrations of aminoacyl-tRNA synthetases (Table 5), amino acids (Table 6), and each tRNA isoacceptor (Table 7), respectively.

Aminoacyl-tRNA Synthetase	Concentration (μM)<br>M9 Minimal Media + 0.4% Glucose, Aerobic	 M9 Minimal Media + 0.4% Glucose + Amino Acids, Aerobic
AlaRS	0.742	0.780
ArgRS	0.667	0.627
AsnRS	2.491	1.847
AspRS	0.577	0.519
CysRS	0.641	0.671
GluRS	3.127	2.890
GlnRS	1.335	1.352
GlyRS	0.843	0.935
HisRS	0.471	0.431
IleRS	1.674	1.888
LeuRS	1.384	1.285
LysRS	1.487	1.516
MetRS	0.906	0.814
PheRS	0.871	0.687
ProRS	0.875	0.924
SerRS	1.548	1.417
ThrRS	1.983	1.436
TrpRS	0.847	0.955
TyrRS	1.047	0.822
ValRS	1.647	1.855

Table 5: Concentrations of aminoacyl-tRNA synthetases used as constants in the optimization of aminoacyl-tRNA synthetase kinetic parameters. Data were retrieved from the Parca of the E. coli Model [1].

Amino Acid	Concentration (μM)	
	M9 Minimal Media + 0.4% Glucose, Aerobic	M9 Minimal Media + 0.4% Glucose + Amino Acids, Aerobic
Alanine	1115.7	2231.3
Arginine	286.7	6159.4
Asparagine	431.5	863.0
Aspartate	2533.0	5066.0
Cysteine	21.7	43.4
Glutamate	24900.0	49800.0
Glutamine	4020.0	8040.0
Glycine	623.5	1247.0
Histidine	73.3	146.5
Isoleucine	152.0	304.0
Leucine	152.0	339.4
Lysine	870.7	12636.5
Methionine	81.2	162.3
Phenylalanine	74.1	148.1
Proline	405.0	810.0
Serine	822.3	1644.7
Threonine	293.0	586.0
Tryptophan	21.7	387.1
Tyrosine	131.2	1873.9
Valine	305.0	610.0

Table 6: Concentrations of amino acids used as constants in the optimization of aminoacyl-tRNA synthetase kinetic parameters. Data were retrieved from the Parca of the E. coli Model [1].

Amino Acid	tRNA	Concentration (μM)	
		M9 Minimal Media + 0.4% Glucose, Aerobic	M9 Minimal Media + 0.4% Glucose + Amino Acids, Aerobic
Alanine	alaT	5.278	6.432
	alaU	5.278	6.432
	alaV	5.278	6.432
	alaW	1.410	1.696
	alaX	1.410	1.696
Arginine	argQ	4.897	5.990
	argU	2.978	3.393
	argV	4.897	5.990
	argW	1.958	2.014
	argX	1.958	2.014
	argY	4.897	5.990
	argZ	4.897	5.990
Asparagine	asnT	1.282	1.683
	asnU	1.282	1.683
	asnV	1.282	1.683
	asnW	1.282	1.683
Aspartate	aspT	3.368	4.629
	aspU	3.368	4.629
	aspV	3.368	4.629
Cysteine	cysT	6.190	6.892
Glutamate	gltT	5.102	6.746
	gltU	5.102	6.746
	gltV	5.102	6.746
	gltW	5.102	6.746
Glutamine	glnU	1.725	1.908
	glnV	2.134	2.889
	glnW	1.725	1.908
	glnX	2.134	2.889
Glycine	glyT	4.701	5.195
	glyU	4.701	5.195
	glyV	4.605	5.752
	glyW	4.605	5.752
	glyX	4.605	5.752
	glyY	4.605	5.752
Histidine	hisR	2.978	3.817
Isoleucine	ileT	3.188	4.485
	ileU	3.188	4.485
	ileV	3.188	4.485
	ileX	3.188	4.485
	ileY	3.188	4.485
Leucine	leuP	4.800	5.169
	leuQ	4.800	5.169
	leuT	4.800	5.169
	leuU	4.390	5.301
	leuV	4.800	5.169

Table 7

Amino Acid	tRNA	Concentration (μM) M9 Minimal Media + 0.4% Glucose, Aerobic	Concentration (μM) M9 Minimal Media + 0.4% Glucose + Amino Acids, Aerobic
	leuW	2.978	2.968
	leuX	8.306	8.906
	leuZ	3.802	3.446
Lysine	lysQ	1.344	1.608
	lysT	1.344	1.608
	lysV	1.344	1.608
	lysW	1.344	1.608
	lysY	1.344	1.608
	lysZ	1.344	1.608
Methionine	metT	1.684	2.173
	metU	1.684	2.173
	metV	2.282	3.088
	metW	2.282	3.088
	metY	2.282	3.088
	metZ	2.282	3.088
Phenylalanine	pheU	2.272	2.439
	pheV	2.272	2.439
Proline	proK	3.098	2.491
	proL	3.055	3.499
	proM	2.272	2.439
Serine	serT	6.153	6.839
	serU	1.215	1.431
	serV	5.094	5.407
	serW	1.586	1.935
	serX	1.586	1.935
Threonine	thrT	4.390	5.301
	thrU	4.074	6.149
	thrV	0.509	0.477
	thrW	2.467	2.915
Tryptophan	trpT	3.684	4.771
Tyrosine	tyrT	1.880	2.014
	tyrU	4.427	4.930
	tyrV	1.880	2.014
Valine	valT	3.007	3.817
	valU	3.007	3.817
	valV	3.173	3.869
	valW	2.625	2.491
	valX	3.007	3.817
	valY	3.007	3.817
	valZ	3.007	3.817

Table 7: Total (both aminoacylated and unaminoacylated forms) concentrations of tRNA isoacceptors used as constants in the optimization of aminoacyl-tRNA synthetase kinetic parameters. Data were retrieved from the Parca of the E. coli Model [1].

To describe the utilization of aminoacyl-tRNAs by ribosomes, the anticipated rate of codon reading was calculated by describing the proteome in units of number of codons read N by time t. At the start of the cell cycle, the proteome can be described as the result of having read N_0 codons by a certain time $t = 0$:

$$N(t = 0) = N_0 \tag{34}$$

By the end of the cell cycle ($t = \tau$, where τ is the doubling time), the proteome will have doubled:

$$N(t = \tau) = 2N_0 \tag{35}$$

Together, equations 34 and 35 describe exponential growth, where the change in the number of codons read as time goes on can be described by the growth rate r:

$$N(t) = N_0 e^{rt}, \quad r = \frac{\ln(2)}{\tau} \tag{36}$$

and the rate of change in the number of codons read by a certain time t is:

$$\frac{dN}{dt} = rN_0 e^{rt} = rN(t) \tag{37}$$

To describe the rate of codon reading, v_{codon}, the number of codons read during time step Δt is described as the difference in the number of codons read by time t and by a small step later $t + \Delta t$ divided by the time step:

$$v_{codon} = \frac{N(t + \Delta t) - N(t)}{\Delta t} = \frac{dN}{dt} \tag{38}$$

To express the rate of codon reading in units molar concentration per time, the right-hand side of Equation 38 is divided by Avogadro's number (N_A) and the cell's volume at time t ($V(t)$):

$$v_{codon} = \frac{dN}{dt} \frac{1}{N_A} \frac{1}{V(t)} \tag{39}$$

Since the cell's volume is not represented directly in the *E. coli* Model, but rather calculated as the cell's mass ($M(t)$, which is represented directly) divided by the cell's density (ρ), the rate of codon reading is described as:

$$v_{codon} = \frac{dN}{dt} \frac{1}{N_A} \frac{\rho}{M(t)} \tag{40}$$

Substituting Equation 37 yields:

$$
\begin{aligned}
v_{codon} &= rN(t)\frac{1}{N_A}\frac{\rho}{M(t)} \\
&= r\frac{N(t)}{M(t)}\frac{\rho}{N_A} \\
&= \frac{\ln(2)}{\tau}\frac{N_0 e^{rt}}{M_0 e^{rt}}\frac{\rho}{N_A} \\
&= \frac{\ln(2)}{\tau}\frac{N_0}{M_0}\frac{\rho}{N_A}
\end{aligned} \tag{41}
$$

where N_0 is the number of codons read to create the initial proteome and M_0 is the initial mass of the cell.

To generalize Equation 41 for the reading rate of the i-th codon type, N_0 is replaced with the number of times codon i is read to create the initial proteome:

$$
v_{codon,i} = \frac{\ln(2)}{\tau}\frac{N_{i,0}}{M_0}\frac{\rho}{N_A} \tag{42}
$$

Table 8 lists the codon reading rates obtained from Equation 42.

Amino Acid	Codon	Codon Reading Rate (μM/s) M9 Minimal Media + 0.4% Glucose, Aerobic	M9 Minimal Media + 0.4% Glucose + Amino Acids, Aerobic
Alanine	GCA	8.904	14.952
	GCC	6.296	9.943
	GCG	9.775	15.641
	GCU	12.060	20.789
Arginine	AGA	0.303	0.479
	AGG	0.104	0.157
	CGA	0.412	0.634
	CGC	7.043	11.780
	CGG	0.557	0.858
	CGU	11.908	20.553
Asparagine	AAC	11.603	19.104
	AAU	3.979	6.259
Aspartate	GAC	11.051	18.132
	GAU	10.145	16.231
Cysteine	UGC	1.778	2.838
	UGU	1.036	1.640
Glutamate	GAA	18.582	30.655
	GAG	6.120	10.034
Glutamine	CAA	3.666	5.922
	CAG	10.790	17.541
Glycine	GGA	1.315	2.008
	GGC	12.333	20.174
	GGG	1.785	2.753
	GGU	13.851	23.285
Histidine	CAC	4.423	7.334
	CAU	2.657	4.260
Isoleucine	AUA	0.424	0.646
	AUC	12.888	21.478
	AUU	7.670	12.289
Leucine	CUA	0.496	0.752
	CUC	2.320	3.646
	CUG	22.081	36.277
	CUU	2.257	3.598
	UUA	1.926	3.018
	UUG	2.216	3.475
Lysine	AAA	18.678	31.412
	AAG	5.146	8.990
Methionine	AUG (start)	1.648	2.817
	AUG (elongating)	7.923	12.849
Phenylalanine	UUC	7.845	12.932
	UUU	4.553	7.235
Proline	CCA	2.242	3.601
	CCC	0.754	1.144
	CCG	8.985	14.698
	CCU	1.783	2.931
Serine	AGC	4.921	7.992
	AGU	1.536	2.419
	UCA	1.299	2.015
	UCC	4.321	7.120
	UCG	1.509	2.321
	UCU	5.368	8.997
Threonine	ACA	1.407	2.235
	ACC	10.066	16.294
	ACG	2.829	4.407
	ACU	6.486	10.984
Tryptophan	UGG	3.524	5.619
Tyrosine	UAC	6.143	9.996
	UAU	3.993	6.266
Valine	GUA	6.085	10.446
	GUC	3.808	6.160
	GUG	6.921	11.079
	GUU	11.874	20.307

Table 8: Codon reading rates used as constants in the optimization of aminoacyl-tRNA synthetase kinetic parameters. Data were calculated according to Equation 42, where $N_{i,0}$, M_0, ρ, N_A, and τ were retrieved from the Parameter Calculator (Parca) of the E. coli Model [1].

To convert the reading rate of codons into the rate of aminoacyl-tRNA utilization by ribosomes, the aminoacyl-tRNA abundance matrix $\boldsymbol{T}$ is populated as described by Equation 26.

$$\boldsymbol{T} = \begin{bmatrix} (1-f_1)T_1 & (1-f_1)T_1 & — & (1-f_1)T_1 \\ (1-f_2)T_2 & (1-f_2)T_2 & — & (1-f_2)T_2 \\ \vdots & \vdots & & \vdots \\ (1-f_n)T_n & (1-f_n)T_n & — & (1-f_n)T_n \end{bmatrix} \tag{43}$$

where T_i is the total amount of the i-th tRNA isoacceptor (ie. both aminoacylated and unaminoacylated

forms), $(1 - f_i)$ is the fraction of $[T_i]$ that exists in the aminoacylated form, and n is the number of tRNA isoacceptors that interact with the aminoacyl-tRNA synthetase described by this objective function.

Then, the appropriate codon-to-tRNA anticodon mapping matrix $\boldsymbol{M}$ is retrieved from Equations 5 through 24 (depending on the amino acid family) and used to calculate $\boldsymbol{D}$ according to Equation 27:

$$\boldsymbol{D} = \boldsymbol{M} \otimes \boldsymbol{T} \tag{44}$$

where $\otimes$ is the element-wise multiplication operation.

Normalizing the columns of $\boldsymbol{D}$ according to Equation 28 yields the codon reading load distribution matrix $\bar{\boldsymbol{D}}$, which has elements:

$$\bar{\boldsymbol{D}}_{\boldsymbol{ij}} = \frac{\boldsymbol{D}_{\boldsymbol{ij}}}{\sum_{i=1}^{n} \boldsymbol{D}_{\boldsymbol{ij}}} \tag{45}$$

where n is the number of tRNA isoacceptors that interact with the aminoacyl-tRNA synthetase described by this objective function.

Finally, the codon reading rates (from Equation 41) for the codons listed along the columns of matrix M are assembled into a vector v_{codons}. The dot product of $\bar{\boldsymbol{D}}$ and v_{codons} yields the rate of utilization each aminoacyl-tRNA isoacceptor by ribosomes.

$$v_D = \bar{\boldsymbol{D}} \cdot v_{codons} \tag{46}$$

from which the utilization rate of the i-th aminoacyl-tRNA isoacceptor is the i-th element of v_D.

Taken together, the sum of the square of the relative errors between the tRNA aminoacylation and aminoacyl-tRNA utilization rates of all tRNA isoacceptors yields the steady-state error:

$$e_{ss} = \sum_{i=1}^{n} \left(1 - \frac{v_{A,i}}{v_{D,i}}\right)^2 \tag{47}$$

where n is the number of tRNA isoacceptors that interact with the aminoacyl-tRNA synthetase described by this objective function.

When testing candidate kinetic parameter solutions in simulations, it was observed that the best solutions continued to achieve steady-state (i.e. v_A matched v_D) even at low aminoacyl-tRNA synthetase concentrations. Consequently, a second term was incorporated to account for the minimum aminoacyl-tRNA synthetase concentration, $[E_{min}]$:

$$e_{ss} = \sum_{i=1}^{n} \left(1 - \frac{v_{A,avg,i}}{v_{D,i}}\right)^2 + \left(1 - \frac{v_{A,min,i}}{v_{D,i}}\right)^2 \tag{48}$$

where $v_{A,avg,i}$ (previously $v_{A,i}$ in Equation 47) is the rate of aminoacyl of the i-th tRNA isoacceptor at the average aminoacyl-tRNA synthetase concentration ($[E_{avg}]$) and $v_{A,min,i}$ is the rate of aminoacylation of the i-th tRNA isoacceptor at $[E_{min}]$:

$$v_{A,avg,i} = k_{cat}[E_{avg}]\frac{[A]}{K_A + [A]}\frac{\frac{f_{avg,i}\cdot[T_i]}{K_{T,i}}}{1 + \sum_j^n \frac{f_{avg,j}\cdot[T_j]}{K_{T,j}}}$$
$$v_{A,min,i} = k_{cat}[E_{min}]\frac{[A]}{K_A + [A]}\frac{\frac{f_{min,i}\cdot[T_i]}{K_{T,i}}}{1 + \sum_j^n \frac{f_{min,j}\cdot[T_j]}{K_{T,j}}} \quad (49)$$

where f_{avg} and f_{min} are the fractions of the total tRNA T that exist in the unaminoacylated form at $[E_{avg}]$ and $[E_{min}]$, respectively. Notably, both $v_{A,avg,i}$ and $v_{A,min,i}$ are anticipated to equal $v_{D,i}$ at steady-state, with the difference in the aminoacylated fraction ($1 - f_{avg}$ and $1 - f_{min}$) absorbing the bulk of the change in $[E]$.

In addition, the availability of the constant parameters, v_D, $[E]$, $[A]$, and $[T]$, in two different growth conditions (described in Tables 8, 5, 6, and 7, respectively) provided an opportunity to reduce the solution space by minimizing the steady-state error in both conditions.

$$e_{ss} = \sum_{j=1}^{c}\left[\sum_{i=1}^{n}\left(1 - \frac{v_{A,avg,ij}}{v_{D,ij}}\right)^2 + \left(1 - \frac{v_{A,min,ij}}{v_{D,ij}}\right)^2\right] \quad (50)$$

where $c = 2$ is the number of growth conditions used in all parameter optimization problems (the E. coli Model's representation of M9 Minimal Media supplemented with 0.4% Glucose, and M9 Minimal Media supplemented with 0.4% Glucose and amino acids).

2.2.2 Regularization

As an underdetermined system, the aminoacyl-tRNA synthetase kinetic parameter optimization problem generated many solutions, some with needlessly large estimates of kinetic parameters. Thus, a regularization term was incorporated to prefer smaller kinetic parameters that could achieve the same output.

Inspecting Equation 49 revealed that the K_T's (the Michaelis-Menten constant describing the binding affinity between tRNAs and their aminoacyl-tRNA synthetases) would be the most appropriate parameters to apply the regularization to because their primary role is to distribute the aminoacylation capacity $k_{cat}[E]\frac{[A]}{K_A+[A]}$ across the different tRNA isoacceptors.

Thus, the sum of the K_T's was scaled with weight $w_r = 10^{-9}$ and minimized, as described in Equation 32, where R is:

$$R = \sum_{i=1}^{n} K_{T,i} \quad (51)$$

where n is the number of tRNA isoacceptors that interact with the aminoacyl-tRNA synthetase described by this objective function.

2.2.3 Bounds Penalty

As a fraction, f must range between 0 and 1; this requirement is described by the constraints of the optimization:

$$
\begin{aligned}
\text{minimize} \quad & e_{ss} + (w_r \cdot R) + (w_b \cdot B) \\
\text{subject to} \quad & 0 < f_{avg,i} < 1, \quad i = 1, ..., n \\
& 0 < f_{min,i} < 1, \quad i = 1, ..., n
\end{aligned} \tag{52}
$$

where n is the number of tRNA isoacceptors that interact with the aminoacyl-tRNA synthetase described by this objective function.

As an underdetermined system, the optimization problem generated many solutions, some with f values close to 0 or 1, which led to unstable simulations. Thus, a bounds penalty was incorporated to prefer f values that could achieve the same output with greater stability. A 5% buffer from either extreme was described the term $w_b \cdot B$ in Equation 32, where $w_b = 10^{-9}$ is the weight and B is:

$$
B = \sum_{i=1}^{n} \left(\frac{1}{f_{avg,i} - 0.05} + \frac{1}{0.95 - f_{avg,i}} \right) + \left(\frac{1}{f_{min,i} - 0.05} + \frac{1}{0.95 - f_{min,i}} \right) \tag{53}
$$

where n is the number of tRNA isoacceptors that interact with the aminoacyl-tRNA synthetase described by this objective function, and $B(f)$ has the shape:

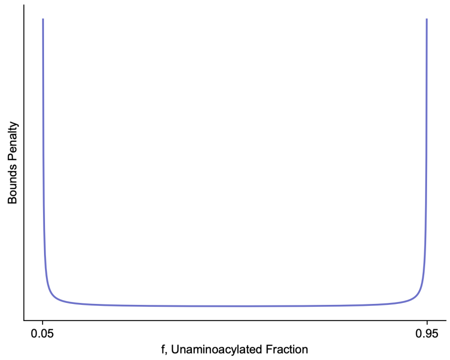

Figure 3: Profile of bounds penalty applied to f, the fraction of unaminoacylated tRNA.

2.2.4 Complete Form

Taken together, the complete form of the objective function for the set of kinetic parameters that describe one aminoacyl-tRNA synthetase is:

$$
\begin{aligned}
\text{minimize} \quad & \sum_{j=1}^{c} \left[\sum_{i=1}^{n} \left(1 - \frac{v_{A,avg,ij}}{v_{D,ij}}\right)^2 + \left(1 - \frac{v_{A,min,ij}}{v_{D,ij}}\right)^2 \right] \\
& + w_r \cdot \sum_{i=1}^{n} K_{T,i} \\
& + w_b \cdot \sum_{i=1}^{n} \left(\frac{1}{f_{avg,i} - 0.05} + \frac{1}{0.95 - f_{avg,i}} \right) + \left(\frac{1}{f_{min,i} - 0.05} + \frac{1}{0.95 - f_{min,i}} \right) \\
\text{subject to} \quad & 0 < f_{avg,i} < 1, \quad i = 1, ..., n \\
& 0 < f_{min,i} < 1, \quad i = 1, ..., n
\end{aligned} \tag{54}
$$

where n is the number of tRNA isoacceptors that interact with the aminoacyl-tRNA synthetase described by this objective function, $c = 2$ is the number of growth conditions used in all parameter optimization

problems (the E. coli Model's representation of M9 Minimal Media supplemented with 0.4% Glucose, and M9 Minimal Media supplemented with 0.4% Glucose and amino acids), $v_{A,avg}$ and $v_{A,min}$ are the rates of tRNA aminoacylation at the average and minimum aminoacyl-tRNA synthetase concentrations, respectively (as described by Equation 49), v_D is the rate of aminoacyl-tRNA utilization by ribosomes (as described by Equation 46), f_{avg} and f_{min} are the fractions of the total tRNA T that exist in the unaminoacylated form at $[E_{avg}]$ and $[E_{min}]$, respectively, and K_T is the Michaelis-Menten constant describing the binding affinity between the aminoacyl-tRNA synthetase and the unaminoacylated tRNAs.

2.3 Generation of Solutions

2.3.1 Random Initialization

To explore the solution space, optimization of the minimization problem described in Equation 54 was performed many times with random initialization of the initial solution. For simplicity, tRNA isoacceptors that have the same codon interaction patterns (according to Equations 5 through 24) are assigned a single, shared K_T.

Algorithm 1: Random Initialization of One Set of Kinetic Parameters for One Aminoacyl-tRNA synthetase

Input : $v_{codons,c}$, Codon reading rates at growth condition c
Input : $[E_c]$, Concentration of aminoacyl-tRNA synthetase at growth condition c
Input : $[A_c]$, Concentration of amino acids at growth condition c
Input : $[T_{i,c}]$, Total concentration of the i-th tRNA at growth condition c
/* Initialize K_A and K_T's */
$K_A \leftarrow$ a random number selected from a uniform distribution between $[A_{c=1}]$ and $[A_{c=2}]$
for $K_{T,i}$ **do**
    $K_{T,i} \leftarrow 10^x$ where x is a random number selected from a uniform distribution between 1 and 6. tRNA isoacceptors that have the same codon interaction patterns (according to Equations 5 through 24) are assigned a single, shared K_T.
/* Initialize f's */
for $f_{avg,i}$ **do**
    $f_{avg,i} \leftarrow$ a random number selected from a uniform distribution between 0.1 and 0.9
for $f_{min,i}$ **do**
    $f_{min,i} \leftarrow$ a random number selected from a uniform distribution between 0.1 and 0.9
/* Estimate a compatible k_{cat} in each growth condition */
for *condition, c* **do**
    /* Describe saturation of substrates */
    $sat_A = \frac{[A_c]}{K_A+[A_c]}$
    $sat_T = \frac{\sum_{i=1}^{n} \frac{f_{avg,i,c}\cdot[T_{i,c}]}{K_{T,i}}}{1+\sum_{i=1}^{n} \frac{f_{avg,i,c}\cdot[T_{i,c}]}{K_{T,i}}}$
    /* Calculate the k_{cat} */
    $k_{cat} = \frac{v_{codons,c}}{[E_c]\cdot sat_A \cdot sat_T}$
/* Initialize the k_{cat} at the maximum estimate */
$k_{cat} \leftarrow$ the maximum of $k_{cat,1}$ and $k_{cat,2}$
Result: One set of kinetic parameters has been randomly initialized.

2.3.2 Parametric Sweep on the Minimum Aminoacyl-tRNA Synthetase Concentration

To assess the impact of the minimum aminoacyl-tRNA synthetase concentration, a parametric sweep was performed on $[E_{min}]$. To inform the range of this sweep, a sample set of 200 simulations (100 in M9 Minimal Media + 0.4% Glucose, and 100 in M9 Minimal Media + 0.4% Glucose + Amino Acids) was run in the prior E. coli Model [1], which assumes unlimited tRNA aminoacylation, to gauge the dynamic range of aminoacyl-tRNA synthetases. Each set of 100 simulations was composed of 20-generation runs initialized at 5 random seeds. The commands used to run these sample sets were:

```
python runscripts/manual/runSim.py --no-trna-charging --translation-supply -v condition 0 0 -g 20 -i 5
python runscripts/manual/runSim.py --no-trna-charging --translation-supply -v condition 2 2 -g 20 -i 5
```

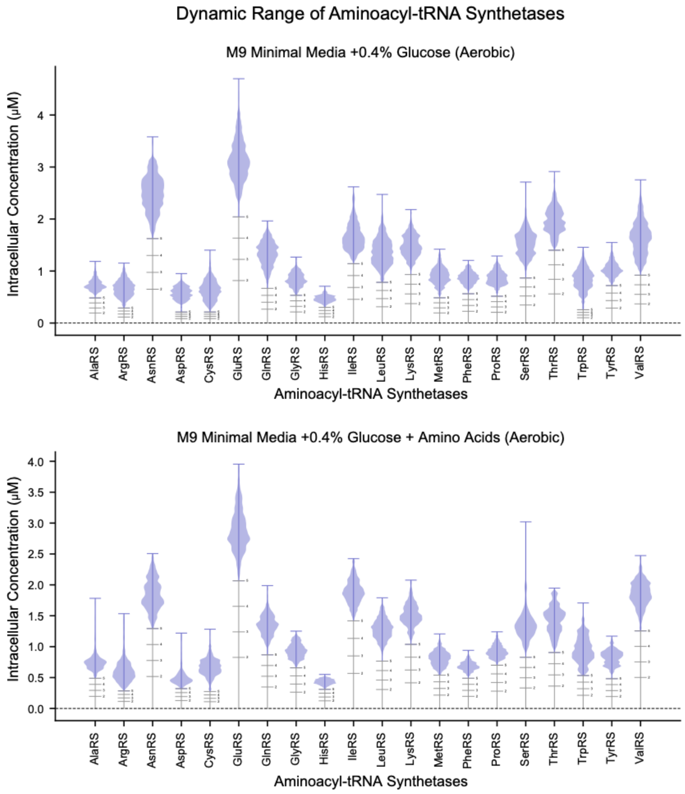

Figure 4: Range of aminoacyl-tRNA synthetase concentrations observed from simulations run in the Prior Model at two growth conditions: M9 Minimal Media + 0.4% Glucose, and M9 Minimal Media + 0.4% Glucose + Amino Acids. The tick marks labeled '2' through '5' (reported in Table 9) indicate the values of the minimum aminoacyl-tRNA synthetase concentration $[E_{min}]$ used in Equation 49. Note that Figure 4 and Figure S3 are the same.

In order to prepare the tRNA aminoacylation system to remain robust at low levels of aminoacyl-tRNA synthetase (which can be variable between cells), the $[E_{min}]$ was allowed to be equal to or less than the observed minimum (labeled '5' in Figure 4). To do so, the distance between the observed minimum ('5') and 0 μM (which is the lowest feasible value) was divided into five equal parts for each aminoacyl-tRNA synthetase. Of these parts, the values corresponding to the highest four values (labeled '2', '3', '4', and '5' in Figure 4) were identified and used as the four values of $[E_{min}]$ in the parametric sweep on the minimum aminoacyl-tRNA synthetase concentration (Table 9). The aminoacyl-tRNA synthetase concentrations corresponding to '1' was found to lead to extremely low intracellular concentrations that could not generate viable aminoacyl-tRNA synthetase kinetic solutions, and was therefore not used.

Next, the aminoacyl-tRNA synthetase kinetic solutions with the minimum objective value was identified for each aminoacyl-tRNA synthetase at each $[E_{min}]$ sweep value. The k_{cat}s corresponding to these minimum-objective solutions are depicted Figure 3H of the main text.

Finally, to select the best performing aminoacyl-tRNA synthetase kinetic parameters, the behavior of these minimum-objective solutions were assessed in test simulations of a nutritional downshift experiment from rich (M9 Minimal Media + 0.4% Glucose + Amino Acids) to poor (M9 Minimal Media + 0.4% Glucose) media. These tests revealed that the aminoacyl-tRNA synthetase kinetic parameter set corresponding to $[E_{min}]$ sweep value '4' demonstrated balanced exploration of aminoacylated fraction values across all tRNAs. For example, the aminoacyl-tRNA synthetase kinetic solutions generated from '2' caused aminoacylation fractions to remain unresponsive to the downshift test, while those generated from '5' caused aminoacylation fractions to fall close to 0%.

The command used to perform the nutritional downshift experiment was:
`models/ecoli/sim/variants/trna_synthetase_kinetics_test.py`

Aminoacyl-tRNA Synthetase	Growth Condition	Concentrations (μM)			
		Sweep Value '2'	Sweep Value '3'	Sweep Value '4'	Sweep Value '5'
AlaRS	⊖	0.19327	0.28991	0.38655	0.48319
AlaRS	⊕	0.19516	0.29274	0.39032	0.48791
ArgRS	⊖	0.11555	0.17332	0.23110	0.28887
ArgRS	⊕	0.11368	0.17051	0.22735	0.28419
AsnRS	⊖	0.64972	0.97459	1.29945	1.62431
AsnRS	⊕	0.51728	0.77592	1.03456	1.29320
AspRS	⊖	0.08459	0.12688	0.16917	0.21146
AspRS	⊕	0.12813	0.19219	0.25626	0.32032
CysRS	⊖	0.08476	0.12713	0.16951	0.21189
CysRS	⊕	0.10953	0.16429	0.21906	0.27382
GluRS	⊖	0.81635	1.22453	1.63270	2.04088
GluRS	⊕	0.82649	1.23974	1.65299	2.06623
GlnRS	⊖	0.26638	0.39957	0.53277	0.66596
GlnRS	⊕	0.34739	0.52109	0.69478	0.86848
GlyRS	⊖	0.21354	0.32031	0.42708	0.53385
GlyRS	⊕	0.26325	0.39487	0.52649	0.65811
HisRS	⊖	0.12067	0.18101	0.24135	0.30168
HisRS	⊕	0.12385	0.18577	0.24769	0.30961
IleRS	⊖	0.45631	0.68446	0.91261	1.14077
IleRS	⊕	0.56688	0.85031	1.13375	1.41719
LeuRS	⊖	0.31302	0.46953	0.62604	0.78254
LeuRS	⊕	0.30649	0.45974	0.61299	0.76623
LysRS	⊖	0.37325	0.55988	0.74651	0.93313
LysRS	⊕	0.41491	0.62236	0.82981	1.03727
MetRS	⊖	0.19440	0.29161	0.38881	0.48601
MetRS	⊕	0.21574	0.32361	0.43148	0.53935
PheRS	⊖	0.22535	0.33803	0.45071	0.56339
PheRS	⊕	0.19535	0.29302	0.39069	0.48837
ProRS	⊖	0.20627	0.30940	0.41253	0.51567
ProRS	⊕	0.27986	0.41980	0.55973	0.69966
SerRS	⊖	0.34654	0.51981	0.69308	0.86635
SerRS	⊕	0.33143	0.49714	0.66285	0.82857
ThrRS	⊖	0.56011	0.84017	1.12023	1.40029
ThrRS	⊕	0.36196	0.54294	0.72391	0.90489
TrpRS	⊖	0.10218	0.15327	0.20436	0.25545
TrpRS	⊕	0.21300	0.31950	0.42600	0.53250
TyrRS	⊖	0.28801	0.43201	0.57602	0.72002
TyrRS	⊕	0.19282	0.28923	0.38564	0.48205
ValRS	⊖	0.36797	0.55195	0.73593	0.91991
ValRS	⊕	0.50202	0.75302	1.00403	1.25504

Table 9: Parametric sweep on minimum aminoacyl-tRNA synthetase abundance. The parametric sweep on $[E_{min}]$ in Equation 49 was performed using the above values. The sweep values (2 through 5) refer to increment between the minimum observed aminoacyl-tRNA synthetase concentration and 0 μM (shown in Figure 4). Growth conditions: ⊖ = M9 Minimal Media + 0.4% Glucose, Aerobic, ⊕ = M9 Minimal Media + 0.4% Glucose + Amino Acids, Aerobic.

2.3.3 Comprehensive Algorithm

Optimization was performed using SciPy's `minimize` function with the Powell method. At least 100 iterations were performed for each aminoacyl-tRNA synthetase problem.

Algorithm 2: Optimization of Aminoacyl-tRNA Synthetase Kinetic Parameters

Input : $v_{codons,c}$, Codon reading rates at growth condition c
Input : $[E_{avg,c}]$, Average concentration of aminoacyl-tRNA synthetase at growth condition c
Input : $[E_{min,c}]$, Minimum concentration of aminoacyl-tRNA synthetase at growth condition c
Input : $[A_c]$, Concentration of amino acids at growth condition c
Input : $[T_{i,c}]$, Total concentration of the i-th tRNA at growth condition c

```
/* Treat each aminoacyl-tRNA synthetase as independent optimization problems */
```

for *each aminoacyl-tRNA synthetase* **do**
- Retrieve the constants: $[E_{avg,c}]$, $[E_{min,c}]$, $[A_c]$, $[T_{i,c}]$, and $v_{codons,c}$.
- **for** *each value of* $E_{x,min,c}$ *(listed in Table 9)* **do**
  - **for** *iteration* **do**
    - Generate a random initial solution (Algorithm 1.
    - Call SciPy `minimize`, using the objective function described by Equation 54 and the constant parameters.
    - Save solution with its objective value.
  - Identify the minimum-objective solution.

Result: Candidate solutions of aminoacyl-tRNA synthetase kinetic parameters are generated.

3 Simulation

The model described in Section 1 was implemented into the polypeptide elongation model of the E. coli Model published in 2020 [1]. A summary-level flowchart of the computational steps that take place within each time step is shown in Figure 1C of the main text, where the model described in Section 1 impacts the polypeptide elongation model during the estimation of ribosome steps, determination of feasibility, reconciliation of kinetic and sequence solutions, and the update of molecule abundances 3.4.

These developments occurred in the following files in the E. coli Model repository:

`wcEcoli` Path	File
`models/ecoli/processes`	`polypeptide_elongation.py`
`reconstruction/ecoli/dataclasses/process`	`transcription.py`
`reconstruction/ecoli/dataclasses/process`	`translation.py`
`reconstruction/ecoli/dataclasses`	`relation.py`
`reconstruction/ecoli/flat`	`trna_charging_kinetics.tsv`
`wholecell/utils`	`_trna_charging.pyx`

Table 10: Table of files for simulation.

3.1 Estimation of Ribosome Steps

To estimate the anticipated codon reading rate during each time step, the number of times each codon type appears in the upcoming codon sequence of all ribosomes is computed.

Algorithm 3: Estimation of Ribosome Steps

Input : n, Anticipated number of ribosome steps per ribosome
Input : Δt, Time step
`/* Calculate anticipated number of codon reading events */`
for *each ribosome,* r **do**
  Count the number of appearances of each codon type i in the next n steps of all active ribosomes
`/* Assume constant codon reading rate */`
for *each codon,* i **do**
  Divide the total number of appearances of codon i by Δt
Result: The anticipated reading rate of each codon type is estimated.

3.2 Determination of Feasibility

To determine the number of feasible ribosome steps, the tRNA aminoacylation and codon reading cycle was described as a system of ODEs and the initial value problem was solved at each time step by the Runge–Kutta method. The resulting solution yields the kinetically feasible number of codon reading events that can occur in each time step.

Algorithm 4: Determination of Feasibility

`/* Each` i`-th aminoacyl-tRNA synthetase interacts with its amino acid. */`

Input : $n_{E,i}$, Number of available i-th aminoacyl-tRNA synthetase in the cell

Input : $n_{A,i}$, Number of available amino acids cognate to the i-th aminoacyl-tRNA synthetase in the cell

Input : $k_{cat,i}$, Rate of catalysis of i-th aminoacyl-tRNA synthetase

Input : $K_{A,i}$, Michaelis-Menten constant describing the binding affinity between the i-th aminoacyl-tRNA synthetase and its cognate amino acid

`/* Each` j`-th tRNA exists in the unaminoacylated and aminoacylated forms. */`

Input : $n_{T^d,j}$, Number of available unaminoacylated j-th tRNA in the cell

Input : $n_{T^a,j}$, Number of available aminoacylated j-th tRNA in the cell

Input : $K_{T,j}$, Michaelis-Menten constant describing the binding affinity between the j-th unaminoacylated tRNA and its corresponding aminoacyl-tRNA synthetase

`/* Codon reading rate of each codon type is described in one vector. */`

Input : v_{codons}

`/* Cellular parameters. */`

Input : v, Cell volume

Input : N_A, Avogadro's number

Input : Δt, Time step

`/* Convert Michaelis-Menten constants to units of number of molecules. */`

$K_A = K_A \cdot N_A \cdot v$

$K_T = K_T \cdot N_A \cdot v$

`/* Prepare the codon reading load distribution matrix */`

for *each aminoacyl-tRNA synthetase,* i **do**

1. Populate aminoacyl-tRNA abundance matrix $\boldsymbol{T}_i$ as described by Equation 26 with the abundances of aminoacyl-tRNAs $n_{T^a,j}$.

2. Retrieve codon-to-tRNA anticodon mapping matrix $\boldsymbol{M}_i$ for the i-th aminoacyl-tRNA synthetase as described by 5 through 24 (depending on the amino acid family).

3. Calculate matrix $\boldsymbol{D}_i$ according to Equation 27:

$\boldsymbol{D}_i = \boldsymbol{M}_i \otimes \boldsymbol{T}_i$ where $\otimes$ is the element-wise multiplication operation.

4. Normalize the columns of $\boldsymbol{D}_i$ according to Equation 28, to yield the codon reading load distribution matrix $\bar{\boldsymbol{D}}_{\boldsymbol{i}}$. Each matrix $\bar{\boldsymbol{D}}$ has elements:

$\bar{\boldsymbol{D}}_{\boldsymbol{nm}} = \frac{\boldsymbol{D}_{\boldsymbol{nm}}}{\sum_{n=1}^{N} \boldsymbol{D}_{\boldsymbol{nm}}}$ where N is the number of rows in $\boldsymbol{D}$.

`/* Describe tRNA aminoacylation and codon reading as a system of ODEs */`

for *each aminoacyl-tRNA synthetase,* i **do**

1. Describe the rate of aminoacylation for each j-th tRNA (that is a substrate of the i-th aminoacyl-tRNA synthetase) according to Equation 4.

$v_{aminoacylation,j} = k_{cat,i} \cdot n_{E,i} \cdot \frac{n_{A,i}}{K_{A,i}+n_{A,i}} \frac{\frac{n_{T^d,j}}{K_{T,j}}}{1+\sum_{j=1}^{n} \frac{n_{T^d,j}}{K_{T,j}}}$ where n is the number of tRNA isoacceptors for the i-th aminoacyl-tRNA synthetase.

2. Assemble the codon reading rate for the codons read by aminoacyl-tRNA synthetase i from v_{codons} into a vector, $v_{codon,i}$.

3. Describe the rate of ribosome utilization of all aminoacyl-tRNA isoacceptors of the i-th aminoacyl-tRNA synthetase according to Equation 29.

$v_{utilization} = \bar{\boldsymbol{D}}_{\boldsymbol{i}} \cdot v_{codon,i}$

`/* Retrieve the kinetically feasible number of codon reading events */`

Call the `solve_ivp` program from SciPy to solve the initial value problem during Δt.

Stochastically round the resulting number of codon reading events that occurred during Δt for each codon type.

Result: The kinetically-feasible number of reading events for each codon type is estimated.

By using the abundance of aminoacyl-tRNAs to calculate the codon reading load distribution matrix $\bar{\boldsymbol{D}}$,

the distribution of codon reading load across different aminoacyl-tRNA isoacceptors is proportional to the relative abundances of aminoacyl-tRNAs.

3.3 Reconciliation of Kinetic and Sequence Solutions

3.3.1 Motivation

In the updated model reported in this study, two different calculations each yield an estimation of the number of codons read during a given time step:

1. a kinetics-based model of the tRNA aminoacylation cycle that obeys the kinetic capacity of aminoacyl-tRNA synthetases to produce aminoacyl-tRNAs (described in Section 3.2), and
2. a sequence-based model of ribosome procession that obeys the codon sequence order that ribosomes encounter on mRNAs (which is the method used in the prior E. coli Model [1]).

To demonstrate each of their outputs during time step Δt in a two-codon system:

1. The kinetics-based model yields the reading rate of each codon. For example,
   - codon A was read at v_A codons per second
   - codon B was read at v_B codons per second

   Multiplying by the time step t results in the number of times each codon type was read:
   - codon A was read n_A times, where $n_A = v_A \cdot \Delta t$
   - codon B was read n_B times, where $n_B = v_B \cdot \Delta t$
2. The sequence-based model yields the number and order of codon reading events. For example,
   - Ribosome 1 read codon A, then codon B, then codon A;
   - Ribosome 2 read codon B, then codon A, then codon A;
   - ... and so on until Ribosome n read codon A, then codon B, then codon B.

   Collectively, the ribosomes read codon A n_A times and codon B n_B times.

Each model approaches the calculation of the number of codons read during t from different perspectives – the kinetic capacity of aminoacyl-tRNA synthetases and the sequence order of codons – and thereby, their estimations of n_A and n_B may not agree. In the E. coli Modeling framework, both perspectives are valuable and must be honored. As a result, the purpose of the Reconciliation Program is to reconcile any disagreements between the kinetics-based and sequence-based solutions.

3.3.2 Tools

To reconcile disagreements in the number of codons read, two points of flexibility were identified and used as tools for the reconciliation task: the flexibility offered by the codon sequence and the tRNA pools.

Flexibility Offered by the Codon Sequence

For each ribosome, the position of the ribosome and the codon sequence of the mRNA could be used to survey the identities of the surrounding codons. For instance, if the sequence-based model overestimated (compared to the kinetics-based model) the number times codon A was read by 1 instance – and if codon A was the final codon read by the i-th ribosome – then the disagreement could be resolved by reversing the last codon reading event by ribosome i.

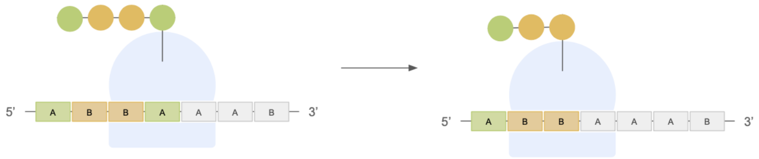

Figure 5: Flexibility offered by the codon sequence. In this scenario, the overestimation of codon A (green) readings by the sequence-based model can be resolved by reversing the last codon A reading event by the ribosome (blue).

The result of resolving all sequence-based model overestimations through reversing codon reading events is a profile of ribosome positions on mRNAs that obey the kinetic limits of tRNA aminoacylation during time step Δt.

Flexibility offered by the tRNA pools

For each tRNA isoacceptor, the pools of aminoacylated and unaminoacylated tRNAs could be used to absorb differences in codon reading events. For instance, the profile of ribosome positions on mRNAs from the previous example may result in a maximum of $n_{B,max}$ instances of codon B readings – no more because that would require reading codon A to reach the next instance of codon B (according to the sequence order of codons) and thereby compromising the kinetic limit of codon A readings.

If the kinetics-based solution n_B is greater than $n_{B,max}$, then the kinetics-based model has overestimated (compared to the sequence-based model) the number of times codon B was read. If the overestimation occurred by 1 codon B reading event – and if at least 1 copy of the tRNA isoacceptor that interacts with codon B exists in the unaminoacylated form – then the disagreement can be resolved by returning the unaminoacylated tRNA to its aminoacylated form, which reverses the final codon reading event in the kinetics-based model.

If no copies of the tRNA isoacceptor exist in the unaminoacylated form, then the assumption of a constant codon reading rate in the kinetics-based model (Algorithm 3) must have been violated. This can be seen in a linear representation of the tRNA aminoacylation cycle.

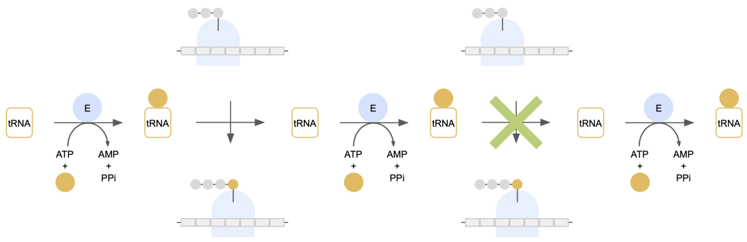

Figure 6: Flexibility offered by tRNA pools. In this scenario, the kinetics-based model proposes that an unaminoacylated tRNA (yellow rectangle) can be aminoacylated three times and de-aminoacylated two times during a particular time step. In contrast, the sequence-based model has calculated that the final codon-reading event was not feasible according to the sequence order of codons on mRNAs (green X). This overestimation can be resolved by reversing the last codon B reading event (green X) by the ribosome (blue), and eliminating all downstream reactions, such as the final tRNA aminoacylation reaction by the aminoacyl-tRNA synthetase (E).

In Figure 6, the final tRNA aminoacylation event assumed the existence of an unaminoacylated tRNA. However, the reversal of the final codon reading event nullifies the existence of the unaminoacylated tRNA and all downstream reactions.

The result of resolving kinetics-based model overestimations through reversing codon-reading events (and downstream reactions) is a solution of the number of tRNA aminoacylation and codon reading events that obey the order of codons encountered by ribosomes during time step Δt.

3.3.3 Strategy

Whereas utilizing the flexibility offered by the codon sequence preserves the kinetic solution, adjusting the tRNA pools can result in a reduced kinetic solution than originally intended if tRNA aminoacylation events are reversed (Section 3.3.2). As such, the two tools were applied in a preferential manner: resolve as many disagreements as possible using the flexibility of codon sequences, then resolve any remaining disagreements using the flexibility of tRNA pools.

Stage 1: Using the flexibility of the codon sequence
Disagreements in the number of codons read by the kinetics-based and sequence-based models can occur in both orientations: the sequence-based solution may overestimate the reading of some codons and underestimate the reading of other codons. When resolving these disagreements using the flexibility of the codon sequence, resolving sequence-based underestimations first (by processing ribosomes towards the 5' end) followed by the overestimations (by processing ribosomes towards the 3' end) ensures that the kinetic limit of codon reading events is always honored.

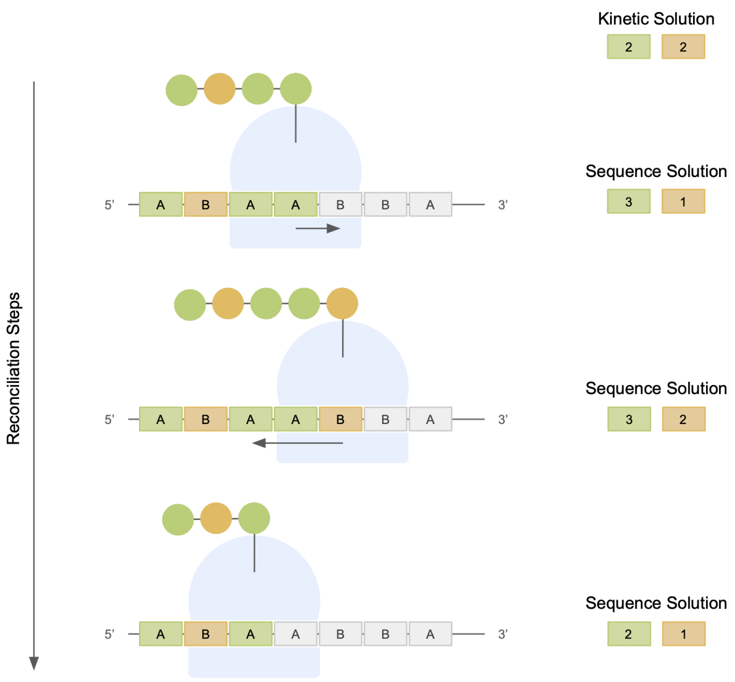

Figure 7: Order of resolving disagreements. This scenario demonstrates the reconciliation steps taken to resolve both an overestimate (codon A, green) and underestimate (codon B, yellow) of the sequence-based solution.

In the scenario depicted in Figure 7, the kinetics-based solution determined that two reading events each for codons A (green) and B (yellow) occurred during time step Δt. Meanwhile, the sequence-based solution determined that three codon A reading events and one codon B reading event occurred. Resolving the sequence-based underestimation first (codon B is read 1 time in the sequence solution, which is less than its 2 reading events in the kinetic solution), followed by the sequence-based overestimation next (codon A is read 3 times in the sequence solution, which is greater than its 2 readings in the kinetic solution) ensures that the resulting solution honors the kinetic limit of codon reading events (codon A is read 2 times in both solutions and codon B is read 1 less time in the sequence solution than the kinetic solution).

Selecting the Best Compromise

When using the flexibility of the codon sequence to reconcile disagreements in the number of codons read between the two models, two key characteristics of codon sequences made it possible to arrive at many different solutions:

1. Tens of thousands of ribosomes: At each time step, there are 10,000s of active ribosomes processing on mRNAs, each with a unique sequence of codons flanking its current location. As a result, for each disagreement of codon i, there are many candidate ribosomes that can undergo the process depicted in Figure 7.

2. Presence of 'in-between' codons: In some cases, the next available instance of codon i is not immediately accessible (as it was for the ribosome in Figure 7, which took +1 and -1 steps). Rather, the ribosome may need to take multiple steps (as depicted in Figure 8) to arrive at the next instance of codon i. The presence of the 'in-between' codons – the two instances of codon C (pink), for example – may introduce further disagreements between the two models. Consequently, encountering further disagreements while pursuing global reconciliation is anticipated.

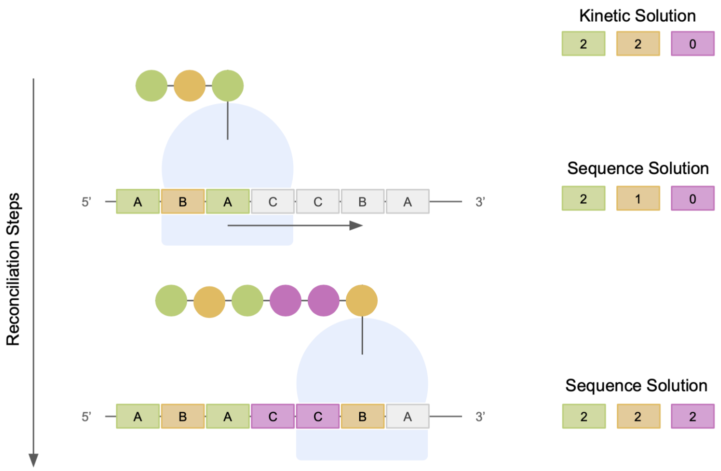

Figure 8: Presence of 'in-between' codons. This scenario demonstrates the progress towards resolution of an underestimate (codon B, yellow) of the sequence-based solution. The presence of 'in-between' codon C (pink) introduces new disagreements that did not exist at the start of the reconciliation.

As a result of these characteristics, each random selection of a ribosome to use (among many candidate ribosomes that could be used for resolving a codon disagreement) affects the landscape of ribosome positions and thereby impacts the options that are available for resolving the next codon disagreement. To resolve as many disagreements as possible in Stage 1, an iterative approach that enables sampling of the reconciliation solution space was developed. To compare candidate solutions, it was necessary to design a metric that would measure a solution's degree of success. This metric, called the compromise C, reflects the total number of disagreements remaining in a solution:

$$C = \sum_{i=0}^{n} |k_i - s_i| \tag{55}$$

where k_i is the number of times codon i is read according to the kinetics-based solution, s_i is the number of times codon i is read according to the sequence-based solution, and n is the number of codon types. A compromise of $C = 0$ indicates a completely reconciled state.

Stage 2: Using the flexibility of tRNA pools

Empirically, it was found that 75% of initial disagreements could be resolved in Stage 1. To resolve the remaining disagreements, the flexibility offered by the tRNA pools was used.

Due to the design of resolving sequence-based underestimations first (by processing ribosomes towards the 5' end) followed by the overestimations (by processing ribosomes towards the 3' end), any disagreements remaining by Stage 2 were sequence-based underestimations. These underestimations were resolved by reversing the appropriate number of final codon reading events (and any downstream reactions) as depicted in Figure 6.

The Reconciliation Program algorithm has been summarized as a flowchart in Figure S1, and its context in the polypeptide elongation model is indicated in Figure 1C of the main text.

3.4 Update of Molecules

Due to the fast turnover of aminoacyl-tRNAs, each tRNA undergoes multiple cycles of aminoacylation during the simulated time step. As a result, net changes to the tRNA pools were reflected when updating the number of molecules at the end of each time step.

To do so, the net number of aminoacylation events Δn that occurred during time step Δt was determined by subtracting the number of aminoacyl-tRNAs at the end of the time step n_1 from that at the beginning of the time step n_0:

$$\Delta n = n_1 - n_0 \tag{56}$$

where $\Delta n > 0$ indicates net aminoacylation and $\Delta n < 0$ indicates net utilization of aminoacyl-tRNAs.

Each net aminoacylation event increments the number of aminoacyl-tRNAs, decrements the number of unaminoacylated tRNAs and amino acids, and converts one molecule of ATP to PPi and AMP.

$$\begin{aligned}
\texttt{aminoacyl-tRNA}_i &\leftarrow \texttt{aminoacyl-tRNA}_i + \Delta n_i \\
\texttt{tRNA}_i &\leftarrow \texttt{tRNA}_i - \Delta n_i \\
\texttt{amino acid}_i &\leftarrow \texttt{amino acid}_i - \Delta n_i \\
\texttt{ATP} &\leftarrow \texttt{ATP} - \Delta n_i \\
\texttt{PPi} &\leftarrow \texttt{PPi} + \Delta n_i \\
\texttt{AMP} &\leftarrow \texttt{AMP} + \Delta n_i
\end{aligned}$$

where i indicates the tRNA isoacceptor type and its corresponding amino acid, and $\Delta n_i > 0$.

Each net aminoacyl-tRNA utilization event indicates an incorporation of an amino acid residue from the aminoacyl-tRNA to the nascent polypeptide and releases a proton molecule. For clarity, the net number of aminoacyl-tRNA utilization events for the i-th aminoacyl-tRNA is described as Δd_i, where $\Delta d_i = -\Delta n_i$.

$$\begin{aligned}
\texttt{aminoacyl-tRNA}_i &\leftarrow \texttt{aminoacyl-tRNA}_i - \Delta d_i \\
\texttt{tRNA}_i &\leftarrow \texttt{tRNA}_i + \Delta d_i \\
\texttt{H}^+ &\leftarrow \texttt{H}^+ + \Delta d_i
\end{aligned}$$

As a result of reflecting net changes to the tRNA pools, a portion of the total number of times the i-th codon is read ($n_{codon,i}$) is represented as aminoacyl-tRNA utilization events (Δd_i) while the remaining portion is represented as direct incorporation of residues ($n_{direct,i}$) from the amino acid pool as in the prior E. coli Model [1].

$$n_{codon,i} = \Delta d_i + n_{direct,i} \tag{57}$$

Each direct incorporation of residue produces a water molecule:

$$\texttt{H}_2\texttt{O} \leftarrow \texttt{H}_2\texttt{O} + n_{direct,i}$$

3.5 N-terminal Cleavage of Initial Methionines

In each time step, the maximal number of cleavage events by MAP ($n_{MAP,max}$) was determined by assuming the maximal reaction rate according to Michaelis-Menten enzyme kinetics (described in Section 1.4 during the time step duration Δt.

$$n_{MAP,max} = v_{MAP,max} \cdot V \cdot N_A \cdot \Delta t \tag{58}$$

where $v_{MAP,max}$ is described in Equation 30, N_A is Avogadro's number, and V is the cell volume.

At each time step, the maximal number of cleavage events by MAP ($n_{MAP,max}$) was compared with the candidate number of cleavage events ($n_{candidates}$) to determine how many cleavage events could occur. MAP substrates that are successfully cleaved proceed to translation termination.

Algorithm 5: N-terminal Cleavage of Initial Methionines

Input : n_{MAP}, Number of MAP molecules in the cell
Input : k_{cat}, Rate of catalysis of MAP, 6 s^{-1} [9]
Input : $n_{candidates}$, number of terminating ribosomes that are synthesizing a MAP substrate (Table 3 lists all documented substrates of MAP.)
Input : V, Cell volume
Input : N_A, Avogadro's number
Input : Δt, Time step

```
/* Determine kinetic capacity of MAP */
```
$v_{MAP,max} = k_{cat} \cdot n_{MAP}$
$n_{MAP,max} = v_{MAP,max} \cdot V \cdot N_A \cdot \Delta t$

```
/* Compare the maximal and candidate numbers of cleavage events */
```
if $n_{candidates} \leq n_{MAP,max}$ **then**
  Proceed all terminating ribosomes to termination.
else
  Determine the number of substrates that cannot be cleaved, $n_{\texttt{cannot cleave}}$.
  $n_{\texttt{cannot cleave}} = n_{candidates} - n_{MAP,max}$
  Randomly select $n_{\texttt{cannot cleave}}$ ribosomes.
  Prevent the $n_{\texttt{cannot cleave}}$ ribosomes from termination in this time step.

Result: The number of successfully terminating ribosomes is limited by the maximal kinetic capacity of MAP.

4 Analysis

4.1 Simulations

Prior to running simulations, the Parameter Calculator (Parca) was run to initialize simulation data, including the parameter optimization program described in Section 2. The command used to run the Parca was:

```
python runscripts/manual/runParca.py --optimize-trna-charging-kinetics sims
```

where `sims` is the name of the simulation output directory, and the `--optimize-trna-charging-kinetics` option enables the parameter optimization program.

The full set of simulations performed in this study was generated from the `trna_synthetase_kinetics` variant located at `wcEcoli/models/ecoli/sim/variants`.

Variant	Model	Perturbation	Simulations		
			# Generations	# Random Seeds	Total # of Viable Cells
0	Updated Model	Optimized aminoacyl-tRNA synthetase kinetics	10	15	150
1	Prior Model	N.A. (unlimited tRNA aminoacylation)	10	10	100
2	Updated Model	Highest measured aminoacyl-tRNA synthetase k_{cat}s	2	50	100
3	Updated Model	HisRS $k_{cat} = 142s^{-1}$ (highest measured value)	10	15	150
4	Updated Model	HisRS $k_{cat} = 183s^{-1}$	10	10	100
5	Updated Model	HisRS $k_{cat} = 223s^{-1}$	10	10	100
6	Updated Model	ArgRS $k_{cat} = 26s^{-1}$ (highest measured value)	20	10	171
7	Updated Model	*argA* codon sequence changed: tandem CGGs at positions 153 and 154 changed to tandem CGUs; ArgRS $k_{cat} = 26s^{-1}$ (highest measured value).	20	3	60
8	Updated Model	*argA* codon sequence changed: tandem CGGs at positions 153 and 154 changed to tandem CGUs.	20	3	60

Table 11: Simulations performed in this study. All simulations represent aerobic growth in M9 Minimal Media supplemented with 0.4% glucose at 37 °C. For single k_{cat} perturbations (Variants 3 through 6), all other aminoacyl-tRNA synthetase kinetic parameters were held at their optimized values.

The commands used to run these simulations were:

```
python runscripts/manual/runSim.py -v trna_synthetase_kinetics 0 0 -g 10 -i 15 sims
python runscripts/manual/runSim.py -v trna_synthetase_kinetics 1 1 -g 10 -i 10 sims
python runscripts/manual/runSim.py -v trna_synthetase_kinetics 2 2 -g 2 -i 50 sims
python runscripts/manual/runSim.py -v trna_synthetase_kinetics 3 3 -g 10 -i 15 sims
python runscripts/manual/runSim.py -v trna_synthetase_kinetics 4 4 -g 10 -i 10 sims
python runscripts/manual/runSim.py -v trna_synthetase_kinetics 5 5 -g 10 -i 10 sims
python runscripts/manual/runSim.py -v trna_synthetase_kinetics 6 6 -g 20 -i 10 sims
python runscripts/manual/runSim.py -v trna_synthetase_kinetics 7 7 -g 20 -i 3 sims
python runscripts/manual/runSim.py -v trna_synthetase_kinetics 8 8 -g 20 -i 3 sims
```

4.2 Methods

Several figure panels investigate comparisons made across different simulations. The table below summarizes which simulations were used to perform the analysis presented in each figure panel.

Figure	Panel	Simulation Variants						
		0	1	2	3	4	5	6
2	A		gray histogram	blue histogram				
	B			(entire panel)				
	C			(entire panel)				
	E		y-axis					
	F		blue histogram					
3	B	blue histogram	gray histogram					
	C	bottom row	top row					
	D	y-axis	x-axis					
	E	y-axis	x-axis					
	F	y-axis						
	G	y-axis						
4	A	gray beeswarm			blue beeswarm			
	B				(entire panel)			
	C				(entire panel)			
	D				(entire panel)			
	E				(entire panel)			
	F	all gray			all blue			
	H	all gray			all blue	all green	all yellow	
	I	1st row			4th row	3rd row	2nd row	
5	A	gray bar						top panel, blue bars
	B	gray lines						blue & green lines
	C	gray bars						blue & green bars
	D							(entire panel)
	E	gray bars						blue bars
	F	gray lines						blue lines
	G	gray lines						blue lines
	H	left panel						right panel
	I	top pie chart						bottom pie chart
6	A	left column						right column

Table 12: Simulations used to perform the analyses presented in each figure of the main text.

4.2.1 Analysis Methods for Panels in Figure 2

- **Panel A**: Doubling times of cells simulated in the Prior Model (Variant 1, gray) and the Updated Model when using measured aminoacyl-tRNA synthetase k_{cat}s (Variant 2, blue) were obtained by measuring the time between cell division events. The anticipated doubling time of 44 minutes was informed [10, 11]. The command that generated the panel was:
`python models/ecoli/analysis/variant/trna_synthetase_kinetics_variant_doubling.py sims`

- **Panel B**: Mass accumulation during the cell cycle in a representative cell simulated in the Updated Model when using measured aminoacyl-tRNA synthetase k_{cat}s was obtained from Variant 2, Seed 11, Generation 0, which was selected for exhibiting a doubling time that is closest to the population average. Protein mass includes all protein monomers and complexes. Non-protein mass includes DNA, RNA, and small molecules. The command that generated the panels was:
`python models/ecoli/analysis/cohort/mass_cell_cycle.py -v 2 sims`

- **Panel C**: Mass accumulation during each cell cycle was obtained from cells simulated in the Updated Model when using measured aminoacyl-tRNA synthetase k_{cat}s (Variant 2). Protein mass includes all protein monomers and complexes. Non-protein mass includes DNA, RNA, and small molecules. The command that generated the panels was:
`python models/ecoli/analysis/cohort/mass_cell_cycle.py -v 2 sims`

- **Panel E**: The simulated proteome was obtained from simulations in the Prior Model (Variant 1, y-axis) by taking the average number of molecules across all time steps, adding 1 (to view molecules with an average of 0 molecules on the log-log plot), and taking the log base 10. The measured proteome was

obtained from [12]. Of the 4,307 proteins in the model, 2,141 were measured in the proteomics dataset and are therefore included in the analysis. The command that generated the panel was:
`python models/ecoli/analysis/cohort/trna_synthetase_average.py -v 1 sims`

- **Panel F**: The simulated distribution of the number of molecules was obtained from simulations in the Prior Model (Variant 1, blue histogram) by recording the molecule abundance at every time step. The measured distribution was obtained from [13], which was performed in slower-growing cells with doubling times of 150 minutes. To be comparable with our faster growing cells (44 minutes doubling time), the measured distributions were scaled up to match the observed mean number of molecules for each aminoacyl-tRNA synthetase while preserving the shape of the measured distributions. The command that generated the panel was:
`python models/ecoli/analysis/cohort/trna_synthetase_distribution.py -v 1 sims`

4.2.2 Analysis Methods for Panels in Figure 3

- **Panel B**: Doubling times of cells simulated in the Prior Model (Variant 1, gray) and the Updated Model when using the optimized aminoacyl-tRNA synthetase kinetic parameters (Variant 0, blue) were obtained by measuring the time between cell division events. The anticipated doubling time of 44 minutes was informed [10, 11]. The command that generated the panel was:
`python models/ecoli/analysis/variant/trna_synthetase_kinetics_variant_doubling.py sims`

- **Panel C**: Aminoacylation rate was calculated by dividing the number of aminoacylation events occurring in each time by the time step duration. tRNA abundances were retrieved from each time step in the simulation and were used to calculate the aminoacylated fraction (number of aminoacylated divided by the total tRNA). tRNA-codon interaction rates were calculated by dividing the number of tRNA-codon interactions occurring in each time step by the time step duration. Polypeptide elongation rate was calculated by dividing the number of Alanine (top) or Alanine codon (bottom) incorporations in each time step by the time step duration. N-terminal methionine cleavage rate was calculated by dividing the number of cleavage events in each time step by the time step duration. The data were obtained from simulations in the Prior Model (Variant 1, top row) and the Updated Model when using the optimized aminoacyl-tRNA synthetase kinetic parameters (Variant 0, bottom row). Cell cycles were aligned to relative time (between 0 and 1), then interpolation was used to sample 1000 points between 0 and 1 for each cell. The command that generated the panel was:
`python models/ecoli/analysis/variant/trna_charging_views.py sims`

- **Panel D**: The proteome abundance was obtained from cell simulated in the Prior Model (Variant 1, x-axis) and the Updated Model when using the optimized aminoacyl-tRNA synthetase kinetic parameters (Variant 0, y-axis) by taking the average number of molecules across all time steps and adding 1 (to view molecules with an average of 0 molecules on the log-log plot). All 4,307 simulated proteins participated in the analysis. The command that generated the panel was:
`python models/ecoli/analysis/variant/trna_charging_validation.py sims`

- **Panel E** The average fluxome was obtained from cells simulated in the Prior Model (Variant 1, x-axis) and the Updated Model when using the optimized aminoacyl-tRNA synthetase kinetic parameters (Variant 0, y-axis) by recording the flux of the reactions of central carbon metabolism reported in Table 13. The command that generated the panel was:
`python models/ecoli/analysis/variant/trna_charging_validation.py sims`

- **Panel F** The simulated intracellular concentrations of tRNA isoacceptors was obtained from simulations in the Updated Model when using the optimized aminoacyl-tRNA synthetase kinetic parameters (Variant 0, y-axis) by taking the average concentration of molecules across all time steps. The measured intracellular concentrations were obtained from [14]. Each tRNA isoacceptor is represented by a gray dot; the mapping between tRNA isoacceptors reported in [14] to the tRNA isoacceptors modeled in our simulations is reported in Table 14. The command that generated the panel was:
`python models/ecoli/analysis/variant/trna_charging_validation.py sims`

- **Panel G**: The distribution of total tRNA was obtained my monitoring the number of all tRNA molecules (as a single sum) across all time steps. The doubling time value (x position of the violin plot) was calculated by taking the average of all simulated doubling times. The molecular abundance of tRNAs across the range of doubling times shown on the x-axis was taken from [15]. The command that generated the panel was:
`python models/ecoli/analysis/variant/trna_charging_validation.py sims`

- **Panel H**: The range of candidate k_{cat} values resulting from the aminoacyl-tRNA synthetase kinetic parameter optimization (blue bars) and the selection of the best candidate (blue dots) are described in Section 2. The range of measured k_{cat} values (gray bars) was observed from Table S1, and the Jakubowski and Goldman-estimated lower limit and the *in vitro* measurement they referenced were obtained from [16]. The ranking of aminoacyl-tRNA synthetases by their agreement with other reports was calculated by subtracting the higher of the highest measurement (top edge of gray bars) or Jakubowski and Goldman's estimation (gray triangles) by the optimized k_{cat} (blue dots). The command that generated the panel was:
`python models/ecoli/analysis/parca/trna_synthetase_kcats.py sims`

Reaction (EcoCyc ID [3])	Enzymes
TRANS-RXN-157	β-glucoside specific PTS enzyme II / BglG kinase / BglG phosphatase: bglF<br>mannose-specific PTS enzyme II: manZ, manY, manX<br>glucose-specific PTS enzyme II: ptsG, crr
PGLUCISOM-RXN	glucose-6-phosphate isomerase (pgi)<br>5-dehydro-4-deoxy-D-glucuronate isomerase (kduI)
6PFRUCTPHOS-RXN	6-phosphofructokinase 1: pfkA<br>6-phosphofructokinase 2: pfkB
F16ALDOLASE-RXN	fructose-bisphosphate aldolase class I: fbaB<br>fructose-bisphosphate aldolase class II: fbaA
TRIOSEPISOMERIZATION-RXN	triose-phosphate isomerase: tpiA
GAPOXNPHOSPHN-RXN	glyceraldehyde-3-phosphate dehydrogenase: gapA
2PGADEHYDRAT-RXN	enolase: eno
PEPDEPHOS-RXN	pyruvate kinase: pykF<br>pyruvate kinase: pykA
PYRUVDEH-RXN	pyruvate dehydrogenase: aceE, aceF, lpd
GLU6PDEHYDROG-RXN	$NADP^+$-dependent glucose-6-phosphate dehydrogenase: zwf
RXN-9952	6-phosphogluconate dehydrogenase, decarboxylating: gnd
RIBULP3EPIM-RXN	ribulose-phosphate 3-epimerase: rpe
RIB5PISOM-RXN	ribose-5-phosphate isomerase A: rpiA<br>allose-6-phosphate isomerase / ribose-5-phosphate isomerase B: rpiB
1TRANSKETO-RXN	transketolase 2: tktB<br>transketolase 1: tktA
TRANSALDOL-RXN	transaldolase A: talA<br>transaldolase B: talB
2TRANSKETO-RXN	transketolase 1: tktA<br>transketolase 2: tktB
CITSYN-RXN	citrate synthase: gltA
ISOCITDEH-RXN	isocitrate dehydrogenase: icd
2OXOGLUTARATEDEH-RXN	2-oxoglutarate dehydrogenase complex: sucA, sucB, lpd
SUCCINATE-DEHYDROGENASE-UBIQUINONE-RXN-SUC/UBIQUINONE-8/FUM/CPD-9956.31	succinate:quinone oxidoreductase: sdhD, sdhC, sdhB, sdhA
FUMHYDR-RXN	fumarase E: fumE<br>fumarase D: fumD<br>fumarase A: fumA<br>fumarase B: fumB<br>fumarase C: fumC
MALATE-DEH-RXN	malate dehydrogenase: mdh<br>malate dehydrogenase (oxaloacetate-decarboxylating): maeA
PEPCARBOX-RXN	phosphoenolpyruvate carboxylase: ppc

Table 13: Reactions of central carbon metabolism analyzed.

Dong et al. 1996 [14]	This Study
Ala1B	alaT, alaU, alaV
Ala2	alaW, alaX
Arg2	argQ, argV, argY, argZ
Arg3	argX
Arg4	argU
Arg5	argW
Asn	asnT, asnU, asnV, asnW
Asp1	aspT, aspU, aspV
Cys	cysT
Gln1	glnU, glnW
Gln2	glnV, glnX
Glu2	gltT, gltU, gltV, gltW
Gly1 + 2	glyU, glyT
Gly3	glyV, glyW, glyX, glyY
His	hisR
Ile1 + 2	ileT, ileU, ileV, ileX, ileY
Leu1	leuP, leuQ, leuT, leuV
Leu2	leuU
Leu3	leuW
Leu4	leuX
Leu5	leuZ
Lys	lysQ, lysT, lysV, lysW, lysY, lysZ
Met f1	metV, metW, metY, metZ
Met f2	metV, metW, metY, metZ
Met m	metT, metU
Phe	pheU, pheV
Pro1	proK
Pro2	proL
Pro3	proM
Ser1	serT
Ser2	serU
Ser3	serV
Ser5	serW, serX
Thr1	thrV
Thr2	thrW
Thr3	thrT
Thr4	thrU
Trp	trpT
Tyr1	tyrT, tyrV
Tyr2	tyrU
Val1	valT, valU, valX, valY, valZ
Val2A	valW
Val2B	valV

Table 14: Mapping of tRNA isoacceptors from the measurements reported by Dong and others [14] to this study.

4.2.3 Analysis Methods for Panels in Figure 4

- **Panel A**: Doubling times of cells simulated in the Updated Model when using the optimized (Variant 0, gray) and measured (Variant 3, blue) HisRS k_{cat}s were obtained by measuring the time between cell division events. Each cell's doubling time was rounded to the nearest minute. The command that generated the panel was:
  `python models/ecoli/analysis/variant/doubling_time_beeswarm.py sims`
- **Panel B**: The doubling times of cells simulated in the Updated Model when using the measured HisRS k_{cat} is from Variant 3, Seed 12, all 10 generations. This lineage was selected for including the cell that exhibited the longest doubling time (Variant 3, Seed 12, Generation 9). The doubling times are presented relative to the average of all cells in Variant 3. After ranking the cells in this lineage from longest to shortest doubling times, the top four slowest-growing cells were highlighted in green. The command that generated the panel was:
  `python models/ecoli/analysis/variant/HisRS_kcat_impact.py sims`
- **Panel C**: The ribosome elongation rates in cells simulated in the Updated Model when using the measured HisRS k_{cat} is from Variant 3, Seed 12, all 10 generations. This lineage was selected for including the cell that exhibited the longest doubling time (Variant 3, Seed 12, Generation 9). Deviations by 40% or more from the expected ribosome elongation rate for the simulated growth condition (17.5 amino

acids per second per ribosome) were highlighted in green. The command that generated the panel was:
`python models/ecoli/analysis/variant/HisRS_kcat_impact.py sims`

- **Panel D**: The rate of hisR tRNA aminoacylation in cells simulated in the Updated Model when using the measured HisRS k_{cat} is from Variant 3, Seed 12, all 10 generations. This lineage was selected for including the cell that exhibited the longest doubling time (Variant 3, Seed 12, Generation 9). Periods of time when the ribosome elongation rate deviated by 40% or more from the expected value for the simulated growth condition (see Panel C) were highlighted in green. The command that generated the panel was:
`python models/ecoli/analysis/variant/HisRS_kcat_impact.py sims`
- **Panel E**: The intracellular abundance of HisRS in cells simulated in the Updated Model when using the measured HisRS k_{cat} is from Variant 3, Seed 12, all 10 generations. This lineage was selected for including the cell that exhibited the longest doubling time (Variant 3, Seed 12, Generation 9). Periods of time when the ribosome elongation rate deviated by 40% or more from the expected value for the simulated growth condition (see Panel C) were highlighted in green. The command that generated the panel was:
`python models/ecoli/analysis/variant/HisRS_kcat_impact.py sims`
- **Panel F**: Ribosome elongation rates and HisRS concentrations were retrieved from each time step in cells simulated in the Updated Model when using the optimized (Variant 0, gray) and measured (Variant 3, blue) HisRS k_{cat}s. Each dot represents the ribosome elongation rate and HisRS concentration at a particular time step. Histogram of HisRS concentrations (top) used a bin size of 0.025 μM. Histogram of the ribosome elongation rate (right) used a bin size of 1 amino acid per second per ribosome. The command that generated the panel was:
`python models/ecoli/analysis/variant/synthetase_ribosome_relation.py sims`
- **Panel H**: Ribosome elongation rates and HisRS concentrations were retrieved from each time step in cells simulated in the Updated Model when using the following four HisRS k_{cat} values: the optimized $k_{cat} = 386s^{-1}$ (Variant 0, gray), the measured $k_{cat} = 142s^{-1}$ (Variant 3, blue), $k_{cat} = 183s^{-1}$ (Variant 4, green), and $k_{cat} = 223s^{-1}$ (Variant 5, yellow). Histogram of HisRS concentrations (top) used a bin size of 0.025 μM. Each dot represents the ribosome elongation rate and HisRS concentration at a particular time step. Histogram of the ribosome elongation rate (right) used a bin size of 1 amino acid per second per ribosome. The command that generated the panel was:
`python models/ecoli/analysis/variant/synthetase_ribosome_relation_sweep.py sims`
- **Panel I**: The sensitivity threshold was calculated by determining the highest HisRS concentration that was associated with a ribosome elongation rate of 17 amino acids per second per ribosome or less, which approximately characterizes where the linear decrease in ribosome elongation rate meets the stable elongation of ribosomes at 17.5 amino acids per second per ribosome. The minimum ribosome rate was determined by identifying the minimum y-value. The portion of time with tRNA aminoacylation limitation was calculated as the portion of time steps displaying a ribosome elongation rate that was less than the minimum observed ribosome elongation rate in the simulations performed in the Updated Model when using the optimized aminoacyl-tRNA synthetase kinetic parameters (gray). All columns were calculated from the simulations shown in Figure 4H of the main text.

4.2.4 Analysis Methods for Panels in Figure 5

- **Panel A**: Top panel: Cells simulated in the Updated Model when using the measured ArgRS k_{cat} (variant 6) were checked for viability. Simulated cells that were considered viable had either doubled their cell mass or reached the 3-hour upper limit of simulation time. Using these criteria, the number of viable simulations at each generation number (0 through 19) were summed. Bottom panel: Doubling times of cells simulated in the Updated Model when using the optimized (Variant 0, gray) and measured (variant 6, blue) ArgRS k_{cat}s were obtained by measuring the time between cell division events. The gray bar is an average of all cells in Variant 0 (regardless of generation number). Each blue bar is an

average of n cells, where n is the number of viable cells at each generation (shown in the top panel). The command that generated the panel was:

```
python models/ecoli/analysis/variant/doubling_time_bars.py sims
```

- **Panel B**: Mass of cellular components in cells simulated in the Updated Model when using the optimized (Variant 0, Seed 8, Generation 0 in gray) and the measured (Variant 6, Seed 8, all 20 generations in blue and green) ArgRS k_{cat}s was retrieved from each time step. Variant 6, Seed 8 was selected as a representative lineage because it was one of the lineages that completed all 20 generations, and Variant 0, Seed 8 was selected because it was the complementary lineage (same random seed initialization) to Variant 0. The generations when mass accumulation first halts (highlighted in green) were identified by calculating the mass fold change at each generation and highlighted the first generation at which the mass fold change was less than 1.1-fold. The command that generated the panel was:
```
python models/ecoli/analysis/variant/mass_single_lineage.py sims
```

- **Panel C**: The mass fold change of cells simulated in the Updated Model when using the optimized (Variant 0, gray) and the measured (Variant 6, blue and green) ArgRS k_{cat}s was obtained by dividing the final mass by the initial mass of each cell cycle for each mass component shown. The gray bar is an average of all cells in Variant 0 (regardless of generation number). Each blue and green bar is an average of n cells, where n is the number of viable cells at each generation (shown in Panel A, top). The generations when mass accumulation first halts (highlighted in green) are the first generations when the median mass fold change is less than or equal to 1.1-fold. The command that generated the panel was:
```
python models/ecoli/analysis/variant/mass_cell_cycle_fold_change.py sims
```

- **Panel D**: The tRNA aminoacylated percent was calculate from cells simulated in the Updated Model when using the measured ArgRS k_{cat} (Variant 6, all viable $n = 171$ cells, blue). After identifying the division event that first leads to cessation of protein mass accumulation (such as the green-highlighted generation 9 in Figure 5B of the main text), the following calculation was performed using only the generations before the identified division events: For each amino acid, the intracellular abundance of all tRNA isoacceptors cognate to the given amino acid were summed (in number of molecules), and the aminoacylated percent was calculated (as the number of aminoacyl-tRNAs divided by the total number of tRNAs) at each time step. The violin plots show the distributions of aminoacylated percent across all time steps. The command that generated the panel was:
```
python models/ecoli/analysis/cohort/trna_charged_fractions.py -v 6 sims
```

- **Panel E**: The similarity of the intracellular abundances of ArgRS, arginine, arginine tRNAs, and ATP between adjacent cell cycles was calculated as a fold change of average concentrations (mean concentration in a daughter cell divided by the mean concentration in its mother cell). For cells simulated in the Updated Model using the measured ArgRS k_{cat} (Variant 6, blue): for each lineage, only the two cell cycles immediately preceding and following the first division event that leads to cessation of protein mass accumulation were included. For cells simulated in the Updated Model using the optimized ArgRS k_{cat} (variant 0, gray), which did not exhibit any protein cessation events and therefore all cell cycles could serve as examples of typical growth, all cell cycles were included. The command that generated the panel was:
```
python models/ecoli/analysis/variant/argRS_and_substrates.py sims
```

- **Panel F**: Intracellular concentrations (glutamate, N-acetylglutamate synthase, citrulline, and arginine) and reaction fluxes (N-acetyl transfer reaction) were retrieved from cells simulated in the Updated Model when using the optimized (Variant 0, Seed 8, all 10 Generations in gray) and measured (Variant 6, Seed 8, all 20 Generations in blue) ArgRS k_{cat}s. Seed 8 was selected as a representative lineage from Figure 5B of the main text. Data corresponding to the measured ArgRS k_{cat} (blue) show the moving average calculated using a 10-minute window. In order to facilitate comparisons with the optimized ArgRS k_{cat} simulations (which simulated 10 generations instead of 20), the data corresponding to the optimized ArgRS k_{cat} (gray) show the median value calculated from all 10 generations of the lineage. The period of arginine depletion (yellow line) was identified as the period of time when the intracellular arginine concentration in the measured ArgRS k_{cat} simulations (Variant 6) deviated from the median

value observed from the optimized ArgRS k_{cat} simulations (Variant 0) by more than 30 μM. The period of no flux through the N-acetyl transfer reaction (red line) was identified as the period of time when the reaction flux was 0. The command that generated the panel was:
`python models/ecoli/analysis/variant/arginine_biosynthesis.py sims`

- **Panel G**: Expression of ArgA in cells simulated in the Updated Model when using the optimized (Variant 0, Seed 8, Generation 0 in gray) and measured (Variant 0, Seed 8, all 20 Generations) ArgRS k_{cat}s were calculated as the number of events observed per 2-second time step (for monomer synthesis, monomer degradation, and complexation) or the number of molecules at each time step (for monomer and hexamer abundances). Seed 8 was selected as a representative lineage from Figure 5B of the main text. The period of ArgA hexamer absence (red line) was identified as the period of time when the hexamer molecular abundance was at 0. The command that generated the panel was:
`python models/ecoli/analysis/variant/argA_counts.py sims`

- **Panel H**: Simulated ribosome profiling experiment from cells simulated in the Updated Model when using the optimized (Variant 0, left panel) and measured (Variant 6, right panel) ArgRS k_{cat}s was obtained by monitoring the position of (and codon identity at) the unoccupied A site of ribosomes processing on *argA* mRNAs by the end of each time step. Ribosome positions associated with each pair of consecutive time steps was compared to compute n, the number of codons processed by each ribosome during each time step. By assuming that ribosomes spent an equal amount of time on each of the n codons, the time spent on each codon (t) during the time step τ could be calculated as $t = \tau/n$. Then, the total amount of time spent on each codon position during all simulations was determined by summing all t calculated from all pairs of consecutive time steps. Finally, the fraction of time spent on each codon was determined by normalizing the total time spent on each codon position. The command that generated the panel was:
`python models/ecoli/analysis/variant/argA_ribosome_profile.py sims`

- **Panel I**: Premature ribosome terminations in cells simulated in the Updated Model when using the optimized (Variant 0, top pie chart) and measured (Variant 6, bottom pie chart) ArgRS k_{cat}s were obtained by retrieving the total number of ribosomes that had initiated on *argA* mRNA transcripts and, of those ribosomes, the number that had terminated prematurely. Of the prematurely terminated ribosomes, the identity of the codon in the open A site of the ribosomes were retrieved to determine the portion of all premature terminations that occurred while the ribosome was waiting on an arginine codon (and compared to the number that prematurely terminated while waiting on non-arginine codons). The command that generated the panel was:
`python models/ecoli/analysis/variant/argA_ribosome_terminations.py sims`

4.2.5 Analysis Methods for Panels in Figure 6

- **Panel A**: All data were obtained from cells simulated in the Updated Model when using the optimized (Variant 0, Seed 8, all 10 Generations on the left column) and measured (Variant 6, Seed 8, all 20 Generations on the right column) ArgRS k_{cat}s. Seed 8 was selected as a representative lineage from Figure 5B of the main text. From top to bottom:
  - Row 1: The maximal aminoacylation rate was calculated as $k_{cat} \cdot [ArgRS]$ at each time step.
  - Row 2: The aminoacylated fraction was calculated by dividing the total number of arginine-charged arginine tRNAs by the total number of arginine tRNAs (both aminoacylated and not).
  - Row 3: The ribosome A site position along *argA* mRNA transcripts was monitored during simulations. The codon sequence is numbered from 0 (start codon) to 442 (the final sense codon). Codon identities are colored: CGU in blue, CGG in green, CGC in yellow, AGG in pink, and non-arginine codons in gray).
  - Row 4: Ribosome accumulation on *argA* mRNA transcripts was calculated by dividing the number of active ribosomes on *argA* mRNAs by the number of *argA* mRNA transcripts. Note: volume

exclusion of polysomes on shared transcripts is not yet represented in this model. For reference, our observation of up to 40 ribosomes per *argA* transcript in the measured ArgRS k_{cat} simulation (right column) agrees with the length of the transcript (443 amino acids, or 1332 base pairs including the stop codon), which suggests that a maximum of 55 ribosomes would physically fit (when assuming an estimated ribosome footprint of 24 nucleotides [17]).

- Row 5: The premature ribosome termination rate was calculated by dividing the number of premature ribosome termination events from *argA* mRNAs in each time step by the time step length (2 seconds).
- Row 6: The number of ArgA monomers were monitored at each time step and were directly shown.
- Row 7: The number of ArgA hexamer (acetylglutamate synthase) were monitored at each time step and were directly shown. The period of time when the ArgA hexamer abundance was at 0 molecules was indicated by the red line.
- Row 8: Flux through the N-acetyl transfer reaction in the arginine biosynthesis pathway is shown as the moving average using 10-minute windows. The period of time when the N-acetyl transfer reaction had a flux of 0 mmol/gDCW/h was indicated by the red line.
- Row 9: The intracellular concentration of arginine was monitored at each time step and were directly shown.
- Row 10: The fractional saturation of ArgRS for its substrate arginine was calculated as $[A]/(K_{M,A}+[A])$, where $[A]$ is the concentration of arginine and $K_{M,A}$ is the Michaelis-Menten constant describing the affinity between arginine and ArgRS.
- Row 11: The aminoacylation rate of arginine tRNAs was calculated according to Equation 4. The period of time when the aminoacylation rate had a value of 0 μM$/s$ was indicated by the red line.
- Row 12: The global ribosome elongation rate was calculated as the total number of elongation steps completed by all active ribosomes during a time step, divided by the time step (2 seconds), divided by the number of active ribosomes during that time step. The period of time when the ribosome elongation rate had a value of 0 amino acids/s/ribosome was indicated by the red line.
- Row 13: Total protein mass was monitored at each time step of the simulations and was directly shown.

The command that generated the panel was:
`python models/ecoli/analysis/variant/argRS_kcat_impact.py sims`

4.2.6 Analysis Methods for Panels in Supporting Figure S1

The flowchart in Figure S1 depicts the algorithm described in Section 3.3, which has been developed in the following file:
`wholecell/utils/_trna_charging.pyx`.

4.2.7 Analysis Methods for Panels in Supporting Figure S2

For Figure S2, please refer to the description of Figure 2F in Section 4.2.1.

4.2.8 Analysis Methods for Panels in Supporting Figure S3

For Figure S3, the concentrations of aminoacyl-tRNA synthetases were observed in sample runs from the previous model that were generated by the commands:

```
python runscripts/manual/runSim.py --no-trna-charging --translation-supply -v condition 0 0 -g 20 -i 5 out
python runscripts/manual/runSim.py --no-trna-charging --translation-supply -v condition 2 2 -g 20 -i 5 out
```
where `out` is the name of the simulation output directory.

Then, for each aminoacyl-tRNA synthetase, the distance between the minimum observed concentration and 0 μM was divided into five equal parts, of which the values corresponding to the four highest values (labeled '2' through '5') were used as the minimum aminoacyl-tRNA synthetase concentration described in Section 2.3.2. The command that generated the panel was:
`python models/ecoli/analysis/variant/trna_synthetase_dynamic_range_sweep.py out`

4.2.9 Analysis Methods for Panels in Supporting Figure S4

- **Panel A**: The abundances of tRNAs were retrieved from each time step of the simulations in the Updated Model when using the optimized aminoacyl-tRNA synthetase kinetic parameters (Variant 0). The violin plots show the distributions of the number of molecules per cell across all time steps. The command that generated the panel was:
  `python models/ecoli/analysis/cohort/trna_forms.py -v 0 sims`
- **Panel B**: The simulated tRNA aminoacylation percentages were calculated at each time step by dividing the number of aminoacyl-tRNAs by the total number of tRNAs for each tRNA type. The experimentally measured aminoacylation percentages and their errors were taken directly from their published reports ([18, 19, 20, 21]. The command that generated the panel was:
  `python models/ecoli/analysis/cohort/trna_charged_fractions_validation.py -v 0 sims`

4.2.10 Analysis Methods for Panels in Supporting Figure S5

- **Panel A**: The percentage of ribosome terminations occurring at each codon position along the *argA* mRNA was calculated by dividing the number of premature ribosome terminations occurring at each codon position by the total number of premature ribosome terminations from the *argA* mRNA (then multiplying 100%). The command that generated the panel was:
  `python models/ecoli/analysis/cohort/premature_ribosome_termination.py -v 6 sims`
- **Panel B**: The locations of arginine codons were identified from the known argA codon sequence [3]. The command that generated the panel was:
  `python models/ecoli/analysis/cohort/premature_ribosome_termination.py -v 6 sims`
- **Panel C**: Proteins with tandem CGGs were identified by searching for two CGG codons in-a-row in their codon sequences; the same criteria were used to identify proteins with tandem AGAs and AGGs. Proteins with no arginine codons were identified as proteins that do not use any of the six arginine codons (AGA, AGG, CGA, CGC, CGG, and CGU). The remaining 4,094 proteins in the proteome were classified as the group of proteins that contain arginine codons that are not tandem arrangements of CGGs, AGAs, or AGGs. The command that determined the number of proteins in each classification was:
  `python models/ecoli/analysis/variant/rare_arginine_codons_expression.py sims`
- **Panel D**: The groups of protein features investigated in this panel are composed of the same protein categories identified in panel C. For each protein, the fold change of its simulated expression was calculated as 1 + the mean number of molecules per cell in the measured ArgRS simulations divided by 1 + the mean number of molecules per cell in the optimized ArgRS simulations. The "No Arginines" group was taken as the reference distribution and described using its mean and standard deviation. For the statistical significance of the difference between the median of each distribution to the "No Arginines" group, the two-tailed p-value was calculated from the z-score of the median. To focus on the time when premature ribosome terminations were occurring (as read from Fig. 6A, row 3), generations

8 through 11 were used in this analysis (total of n = 30 cells in the optimized ArgRS k_{cat} and n = 40 cells in the measured ArgRS k_{cat}). The command that generated the panel was:

```
python models/ecoli/analysis/variant/rare_arginine_codons_expression.py sims
```

- **Panel E**: For each perturbation experiment, the number of molecules per cell was recorded from each time step of the simulations and the distributions are shown. The "CGG" simulations were taken as the reference distributions and described using their means and standard deviations. For the statistical significance of the difference between the means of each "CGU" distribution to its corresponding "CGG" distribution, the two-tailed p-value was calculated from the z-score of the median. For the "Measured ArgRS k_{cat}, CGG" group: to focus on the time when premature ribosome terminations were occurring (as read from Fig. 6A, row 3), generations 8 through 11 were used in this analysis (total of n = 40 cells). In terms of the number of time steps, the number of samples per distribution are: Optimized ArgRS k_{cat}, CGG n = 201,432; Optimized ArgRS k_{cat}, CGU n = 81,264; Measured ArgRS k_{cat}, CGG n = 182,863; Measured ArgRS k_{cat}, CGU n = 245,554. The command that generated the panel was:

```
python models/ecoli/analysis/variant/argA_codon_experiment.py sims
```

5 Supporting Figures and Tables

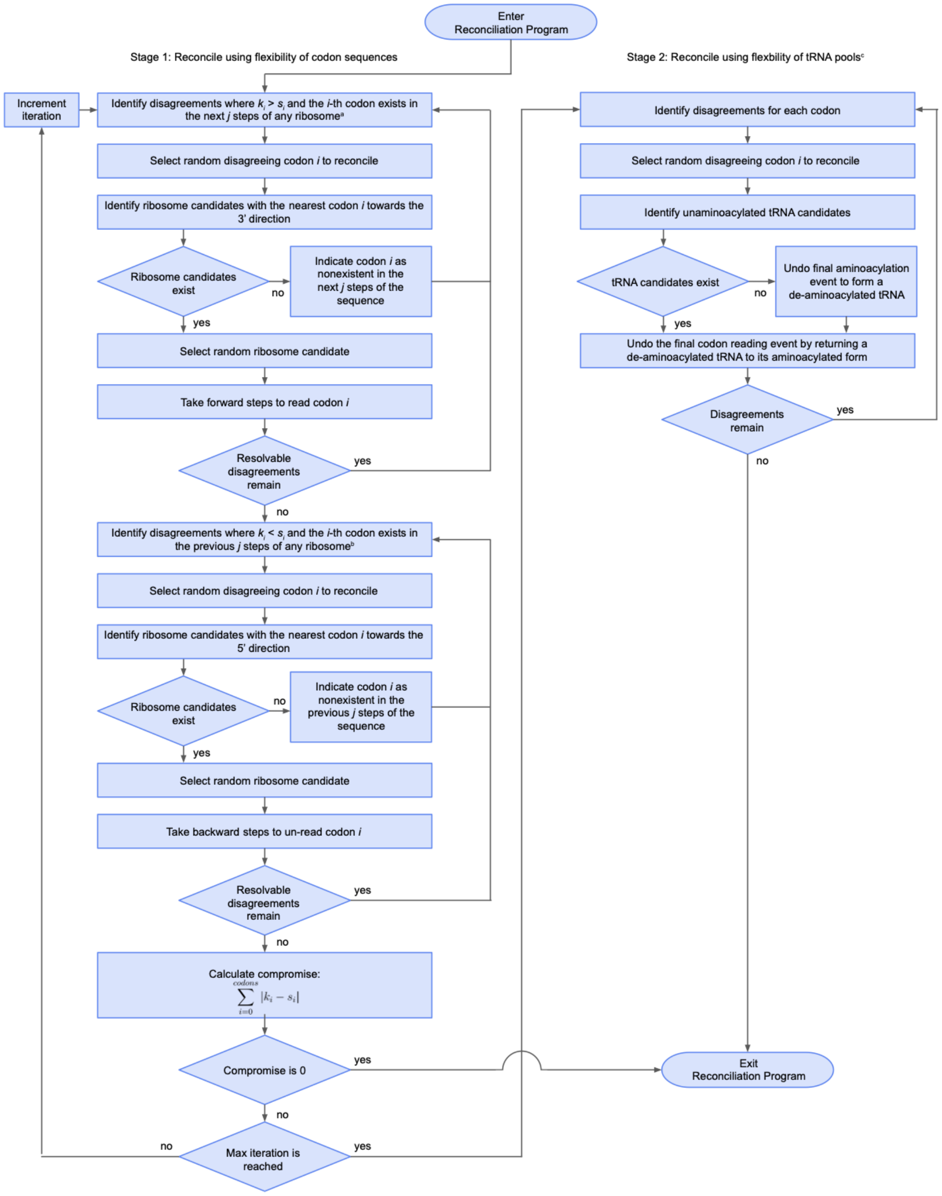

Figure S1. Reconciliation Program. The algorithm used in the Reconciliation Program is described in Section 3.3. Notes: a: Identifies sequence-based underestimations by processing ribosomes forwards toward the 5' end. b: Identifies sequence-based overestimations by processing ribosomes backwards toward the 3' end. c: Disagreements remaining in Stage 2 are sequence-based underestimations. The Reconciliation Program is shown in context to the polypeptide elongation model in Figure 1 of the main text.

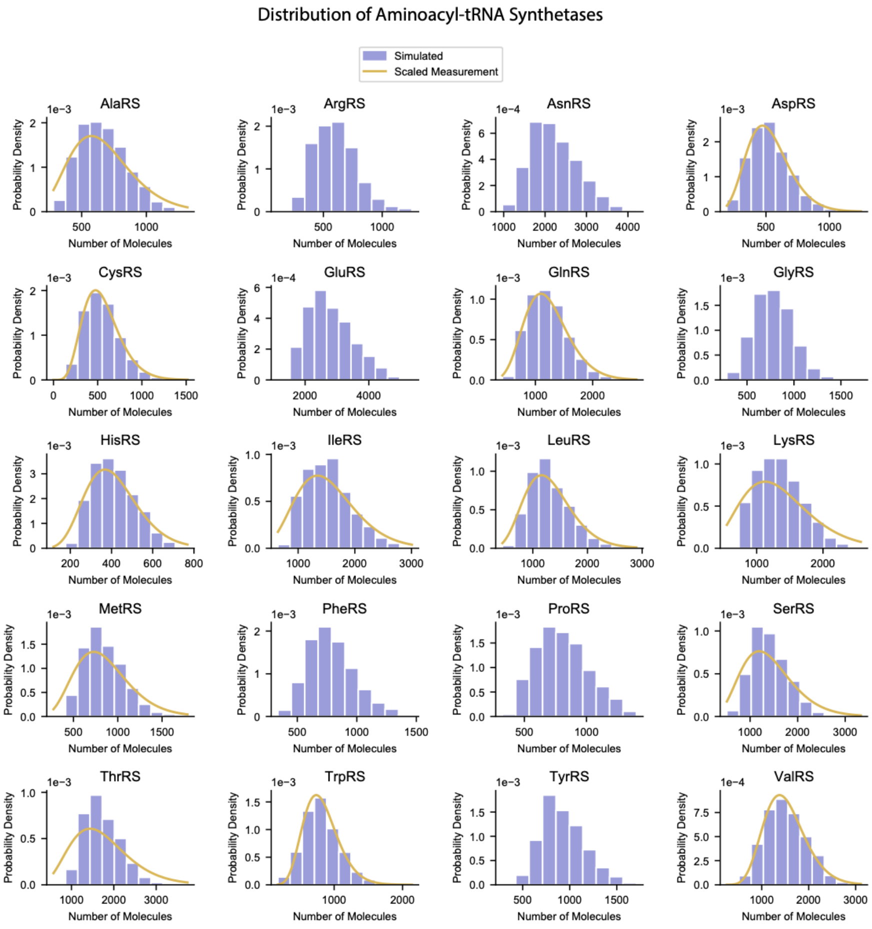

Figure S2. Comparison of distribution of aminoacyl-tRNA synthetase abundances. Distributions of protein abundances estimated from simulations of the Prior Model (n=100 cells, blue) and measured from [13] (yellow) are shown as probability densities. Cells were simulated in aerobic growth in M9 Minimal Media supplemented with 0.4% glucose at 37 °C. Simulations of the Prior Model represent 10-generation long lineages initialized at 10 random seeds (total of n=100 cells). CysRS, AlaRS, and ThrRS are highlighted in Figure 2F of the main text.

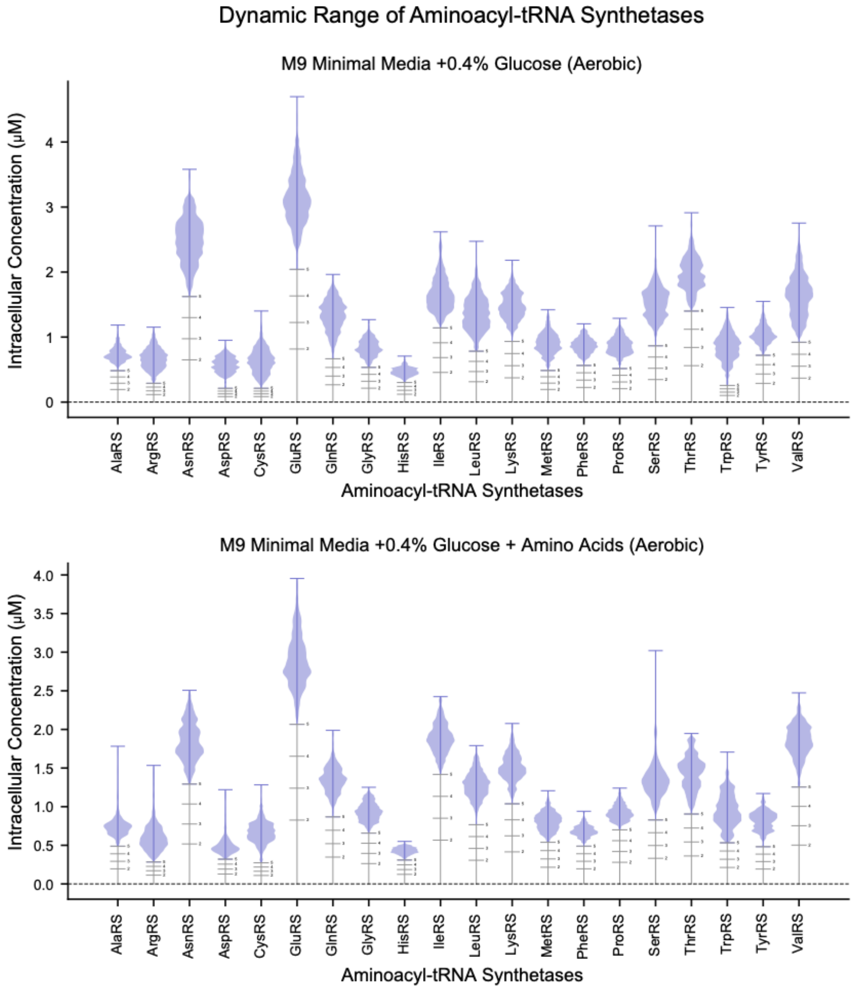

Figure S3. Dynamic range of intracellular aminoacyl-tRNA synthetase concentrations. Range of aminoacyl-tRNA synthetase concentrations observed from simulations run in the Prior Model in nutritionally poor (top) and rich (bottom) media. The tick marks labeled '2' through '5' (reported in Table 9) indicate the values of the minimum aminoacyl-tRNA synthetase concentration $[E_{min}]$ used in Equation 49. Cells were simulated in aerobic growth in M9 Minimal Media supplemented with 0.4% glucose at 37 °C (top) and supplemented with amino acids (5x Supplement EZ without VA Vitamin Solution, bottom). In each media condition, 20-generation long lineages initialized at 5 random seeds (total n=100 cells) were simulated.

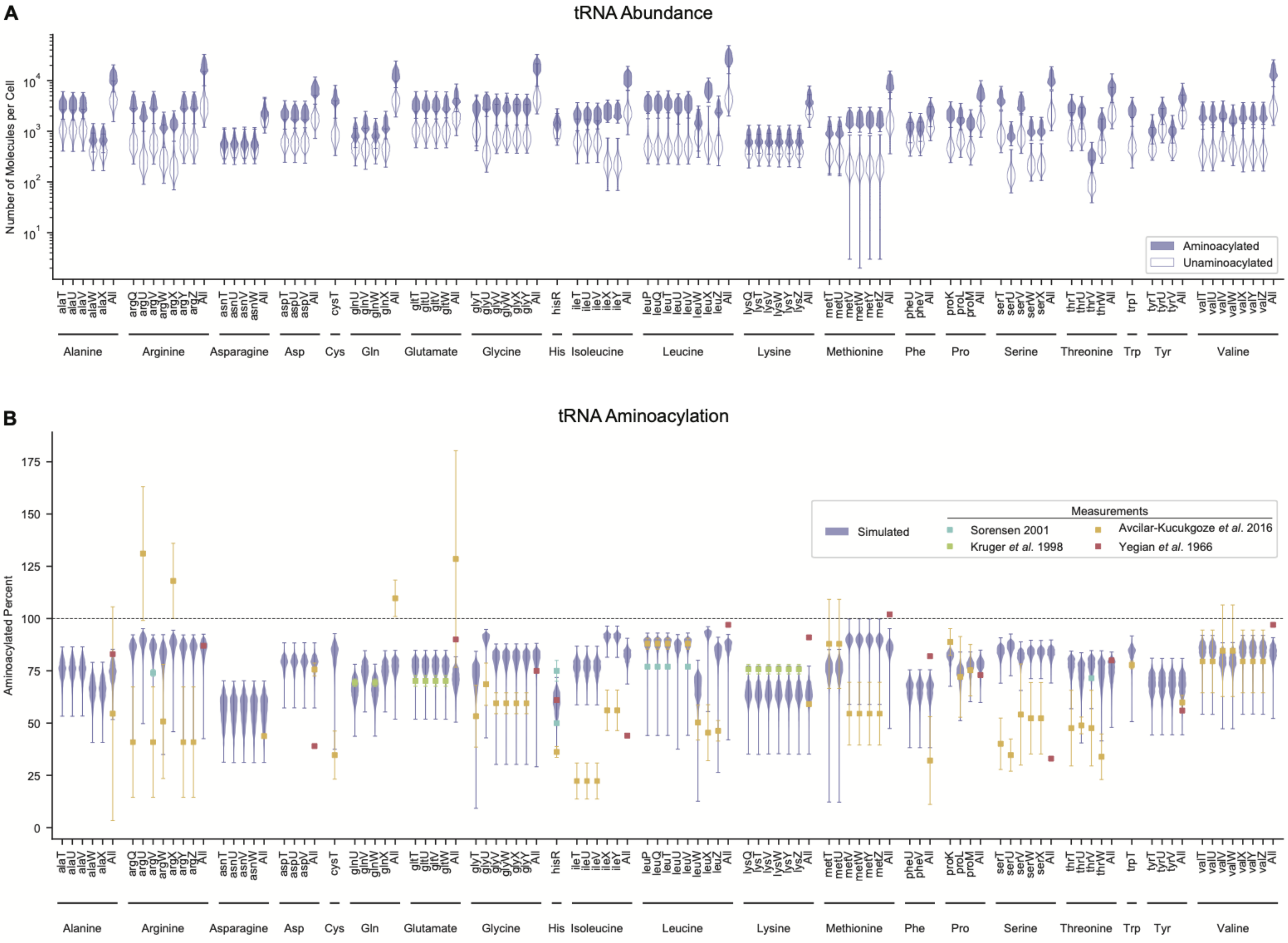
A
tRNA Abundance
Number of Molecules per Cell
Aminoacylated
Unaminoacylated
B
tRNA Aminoacylation
Aminoacylated Percent
Simulated
Measurements
Sorensen 2001
Kruger et al. 1998
Avcilar-Kucukgoze et al. 2016
Yegian et al. 1966
Alanine
Arginine
Asparagine
Asp
Cys
Gln
Glutamate
Glycine
His
Isoleucine
Leucine
Lysine
Methionine
Phe
Pro
Serine
Threonine
Trp
Tyr
Valine

Figure S4. Abundance and aminoacylation of tRNAs. (A) Range of tRNA molecular abundances are shown as the number of tRNA molecules per cell for each tRNA isoacceptor (both aminoacylated and unaminoacylated forms). Error bars indicate the range of observed values. (B) Simulated (violin plots) tRNA aminoacylation percentages are shown in comparison to four studies reporting experimental measurements: Sorensen 2001 [18] (blue), Kruger *et al.* 1998 [19] (green), Avcilar-Kucukgoze *et al.* 2016 [20] (yellow), and Yegian *et al.* 1966 [21] (red). Error bars on the violin plots indicate the range of simulated values while error bars on the measurements represent the standard error of the mean (SEM) [18, 19] or the standard deviation [20]. All simulations in this panel were performed in the Updated Model when using the optimized aminoacyl-tRNA synthetase kinetic parameters. All cells were simulated in aerobic growth in M9 Minimal Media supplemented with 0.4% glucose at 37 °C. Simulations in this figure represent 10-generation long lineages initialized at 15 random seeds (n=150 cells).

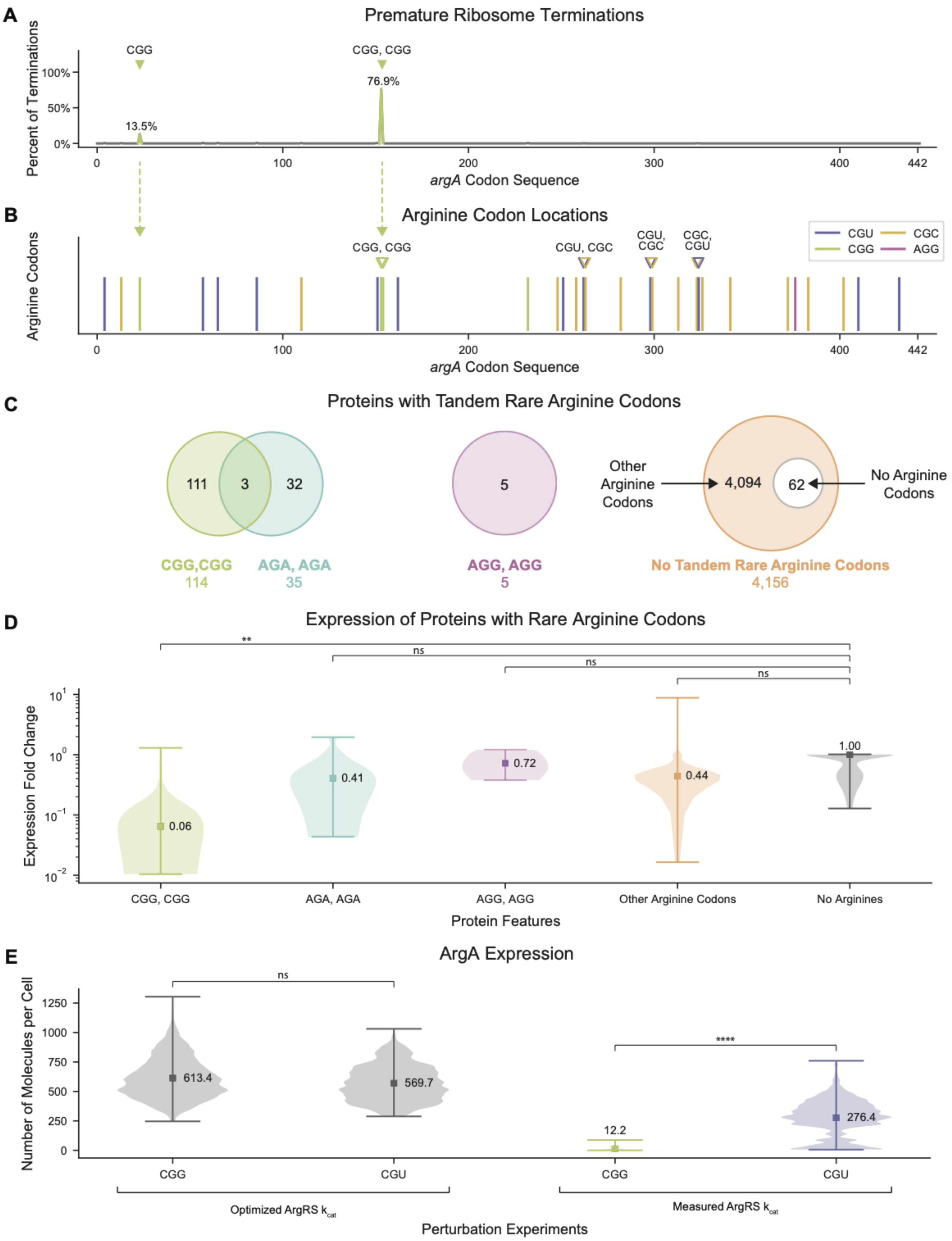
A
Premature Ribosome Terminations
Percent of Terminations
CGG
CGG, CGG
76.9%
13.5%
argA Codon Sequence
B
Arginine Codon Locations
Arginine Codons
CGG, CGG
CGU, CGC
CGU, CGC
CGC, CGU
CGU
CGC
CGG
AGG
C
Proteins with Tandem Rare Arginine Codons
111
3
32
5
Other Arginine Codons
4,094
62
No Arginine Codons
CGG,CGG 114
AGA, AGA 35
AGG, AGG 5
No Tandem Rare Arginine Codons 4,156
D
Expression of Proteins with Rare Arginine Codons
Expression Fold Change
0.06
0.41
0.72
0.44
1.00
CGG, CGG
AGA, AGA
AGG, AGG
Other Arginine Codons
No Arginines
Protein Features
E
ArgA Expression
Number of Molecules per Cell
613.4
569.7
12.2
276.4
CGG
CGU
CGG
CGU
Optimized ArgRS k_{cat}
Measured ArgRS k_{cat}
Perturbation Experiments

Figure S5. Rare arginine codons in the argA mRNA sequence and across the E. coli proteome. (A) Percentage of premature ribosome terminations occurring at each codon position along the *argA* mRNA. The highest (tandem CGG codons at positions 153 and 154) and second highest (CGG codon at position 23) sites of premature ribosome termination are indicated by the inverted triangles and labeled with their percentages and codons. (B) Location of arginine codons in the *argA* mRNA sequence are indicated by the vertical lines. Colors indicate the identity of the arginine codons. Tandem arginine codons are indicated by the inverted triangles and are labeled. (C) Proteins with tandem rare arginine codons. Of the 4,307 proteins in our simulated proteome: 114 have tandem CGGs in their codon sequences, 35 have tandem AGAs, 5 have tandem AGGs, 62 proteins do not have arginine codons, and the remaining 4,094 proteins do not have tandem CGGs, AGAs, nor AGGs. Three proteins (*lhr*, *ymfK*, *recF*) have both tandem CGGs and AGAs in their codon sequences. (D) Distribution of fold changes in expression of proteins with rare arginine codons in their codon sequences. The groups of protein features investigated are composed of the same protein categories identified in panel C. For each protein, the fold change of its expression was calculated as a ratio between the expression in the measured ArgRS simulations to the optimized ArgRS simulations. Statistical significance of the difference between the median of each distribution to the "No Arginines" group (which was taken as the reference distribution) was determined from the two-tailed p-value, which was calculated from the z-score of the median: "CGG, CGG": z-score for median (0.06) is -2.83, p-value is 4.7×10^{-3}; "AGA, AGA": z-score for median (0.41) is -1.55, p-value is 1.2×10^{-1}; "AGG, AGG": z-score for median (0.72) is -0.37, p-value is 7.1×10^{-1}; and "Other Arginine Codons": z-score for median (0.44) is -1.42, p-value is 1.6×10^{-1}. Error bars indicate the range of observed values, and the squares (and their labels) indicate the median. (E) Distribution of the number of ArgA molecules per cell when using the optimized (gray) or measured (blue) ArgRS k_{cat}, and when using the original CGG codons at positions 153 and 154 or using CGU at those positions. Statistical significance of the difference between the mean of each distribution to the "CGG" simulations (which was taken as the reference distribution) was determined from the two-tailed p-value, which was calculated from the z-score of the mean: "Optimized ArgRS k_{cat}": z-score for mean (569.7) is -0.25, p-value is 0.80; and "Measured ArgRS k_{cat}": z-score for mean (276.4) is 15.22, p-value is 2.6×10^{-52}. Error bars indicate the range of observed values, and the squares (and their labels) indicate the mean.

Table S1. Curation of aminoacyl-tRNA synthetase kinetic measurements. The curation consists of 131 k_{cat} measurements reported from 81 studies collectively representing all 20 aminoacyl-tRNA synthetases. For each aminoacyl-tRNA synthetase, the highest measurement taken from studies performed at 37 °C were used in simulations that were analyzed in Figure 2A, 2B, and 2C of the main text. Note: [1] Purchased from Sigma (best guess from catalog: in vivo E. coli).

tRNA Synthetase	Measurements							Used in Figures 2A, 2B, 2C
	k_{cat} (s^{-1})	K_M (μM)			Reference	Temperature (°C)	tRNA Source	
		tRNA	Amino Acid	ATP				
AlaRS	84		340	83	[22]	37	N/A	Yes
	1.1	1.1			[22]	37		
	99		300	140	[23]	not reported		
	1.5	2.4			[23]	not reported		
	0.2	1.5			[24]	37		
	0.44	1.1			[25]	37		
	2				[26]	37		
	1.2		50		[27]	22		
ArgRS	3.3				[28]	37		
	26	2.5	12	900	[29]	37	in vivo E. coli	Yes
	17	1			[30]	37		
	21.9	1.9			[31]	37		
AsnRS	1.6		32		[32]	37	in vivo E. coli	Yes
	1.3			76	[32]	37		
	1.6		32.3		[33]	37	in vivo E. coli	Yes
	1.3			76	[33]	37		
AspRS	18	0.6	60	90	[34]	37		
	2.1	1.7			[35]	37		
	0.55	0.08			[36]	28		
	6.8	0.2	22		[37]	not reported		
	12	0.5		91	[38]	37		
	30		1300		[38]	37	N/A	Yes
CysRS	3.4	0.3			[39]	37		
	0.9	0.35			[40]	37		
	91			290	[40]	37	N/A	Yes
	79		22		[40]	37		
	2.9	0.64			[41]	30		
	4.4			338	[41]	30		
	4.8		7.2		[41]	30		
	2.46	1.16			[42]	37		
	2.46	1.16			[43]	37		
	2.9		22		[26]	37		
	2.5	1.2			[26]	37		
GluRS	3.4	0.08	100	180	[44]	37		
	0.8	0.1	100	200	[45]	37		
	4				[28]	37	in vivo E. coli	Yes
	0.3	0.3	5	40	[46]	30		
	3	56			[35]	37		
GlnRS	0.12				[47]	37		
	3.3	0.2	150	210	[48]	37		
	3.83	0.27			[49]	37	in vivo E. coli	Yes
	2.8		214		[50]	37		
	2.62		114		[51]	37		
	2.8			110	[51]	37		
	3.2		260		[52]	ambient		
GlyRS	36.7	0.2	160	42	[53]	37	in vivo E. coli	Yes
HisRS	2.6	1.4			[54]	37		
	142		30		[54]	37	N/A	Yes
	120			890	[54]	37		
	7		8		[55]	37		
	34			560	[55]	37		
	2.04	0.34			[56]	25		
	2.02	0.34			[57]	37		
	130		30	890	[57]	37		
	1.71	0.34			[58]	30		

Table S1 - continued.

tRNA Synthetase	Measurements							Used in Figures 2A, 2B, 2C
	k_{cat} (s^{-1})	K_M (μM)			Reference	Temperature (°C)	tRNA Source	
		tRNA	Amino Acid	ATP				
	130		30	890	[58]	30		
IleRS	1.46		5.69		[59]	25		
	1.37			30	[59]	25		
	0.3				[28]	25		
	3.1	2.1			[60]	37		
	104		1.3		[60]	37	N/A	Yes
	27.1		5.2	540	[61]	25		
	3.1	2.1			[62]	37		
	104		1.3	700	[62]	37	N/A	Yes
	80.4		3.6	280	[63]	37		
LeuRS	1				[28]	37		
	3.4		15		[64]	not reported		
	4.2			260	[64]	not reported		
	3.9	1.6			[64]	not reported		
	3		15		[65]	37		
	3.6			280	[65]	37		
	2.9	1.5			[65]	37		
	5.2		15		[66]	37		
	5.0			280	[66]	37		
	5.1	2.2			[66]	37		
	5.1		15		[67]	37		
	4.9			260	[67]	37		
	5.0	2.5			[67]	37		
	6.1	1.3	1.5		[68]	not reported		
	5.1	2.6	20		[69]	37		
	5.0			240	[69]	37		
	11		15		[70]	37		
	0.12	0.7			[71]	not reported		
	29	13			[72]	37	N/A	Yes
	9.6	0.73			[73]	not reported		
LysRS	2.5				[28]	37		
	0.05533	1.9			[74]	37		
	6.1		2.7		[75]	37	(in vivo E. coli)[1]	Yes
	3.6	3.4			[36]	28		
	3.4			12	[76]	37		
	1.8		2.6		[76]	37		
MetRS	3.3	0.9			[77]	not reported		
	6.85	1			[78]	37	(in vivo E. coli)[1]	Yes
	2.8	4.4			[79]	25		
	1	3.9			[80]	25		
	5.7		40	350	[80]	25		
	5.6		7900		[81]	37		
	13.3		24.3		[82]	25		
PheRS	1.7				[28]	37		
	138			40	[83]	not reported		
	5.6	7			[83]	not reported		
	3.52		20.2		[84]	37		
	1.72		10.3		[85]	not reported		
	0.817	2.7			[86]	37		
	3.33		5.3		[86]	37		
	199		2		[87]	37	N/A	Yes
ProRS	70		250		[88]	37	N/A	Yes
	14		290		[89]	37		
	0.239	14.14			[90]	not reported		
SerRS	0.34		64		[91]	37		
	0.35			0.068	[91]	37		
	2.6	0.56			[91]	37	in vivo E. coli	Yes
ThrRS	0.2				[28]	30		
	0.53	0.05			[92]	37		
	36		110		[93]	37		
	90		201	267	[94]	37	N/A	Yes
	33		110		[95]	37		
	0.05	1.5			[96]	37		

Table S1 - continued.

tRNA Synthetase	Measurements							Used in Figures 2A, 2B, 2C
	k_{cat} (s^{-1})	K_M (μM)			Reference	Temperature (°C)	tRNA Source	
		tRNA	Amino Acid	ATP				
	13			90	[96]	37		
TrpRS	1.5			190	[97]	37	in vivo E. coli	Yes
	2	0.34	12.4		[97]	37		
	4.8	3.9			[50]	37		
	150.6		16.81		[98]	not reported		
	13.9	0.62			[98]	not reported		
TyrRS	0.74		3.3		[99]	30	N/A	Yes
	12		5.3		[100]	37		
ValRS	6				[28]	25	in vitro transcribed	Yes
	1.5	0.1			[101]	25		
	50		70	110	[101]	25		
	13.9		4.3		[102]	37		
	4				[26]	37		

Table S1 - continued.

Table S2. Optimized aminoacyl-tRNA synthetase kinetic parameters. For each aminoacyl-tRNA synthetase, the optimization approach described in Section 2 generated several candidate solutions, of which the one corresponding to the minimum objective value (reported here) was taken to be the best solution and used for the rest of the study. These optimized parameters are compared to the estimated lower limit of aminoacyl-tRNA synthetase activity reported in [16].

tRNA Synthetase	Optimized Parameters				Estimated [16] Lower Limit of k_{cat} (s^{-1})	Fold Change Above Estimated Lower Limit of k_{cat}
	k_{cat} (s^{-1})	$K_{M,A}$ (μM)	$K_{M,T}$ (μM)			
AlaRS	476.057	1242.769	alaT	21.917		
			alaU	21.917		
			alaV	21.917		
			alaW	3.976		
			alaX	3.976		
ArgRS	210.989	199.182	argQ	7.522	9.7	21.8
			argU	57.372		
			argV	7.522		
			argW	558.475		
			argX	26.311		
			argY	7.522		
			argZ	7.522		
AsnRS	140.324	865.826	asnT	10.378		
			asnU	10.378		
			asnV	10.378		
			asnW	10.378		
AspRS	579.774	2130.913	aspT	12.877		
			aspU	12.877		
			aspV	12.877		
CysRS	69.447	6.282	cysT	10.591		
GluRS	59.131	37449.661	gltT	7.839	2	29.6
			gltU	7.839		
			gltV	7.839		
			gltW	7.839		
GlnRS	208.545	5891.203	glnU	11.789	4.9	42.6
			glnV	14.728		
			glnW	11.789		
			glnX	14.728		
GlyRS	169.941	434.046	glyT	33.604	14.7	11.6
			glyU	24.735		
			glyV	6.183		
			glyW	6.183		
			glyX	6.183		
			glyY	6.183		
HisRS	386.208	87.984	hisR	10.404		
IleRS	41.410	0.014	ileT	4.381	12.4	3.3
			ileU	4.381		
			ileV	4.381		
			ileX	63.612		
			ileY	63.612		
LeuRS	167.937	194.859	leuP	6.413	23.2	7.2
			leuQ	6.413		
			leuT	6.413		

Table S2 - continued.

tRNA Synthetase	Optimized Parameters k_{cat} (s^{-1})	$K_{M,A}$ (μM)	$K_{M,T}$ (μM)		Estimated [16] Lower Limit of k_{cat} (s^{-1})	Fold Change Above Estimated Lower Limit of k_{cat}
			leuU	7.345		
			leuV	6.413		
			leuW	22.547		
			leuX	25.764		
			leuZ	14.694		
LysRS	177.567	345.351	lsyQ	15.533	28.3	6.3
			lsyT	15.533		
			lysV	15.533		
			lysW	15.533		
			lysY	15.533		
			lysZ	15.533		
MetRS	72.906	42.364	metT	2.749		
			metU	2.749		
			metV	18.086		
			metW	18.086		
			metY	18.086		
			metZ	18.086		
PheRS	260.578	80.895	pheU	8.884	10.0	26.1
			pheV	8.884		
ProRS	76.461	79.973	proK	3.646		
			proL	13.006		
			proM	2.878		
SerRS	94.790	434.817	serT	26.791		
			serU	47.793		
			serV	9.048		
			serW	2.437		
			serX	2.437		
ThrRS	283.996	1706.487	thrT	2.564	48.0	5.9
			thrU	18.277		
			thrV	2.564		
			thrW	33.945		
TrpRS	66.962	0.080	trpT	8.749		
TyrRS	164.508	180.620	tyrT	13.629		
			tyrU	13.629		
			tyrV	13.629		
ValRS	97.125	264.722	valT	8.521	26.6	3.7
			valU	8.521		
			valV	3.159		
			valW	3.159		
			valX	8.521		
			valY	8.521		
			valZ	8.521		

Table S2 - continued.

Table S3. Aminoacyl-tRNA synthetase properties. Comparison of the measured and optimized k_{cat}s are shown, alongside the simulated molecular abundances of aminoacyl-tRNA synthetases. Other simulated properties: Ribosome elongation rate: 17.5 amino acids / ribosome / second; Active ribosomes per cell: 20,163 ± 4,209; Inactive ribosomes per cell: 3,220 ± 681. Note: [a]: mean ± standard deviation of measurements reported in Figure 3H.

Aminoacyl-tRNA Synthetase	Measured k_{cat} (s^{-1}) [a]	Optimized k_{cat} (s^{-1})	Simulated Total Aminoacyl-tRNA Synthetase per Cell [a]
AlaRS	28.6 ± 39.2	476.057	688.9 ± 188.4
ArgRS	19.0 ± 7.9	210.989	625.8 ± 195.5
AsnRS	1.4 ± 0.2	140.324	2140.5 ± 545.4
AspRS	14.8 ± 9.1	579.774	567.4 ± 162.9
CysRS	23.1 ± 35.9	69.447	606.4 ± 232.0
GluRS	2.6 ± 1.1	59.131	2864.0 ± 709.3
GlnRS	2.9 ± 1.4	208.545	1310.7 ± 364.5
GlyRS	25.7 ± 11.0	169.941	804.7 ± 203.6
HisRS	71.0 ± 60.5	386.208	427.6 ± 107.5
IleRS	54.6 ± 45.6	41.410	1698.2 ± 425.4
LeuRS	7.5 ± 7.4	167.937	1278.3 ± 353.5
LysRS	7.0 ± 9.7	177.567	1409.8 ± 360.2
MetRS	6.2 ± 0.6	72.906	843.1 ± 242.6
PheRS	36.4 ± 72.8	260.578	772.5 ± 184.7
ProRS	42.0 ± 28.0	76.461	862.8 ± 216.6
SerRS	1.1 ± 1.1	94.790	1438.3 ± 371.8
ThrRS	34.5 ± 33.9	283.996	1824.4 ± 467.6
TrpRS	2.6 ± 1.3	66.962	879.8 ± 286.6
TyrRS	12.0 ± 0.0	164.508	994.8 ± 259.4
ValRS	12.6 ± 8.9	97.125	1624.9 ± 465.5

Table S4. Properties of the 114 genes exhibiting tandemly arranged CGG codons in their codon sequences. The fold change in expression between simulations using the optimized and measured ArgRS k_{cat} are reported here and also depicted as a violin plot in Figure S5C. The gene abbreviation, EcoCyc ID, and gene name were taken from the EcoCyc database [3].

Expression Fold Change	Gene Abbreviation	EcoCyc ID	Gene Name
0.010	folA	DIHYDROFOLATEREDUCT-MONOMER	dihydrofolate reductase
0.012	lpxM	MYRISTOYLACYLTRAN-MONOMER	Lipid A biosynthesis myristoyltransferase
0.015	dgcP	G6980-MONOMER	diguanylate cyclase DgcP
0.015	rseP	EG12436-MONOMER	intramembrane zinc metalloprotease RseP
0.018	gntR	PD03585	DNA-binding transcriptional repressor GntR
0.019	ybjD	G6459-MONOMER	DUF2813 domain-containing protein YbjD
0.021	argA	N-ACETYLTRANSFER-MONOMER	N-acetylglutamate synthase
0.022	yeaH	G6970-MONOMER	DUF444 domain-containing protein YeaH
0.022	yegH	G7108-MONOMER	inner membrane protein YegH
0.023	yohD	EG12017-MONOMER	DedA family protein YohD
0.023	xthA	EG11073-MONOMER	exodeoxyribonuclease III
0.023	fieF	YIIP-MONOMER	Zn2+/Fe2+/Cd2+ exporter
0.023	rep	EG10837-MONOMER	ATP-dependent DNA helicase Rep
0.026	yaeQ	G6098-MONOMER	uncharacterized protein YaeQ
0.026	yfaY	G7162-MONOMER	IPR008135 CinA family protein YfaY
0.026	yfaU	G7158-MONOMER	2-keto-3-deoxy-L-rhamnonate aldolase
0.026	hypA	EG10483-MONOMER	hydrogenase 3 nickel incorporation protein HypA
0.027	lplA	EG11796-MONOMER	lipoate—protein ligase A
0.027	insEF5	MONOMER0-4249	IS3 element transposase
0.028	helD	EG10426-MONOMER	DNA helicase IV
0.029	ybbJ	G6264-MONOMER	NfeD-like family protein YbbJ
0.029	smrA	G6672-MONOMER	DNA endonuclease SmrA
0.030	lolE	YCFW-MONOMER	lipoprotein release complex - inner membrane subunit
0.031	hemY	EG10434-MONOMER	protein HemY
0.031	hrpA	G6732-MONOMER	ATP-dependent 3'→5' RNA helicase HrpA
0.032	glgX	EG10381-MONOMER	limit dextrin -1,6-glucohydrolase
0.033	lepB	EG10530-MONOMER	signal peptidase I
0.033	kup	KUP-MONOMER	K+:H+ symporter Kup
0.033	glnE	GLNE-MONOMER	fused glutamine synthetase deadenylase /glutamine synthetase adenylyltransferase
0.033	menF	MENF-MONOMER	isochorismate synthase MenF
0.034	cca	EG10136-MONOMER	fused CCA tRNA nucleotidyltransferase /phosphohydrolase
0.035	nudF	EG12633-MONOMER	ADP-sugar pyrophosphatase
0.037	yaiW	G6227-MONOMER	surface-exposed outer membrane lipoprotein
0.037	bluF	G6603-MONOMER	blue light- and temperature-regulated antirepressor BluF
0.039	cynR	PD00291	DNA-binding transcriptional dual regulator CynR
0.039	ampG	AMPG-MONOMER	muropeptide:H+ symporter
0.041	yjbB	EG11919-MONOMER	putative inorganic phosphate export protein YjbB
0.042	oppB	OPPB-MONOMER	murein tripeptide ABC transporter / oligopeptide ABC transporter inner membrane subunit OppB
0.042	clsB	G6406-MONOMER	cardiolipin synthase B
0.044	ymfK	G6589-MONOMER	e14 prophage; putative repressor protein YmfK
0.044	ygfX	G7509-MONOMER	protein YgfX
0.044	insEF2	MONOMER0-4246	IS3 element transposase
0.044	cynX	CYNX-MONOMER	cyanate transporter
0.045	insF2	MONOMER0-4441	IS3 element protein Ins
0.045	insEF1	MONOMER0-2748	IS3 element transposase
0.046	dnaE	EG10238-MONOMER	DNA polymerase III subunit
0.048	dcp	EG10212-MONOMER	peptidyl-dipeptidase Dcp
0.051	ydfV	G6832-MONOMER	Qin prophage; protein YdfV
0.052	malZ	MALTODEXGLUCOSID-MONOMER	maltodextrin glucosidase
0.054	insF4	MONOMER0-4443	IS3 element protein InsF
0.055	atoC	ATOC-MONOMER	DNA-binding transcriptional activator/ornithine decarboxylase inhibitor AtoC
0.055	pncC	G7409-MONOMER	NMN amidohydrolase
0.056	yafZ	G6127-MONOMER	CP4-6 prophage; DUF932 domain-containin protein YafZ
0.056	emrB	EMRB-MONOMER	multidrug efflux pump membrane subunit EmrB
0.058	cheR	CHER-MONOMER	chemotaxis protein methyltransferase
0.058	paaZ	G6708-MONOMER	oxepin-CoA hydrolase [multifunctional]
0.063	hyfI	MONOMER0-151	hydrogenase 4 catalytic subunit HyfI
0.066	ampE	EG10042-MONOMER	protein AmpE
0.066	nadB	L-ASPARTATE-OXID-MONOMER	L-aspartate oxidase
0.067	ppk	PPK-MONOMER	polyphosphate kinase
0.068	insF3	MONOMER0-4442	DLP12 prophage; IS3 element protein InsF

Table S4

Expression Fold Change	Gene Abbreviation	EcoCyc ID	Gene Name
0.068	flk	EG10229-MONOMER	putative flagella assembly protein
0.068	recG	EG10829-MONOMER	ATP-dependent DNA helicase RecG
0.069	yfiE	EG11785-MONOMER	putative LysR-type DNA-binding transcriptional regulator YfiE
0.070	aceK	ICITDEHASE-KIN-PHOSPHA	isocitrate dehydrogenase kinase / isocitrate dehydrogenase phosphatase
0.070	yfeZ	G7268-MONOMER	putative inner membrane protein
0.071	yphC	YPHC-MONOMER	putative zinc-binding dehydrogenase YphC
0.073	insEF4	MONOMER0-4248	IS3 element transposase
0.076	recF	EG10828-MONOMER	recombination mediator protein RecF
0.078	insF1	G7126-MONOMER	IS3 element protein InsF
0.079	yjiS	G7937-MONOMER	DUF1127 domain-containing protein YjiS
0.080	insF5	MONOMER0-4447	IS3 element protein InsF
0.080	umuC	EG11056-MONOMER	DNA polymerase V catalytic protein
0.082	yjiR	G7936-MONOMER	fused putative DNA-binding transcriptional regulator/putative aminotransferase YjiR
0.082	ylbF	G6289-MONOMER	DUF2877 domain-containing protein YlbF
0.084	hisQ	HISQ-MONOMER	lysine/arginine/ornithine ABC transporter / histidine ABC transporter, membrane subunit HisQ
0.087	zraR	HYDG-MONOMER	DNA-binding transcriptional activator ZraR
0.089	dgcC	EG11257-MONOMER	diguanylate cyclase DgcC
0.093	insEF3	MONOMER0-4247	IS3 element transposase
0.095	dinG	EG11357-MONOMER	ATP-dependent DNA helicase DinG
0.095	lysR	PD00360	DNA-binding transcriptional dual regulator LysR
0.097	yjfP	G7853-MONOMER	carboxylesterase
0.098	yhgN	G7753-MONOMER	MarC family putative inner membrane protein YhgN
0.098	thiK	THIKIN-MONOMER	thiamine kinase
0.102	tmcA	G7297-MONOMER	tRNAMet cytidine acetyltransferase
0.107	ynfF	G6846-MONOMER	putative selenate reductase YnfF
0.108	xynR	G6144-MONOMER	CP4-6 prophage; DNA-binding transcriptional repressor XynR
0.111	recD	EG10826-MONOMER	exodeoxyribonuclease V subunit RecD
0.117	ymfN	G6593-MONOMER	e14 prophage; chimeric replication protein/phage terminase YmfN
0.119	lhr	EG11548-MONOMER	ATP-dependent helicase Lhr
0.128	eutA	G7281-MONOMER	ethanolamine ammonia-lyase reactivase EutA
0.128	rimK	EG10852-MONOMER	ribosomal protein S6 modification protein
0.140	narU	NARU-MONOMER	nitrate/nitrite transporter NarU
0.156	yfhH	EG12308-MONOMER	putative DNA-binding transcriptional regulator YfhH
0.166	yidL	EG11707-MONOMER	putative DNA-binding transcriptional regulator YidL
0.172	prpR	G6195-MONOMER	DNA-binding transcriptional dual regulator PrpR
0.176	ydcJ	G6738-MONOMER	DUF1338 domain-containing protein YdcJ
0.180	mutM	EG10329-MONOMER	DNA-formamidopyrimidine glycosylase
0.190	murR	G7262-MONOMER	DNA-binding transcriptional dual regulator MurR
0.193	ybjO	G6448-MONOMER	putative inner membrane protein
0.197	hscC	G6357-MONOMER	chaperone protein HscC
0.204	rpnC	EG11748-MONOMER	recombination-promoting nuclease RpnC
0.260	yccM	G6513-MONOMER	putative electron transport protein YccM
0.301	priC	EG10765-MONOMER	primosomal replication protein N
0.311	yagF	G6141-MONOMER	CP4-6 prophage; D-xylonate dehydratase
0.318	bcsA	EG12260-MONOMER	cellulose synthase catalytic subunit
0.336	lsrC	YDEY-MONOMER	Autoinducer-2 ABC transporter membrane subunit LsrC
0.469	tauC	TAUC-MONOMER	taurine ABC transporter membrane subunit
0.565	rhsD	EG10849-MONOMER	protein RhsD
0.578	yciQ	G6636-MONOMER	DUF2207 domain-containing protein YciQ
0.700	hcr	G6456-MONOMER	NADH oxidoreductase
0.778	ybdN	G6331-MONOMER	putative PAPS reductase/DUF3440 domain-containing protein YbdN
1.000	mgtT	MONOMER0-4487	protein MgtT
1.300	ubiV	G7653-MONOMER	ubiquinone biosynthesis protein UbiV

References

[1] Macklin, D., Ahn-Horst, T., Choi, H., Ruggero, N., Carrera, J., Mason, J., Sun, G., Agmon, E., DeFelice, M., Maayan, I., Lane, K., Spangler, R., Gillies, T., Paull, M., Akhter, S., Bray, S., Weaver, D., Keseler, I., Karp, P., Morrison, J., and Covert, M. (2020) Simultaneous cross-evaluation of heterogeneous E. coli datasets via mechanistic simulation. *Science,* **369**(6502), eaav3751.

[2] Liebermeister, W. and Klipp, E. (2006) Bringing metabolic networks to life: Convenience rate law and thermodynamic constraints. *Theoretical Biology and Medical Modelling,* **3**(41).

[3] Keseler, I. M., Mackie, A., Peralta-Gil, M., Santos-Zavaleta, A., Gama-Castro, S., Bonavides-Martínez, C., Fulcher, C., Huerta, A. M., Kothari, A., Krummenacker, M., et al. (2012) EcoCyc: fusing model organism databases with systems biology. *Nucleic acids research,* **41**(D1), D605–D612.

[4] Crick, F. (1966) Codon—anticodon pairing: The wobble hypothesis. *Journal of Molecular Biology,* **19**(2), 548–555.

[5] Komine, Y., Adachi, T., Inokuchi, H., and Ozeki, H. (1990) Genomic organization and physical mapping of the transfer RNA genes in Escherichia coli K12. *Journal of Molecular Biology,* **212**(4), 579–98.

[6] Agris, P., Narendran, A., Sarachan, K., Väre, V., and Eruysal, E. (2017) The Importance of Being Modified: The Role of RNA Modifications in Translational Fidelity. *The Enzymes,* **41**, 1–50.

[7] Muramatsu, T., Nishikawa, K., Nemoto, F., Kuchino, Y., Nishimura, S., Miyazawa, T., and S, Y. (1988) Codon and amino-acid specificities of a transfer RNA are both converted by a single post-transcriptional modification. *Nature,* **336**, 179–181.

[8] Nilsson, E. and Alexander, R. (2019) Bacterial wobble modifications of NNA-decoding tRNAs. *IUBMB Life,* **71**(8), 1158–1166.

[9] Meinnel, T., Mechulam, Y., and Blanquet, S. (1993) Methionine as translation start signal: a review of the enzymes of the pathway in Escherichia coli.. *Biochimie,* **75**(12), 1061–75.

[10] Brunschede, H., Dove, T., and Bremer, H. (1977) Establishment of exponential growth after a nutritional shift-up in Escherichia coli B/r: accumulation of deoxyribonucleic acid, ribonucleic acid and protein. *Journal of Bacteriology,* **129**, 1020–1033.

[11] Reshes, G., Vanounou, S., Fishov, I., and Feingold, M. (2008) Timing the start of division in E. coli: a single-cell study. *Physical Biology,* **5**.

[12] Schmidt, A., Kochanowski, K., Vedelaar, S., Ahrné, E., Volkmer, B., Callipo, L., Knoops, K., Bauer, M., Aebersold, R., and Heinemann, M. (2016) The quantitative and condition-dependent Escherichia coli proteome. *Nature biotechnology,* **34**(1), 104–110.

[13] Taniguchi, Y., Choi, P. J., Li, G.-W., Chen, H., Babu, M., Hearn, J., Emili, A., and Xie, X. S. (2010) Quantifying E. coli proteome and transcriptome with single-molecule sensitivity in single cells. *Science,* **329**(5991), 533–538.

[14] Dong, H., Nilsson, L., and Kurland, C. (1996) Co-variation of tRNA abundance and codon usage in Escherichia coli at different growth rates. *Journal of Molecular Biology,* **260**(5), 649–63.

[15] Bremer, H. and Dennis, P. P. (2008) Modulation of chemical composition and other parameters of the cell at different exponential growth rates. *EcoSal Plus,* pp. 1–49.

[16] Jakubowski, H. and Goldman, E. (1984) Quantities of Individual Aminoacyl-tRNA Families and Their Turnover in Escherichia coli.. *Journal of Bacteriology,* **158**(3), 769–776.

[17] Mohammad, F., Green, R., and Buskirk, A. (2019) A systematically-revised ribosome profiling method for bacteria reveals pauses at single-codon resolution. *eLIFE,* **8**(e42591), 1–25.

[18] Sørensen, M. (2001) Charging levels of four tRNA species in Escherichia coli Rel(+) and Rel(-) strains during amino acid starvation: a simple model for the effect of ppGpp on translational accuracy. *J Mol Biol,* **307**(3), 785–98.

[19] Krüger, M. and Sørensen, M. (1998) Aminoacylation of hypomodified tRNAGlu in vivo. *J Mol Biol,* **284**(3), 609–20.

[20] Avcilar-Kucukgoze, I., Bartholomäus, A., Cordero Varela, J., Kaml, R., Neubauer, P., Budisa, N., and Ignatova, Z. (2016) Discharging tRNAs: a tug of war between translation and detoxification in Escherichia coli. *Nucleic Acids Res,* **44**(17), 8324–34.

[21] Yegian, C., Stent, G., and Martin, E. (1966) Intracellular condition of Escherichia coli transfer RNA. *Proc Natl Acad Sci U S A,* **55**(4), 839–46.

[22] Wu, M., Filley, S., Xiong, J., Lee, J., and Hill, K. (1994) A cysteine in the C-terminal region of alanyl-tRNA synthetase is important for aminoacylation activity. *Biochemistry,* **33**(40), 12260–6.

[23] Filley, S. and Hill, K. (1993) Amino acid substitutions at position 73 in motif 2 of Escherichia coli alanyl-tRNA synthetase. *Arch Biochem Biophys,* **307**(1), 46–51.

[24] Barends, S., Wower, J., and Kraal, B. (2000) Kinetic parameters for tmRNA binding to alanyl-tRNA synthetase and elongation factor Tu from Escherichia coli. *Biochemistry,* **39**(10), 2652–8.

[25] Pleiss, J., Wolfson, A., and Uhlenbeck, O. (2000) Mapping contacts between Escherichia coli alanyl tRNA synthetase and 2' hydroxyls using a complete tRNA molecule. *Biochemistry,* **39**(28), 8250–8.

[26] Zhang, C., Perona, J., Ryu, K., Francklyn, C., and Hou, Y. (2006) Distinct kinetic mechanisms of the two classes of Aminoacyl-tRNA synthetases. *Journal of Molecular Biology,* **361**(2), 300–11.

[27] Guo, M., Chong, Y., Shapiro, R., Beebe, K., Yang, X., and Schimmel, P. (2009) Paradox of mistranslation of serine for alanine caused by AlaRS recognition dilemma. *Nature,* **462**(7274), 808–12.

[28] Loftfield, R. (1972) The mechanism of aminoacylation of transfer RNA. *Progress in Nucleic Acid Research and Molecular Biology,* **12**, 87–128.

[29] Zhang, Q., Wang, E., and Wang, Y. (1998) The role of tryptophan residues in Escherichia coli arginyl-tRNA synthetase. *Biochimica et Biophysica Acta,* **1387**(1-2), 136–42.

[30] Kiga, D., Sakamoto, K., Sato, S., Hirao, I., and Yokoyama, S. (2001) Shifted positioning of the anticodon nucleotide residues of amber suppressor tRNA species by Escherichia coli arginyl-tRNA synthetase. *European Journal of Biochemistry,* **268**(23), 6207–13.

[31] Yao, Y., Zhang, Q., Yan, X., Zhu, G., and Wang, E. (2004) Escherichia coli tRNA(4)(Arg)(UCU) induces a constrained conformation of the crucial Omega-loop of arginyl-tRNA synthetase. *Biochemical and Biophysical Research Communications,* **313**(1), 129–34.

[32] Madern, D., Anselme, J., and Härtlein, M. (1992) Asparaginyl-tRNA synthetase from the Escherichia coli temperature-sensitive strain HO202. A proline replacement in motif 2 is responsible for a large increase in Km for asparagine and ATP.. *FEBS Letters,* **299**(1), 85–9.

[33] Anselme, J. and Härtlein, M. (1991) Tyr-426 of the Escherichia coli asparaginyl-tRNA synthetase, an amino acid in a C-terminal conserved motif, is involved in ATP binding. *FEBS Letters,* **280**(1), 163–6.

[34] Martin, F., Sharples, G., Lloyd, R., Eiler, S., Moras, D., Gangloff, J., and Eriani, G. (1997) Characterization of a thermosensitive Escherichia coli aspartyl-tRNA synthetase mutant. *Journal of Bacteriology,* **179**(11), 3691–6.

[35] Sekine, S., Nureki, O., Tateno, M., and Yokoyama, S. (1999) The identity determinants required for the discrimination between tRNAGlu and tRNAAsp by glutamyl-tRNA synthetase from Escherichia coli. *European Journal of Biochemistry,* **261**(2), 354–60.

[36] Brevet, A., Chen, J., Commans, S., Lazennec, C., Blanquet, S., and Plateau, P. (2003) Anticodon recognition in evolution: switching tRNA specificity of an aminoacyl-tRNA synthetase by site-directed peptide transplantation. *Journal of Biological Chemistry,* **278**(33), 30927–35.

[37] Dubois, D., Blaise, M., Becker, H., Campanacci, V., Keith, G., Giegé, R., Cambillau, C., Lapointe, J., and Kern, D. (2004) An aminoacyl-tRNA synthetase-like protein encoded by the Escherichia coli yadB gene glutamylates specifically tRNAAsp. *Proceedings of the National Academy of Sciences U S A,* **101**(20), 7530–5.

[38] Martin, F., Barends, S., and Eriani, G. (2004) Single amino acid changes in AspRS reveal alternative routes for expanding its tRNA repertoire in vivo. *Nucleic Acids Research,* **32**(13), 4081–9.

[39] Sherlin, L., Bullock, T., Newberry, K., Lipman, R., Hou, Y., Beijer, B., Sproat, B., and Perona, J. (2000) Influence of transfer RNA tertiary structure on aminoacylation efficiency by glutaminyl and cysteinyl-tRNA synthetases. *J Mol Biol,* **299**(2), 431–46.

[40] Zhang, C., Christian, T., Newberry, K., Perona, J., and Hou, Y. (2003) Zinc-mediated amino acid discrimination in cysteinyl-tRNA synthetase. *J Mol Biol,* **327**(5), 911–7.

[41] Ruan, B., Nakano, H., Tanaka, M., Mills, J., DeVito, J., Min, B., Low, K., Battista, J., and Söll, D. (2004) Cysteinyl-tRNA(Cys) formation in Methanocaldococcus jannaschii: the mechanism is still unknown. *J Bacteriol,* **186**(1), 8–14.

[42] Hauenstein, S., Zhang, C., Hou, Y., and Perona, J. (2004) Shape-selective RNA recognition by cysteinyl-tRNA synthetase. *Nat Struct Mol Biol,* **11**(11), 1134–41.

[43] Zhang, C. and Hou, Y. (2005) Domain-domain communication for tRNA aminoacylation: the importance of covalent connectivity. *Biochemistry,* **44**(19), 7240–9.

[44] Kern, D., Potier, S., Boulanger, Y., and Lapointe, J. (1979) The monomeric glutamyl-tRNA synthetase of Escherichia coli. Purification and relation between its structural and catalytic properties. *J Biol Chem,* **254**(2), 518–24.

[45] Lapointe, J., Levasseur, S., and Kern, D. (1985) Glutamyl-tRNA synthetase from Escherichia coli. *Methods Enzymol,* **113**, 42–9.

[46] Lapointe, J. and Söll, D. (1972) Glutamyl transfer ribonucleic acid synthetase of Escherichia coli. 3. Influence of the 46K protein on the affinity of the 56K glutamyl transfer ribonucleic acid synthetase for its substrates. *J Biol Chem,* **247**(16), 4982–5.

[47] Hoben, P., Royal, N., Cheung, A., Yamao, F., Biemann, K., and Söll, D. (1982) Escherichia coli glutaminyl-tRNA synthetase. II. Characterization of the glnS gene product. *J Biol Chem,* **257**(19), 11644–50.

[48] Kern, D., Potier, S., Lapointe, J., and Boulanger, Y. (1980) The glutaminyl-transfer RNA synthetase of Escherichia coli. Purification, structure and function relationship. *Biochim Biophys Acta,* **607**(1), 65–80.

[49] Sherman, J., Thomann, H., and Söll, D. (1996) Functional connectivity between tRNA binding domains in glutaminyl-tRNA synthetase. *J Mol Biol,* **256**(5), 818–28.

[50] Ibba, M., Hong, K., Sherman, J., Sever, S., and Söll, D. (1996) Interactions between tRNA identity nucleotides and their recognition sites in glutaminyl-tRNA synthetase determine the cognate amino acid affinity of the enzyme. *Proc Natl Acad Sci U S A,* **93**(14), 6953–8.

[51] Liu, J., Ibba, M., Hong, K., and Söll, D. (1998) The terminal adenosine of tRNA(Gln) mediates tRNA-dependent amino acid recognition by glutaminyl-tRNA synthetase. *Biochemistry,* **37**(27), 9836–42.

[52] Bullock, T., Rodríguez-Hernández, A., Corigliano, E., and Perona, J. (2008) A rationally engineered misacylating aminoacyl-tRNA synthetase. *Proc Natl Acad Sci U S A,* **105**(21), 7428–33.

[53] Ostrem, D. and P, B. (1974) Glycyl transfer ribonucleic acid synthetase from Escherichia coli: purification, properties, and substrate binding. *Biochemistry,* **13**(7), 1338–48.

[54] Augustine, J. and Francklyn, C. (1997) Design of an active fragment of a class II aminoacyl-tRNA synthetase and its significance for synthetase evolution. *Biochemistry,* **36**(12), 3473–82.

[55] Rühlmann, A., Cramer, F., and Englisch, U. (1997) Isolation and analysis of mutated histidyl-tRNA synthetases from Escherichia coli. *Biochem Biophys Res Commun,* **237**(1), 192–201.

[56] Connolly, S., Rosen, A., Musier-Forsyth, K., and Francklyn, C. (2004) G-1:C73 recognition by an arginine cluster in the active site of Escherichia coli histidyl-tRNA synthetase. *Biochemistry,* **43**(4), 962–9.

[57] Guth, E., Connolly, S., Bovee, M., and Francklyn, C. (2005) A substrate-assisted concerted mechanism for aminoacylation by a class II aminoacyl-tRNA synthetase. *Biochemistry,* **44**(10), 3785–94.

[58] Rosen, A., Brooks, B., Guth, E., Francklyn, C., and Musier-Forsyth, K. (2006) Evolutionary conservation of a functionally important backbone phosphate group critical for aminoacylation of histidine tRNAs. *RNA,* **12**(7), 1315–22.

[59] Fersht, A. and Kaethner, M. (1976) Mechanism of aminoacylation of tRNA. Proof of the aminoacyl adenylate pathway for the isoleucyl- and tyrosyl-tRNA synthetases from Escherichia coli K12. *Biochemistry,* **15**(4), 818–23.

[60] L, Z. and Rosevear, P. (1995) Mutation of the carboxy terminal zinc finger of E. coli isoleucyl-tRNA synthetase alters zinc binding and aminoacylation activity. *Biochem Biophys Res Commun,* **216**(2), 648–54.

[61] Landro, J., Schmidt, E., Schimmel, P., Tierney, D., and Penner-Hahn, J. (1994) Thiol ligation of two zinc atoms to a class I tRNA synthetase: evidence for unshared thiols and role in amino acid binding and utilization. *Biochemistry,* **33**(47), 14213–20.

[62] Xu, B., Trawick, B., Krudy, G., Phillips, R., Zhou, L., and Rosevear, P. (1994) Probing the metal binding sites of Escherichia coli isoleucyl-tRNA synthetase. *Biochemistry,* **33**(2), 398–402.

[63] Glasfeld, E., Landro, J., and Schimmel, P. (1996) C-terminal zinc-containing peptide required for RNA recognition by a class I tRNA synthetase. *Biochemistry,* **35**(13), 4139–45.

[64] Chen, J., Li, Y., Wang, E., and Wang, Y. (1999) High-level expression and single-step purification of leucyl-tRNA synthetase from Escherichia coli. *Protein Expr Purif,* **15**(1), 115–20.

[65] Chen, J., Guo, N., Li, T., Wang, E., and Wang, Y. (2000) CP1 domain in Escherichia coli leucyl-tRNA synthetase is crucial for its editing function. *Biochemistry,* **39**(22), 6726–31.

[66] Chen, J., Li, T., Wang, E., and Wang, Y. (2001) Effect of alanine-293 replacement on the activity, ATP binding, and editing of Escherichia coli leucyl-tRNA synthetase. *Biochemistry,* **40**(5), 1144–9.

[67] Du, X. and Wang, E. (2003) E292 is important for the aminoacylation activity of Escherichia coli leucyl-tRNA synthetase. *J Protein Chem,* **22**(1), 71–6.

[68] Mursinna, R., Lee, K., Briggs, J., and Martinis, S. (2004) Molecular dissection of a critical specificity determinant within the amino acid editing domain of leucyl-tRNA synthetase. *Biochemistry,* **43**(1), 155–65.

[69] Xu, M., Li, J., Du, X., and Wang, E. (2004) Groups on the side chain of T252 in Escherichia coli leucyl-tRNA synthetase are important for discrimination of amino acids and cell viability. *Biochem Biophys Res Commun,* **318**(1), 11–6.

[70] Lue, S. and Kelley, S. (2005) An aminoacyl-tRNA synthetase with a defunct editing site. *Biochemistry,* **44**(8), 3010–6.

[71] Zhai, Y., Nawaz, M., Lee, K., Kirkbride, E., Briggs, J., and Martinis, S. (2007) Modulation of substrate specificity within the amino acid editing site of leucyl-tRNA synthetase. *Biochemistry,* **46**(11), 3331–7.

[72] Lue, S. and Kelley, S. (2007) A single residue in leucyl-tRNA synthetase affecting amino acid specificity and tRNA aminoacylation. *Biochemistry,* **46**(15), 4466–72.

[73] Vu, M. and Martinis, S. (2007) A unique insert of leucyl-tRNA synthetase is required for aminoacylation and not amino acid editing. *Biochemistry,* **46**(17), 5170–6.

[74] Ibba, M., Losey, H., Kawarabayasi, Y., Kikuchi, H., Bunjun, S., and Söll, D. (1999) Substrate recognition by class I lysyl-tRNA synthetases: a molecular basis for gene displacement. *Proc Natl Acad Sci U S A,* **96**(2), 418–23.

[75] Jakubowski, H. (1999) Misacylation of tRNALys with noncognate amino acids by lysyl-tRNA synthetase. *Biochemistry,* **38**(25), 8088–93.

[76] of cognate, D. and noncognate substrates at the active site of class II lysyl-tRNA synthetase (2004) Discrimination of cognate and noncognate substrates at the active site of class II lysyl-tRNA synthetase. *Biochemistry,* **43**(37), 11836–41.

[77] Meinnel, T., Mechulam, Y., Dardel, F., Schmitter, J., Hountondji, C., Brunie, S., Dessen, P., Fayat, G., and Blanquet, S. (1990) Methionyl-tRNA synthetase from E. coli–a review. *Biochimie,* **72**(8), 625–32.

[78] Kim, S., Jo, Y., Lee, S., Motegi, H., Shiba, K., Sassanfar, M., and Martinis, S. (1998) Biochemical and phylogenetic analyses of methionyl-tRNA synthetase isolated from a pathogenic microorganism, Mycobacterium tuberculosis. *FEBS Lett,* **427**(2), 259–62.

[79] Crepin, T., Schmitt, E., Blanquet, S., and Mechulam, Y. (2002) Structure and function of the C-terminal domain of methionyl-tRNA synthetase. *Biochemistry,* **41**(43), 13003–11.

[80] Casina, V., Lobashevsky, A., McKinney, W., Brown, C., and Alexander, R. (2011) Role for a conserved structural motif in assembly of a class I aminoacyl-tRNA synthetase active site. *Biochemistry,* **50**(5), 763–9.

[81] Jo, Y., Lee, S., Jo, M., Lee, J., Kang, M., Yoon, J., and Kim, S. (1999) Methionine analogue probes functionally important residues in active site of methionyl-tRNA synthetase. *J. Biochem. Mol. Biol.,* **32**, 547–553.

[82] Kiick, K. and Tirrell, D. (2000) Protein Engineering by In Vivo Incorporation of Non-Natural Amino Acids: Control of Incorporation of Methionine Analogues by Methionyl-tRNA Synthetase. *Tetrahedron,* **56**, 9487–9493.

[83] Ibba, M., Kast, P., and Hennecke, H. (1994) Substrate specificity is determined by amino acid binding pocket size in Escherichia coli phenylalanyl-tRNA synthetase. *Biochemistry,* **33**(23), 7107–12.

[84] Bentin, T., Hamzavi, R., Salomonsson, J., Roy, H., Ibba, M., and Nielsen, P. (2004) Photoreactive bicyclic amino acids as substrates for mutant Escherichia coli phenylalanyl-tRNA synthetases. *J Biol Chem,* **279**(19), 19839–45.

[85] Roy, H., Ling, J., Irnov, M., and Ibba, M. (2004) Post-transfer editing in vitro and in vivo by the beta subunit of phenylalanyl-tRNA synthetase. *EMBO J,* **23**(23), 4639–48.

[86] Roy, H. and Ibba, M. (2006) Phenylalanyl-tRNA synthetase contains a dispensable RNA-binding domain that contributes to the editing of noncognate aminoacyl-tRNA. *Biochemistry,* **45**(30), 9156–62.

[87] Reynolds, N., Ling, J., Roy, H., Banerjee, R., Repasky, S., Hamel, P., and Ibba, M. (2010) Cell-specific differences in the requirements for translation quality control. *Proc Natl Acad Sci U S A,* **107**(9), 4063–8.

[88] Beuning, P. and Musier-Forsyth, K. (2001) Species-specific differences in amino acid editing by class II prolyl-tRNA synthetase. *J Biol Chem,* **276**(33), 30779–85.

[89] Ahel, I., Stathopoulos, C., Ambrogelly, A., Sauerwald, A., Toogood, H., Hartsch, T., and Söll, D. (2002) Cysteine activation is an inherent in vitro property of prolyl-tRNA synthetases. *J Biol Chem,* **277**(38), 34743–8.

[90] Hati, S., Ziervogel, B., Sternjohn, J., Wong, F., Nagan, M., Rosen, A., Siliciano, P., Chihade, J., and Musier-Forsyth, K. (2006) Pre-transfer editing by class II prolyl-tRNA synthetase: role of aminoacylation active site in "selective release" of noncognate amino acids. *J Biol Chem,* **281**(38), 27862–72.

[91] Borel, F., Vincent, C., Leberman, R., and Härtlein, M. (1994) Seryl-tRNA synthetase from Escherichia coli: implication of its N-terminal domain in aminoacylation activity and specificity. *Nucleic Acids Res,* **22**(15), 2963–9.

[92] Zheltonosova, J., Melnikova, E., Garber, M., Reinbolt, J., Kern, D., Ehresmann, C., and Ehresmann, B. (1994) Threonyl-tRNA synthetase from Thermus thermophilus: purification and some structural and kinetic properties. *Biochimie,* **76**(1), 71–7.

[93] Sankaranarayanan, R., Dock-Bregeon, A., Rees, B., Bovee, M., Caillet, J., Romby, P., Francklyn, C., and Moras, D. (2000) Zinc ion mediated amino acid discrimination by threonyl-tRNA synthetase. *Nat Struct Biol,* **7**(6), 461–5.

[94] Bovee, M., Pierce, M., and Francklyn, C. (2003) Induced fit and kinetic mechanism of adenylation catalyzed by Escherichia coli threonyl-tRNA synthetase. *Biochemistry,* **42**(51), 15102–13.

[95] Ruan, B., Bovee, M., Sacher, M., Stathopoulos, C., Poralla, K., Francklyn, C., and Söll, D. (2005) A unique hydrophobic cluster near the active site contributes to differences in borrelidin inhibition among threonyl-tRNA synthetases. *J Biol Chem,* **280**(1), 571–7.

[96] Ling, J. and Söll, D. (2010) Severe oxidative stress induces protein mistranslation through impairment of an aminoacyl-tRNA synthetase editing site. *Proc Natl Acad Sci U S A,* **107**(9), 4028–33.

[97] Sever, S., Rogers, K., Rogers, M., Carter, C. J., and Söll, D. (1996) Escherichia coli tryptophanyl-tRNA synthetase mutants selected for tryptophan auxotrophy implicate the dimer interface in optimizing amino acid binding. *Biochemistry,* **35**(1), 32–40.

[98] Zúñiga, R., Salazar, J., Canales, M., and Orellana, O. (2002) A dispensable peptide from Acidithiobacillus ferrooxidans tryptophanyl-tRNA synthetase affects tRNA binding. *FEBS Lett,* **532**(3), 387–90.

[99] Hamano-Takaku, F., Iwama, T., Saito-Yano, S., Takaku, K., Monden, Y., Kitabatake, M., Soll, D., and Nishimura, S. (2000) A mutant Escherichia coli tyrosyl-tRNA synthetase utilizes the unnatural amino acid azatyrosine more efficiently than tyrosine. *J Biol Chem,* **275**(51), 40324–8.

[100] Kiga, D., Sakamoto, K., Kodama, K., Kigawa, T., Matsuda, T., Yabuki, T., Shirouzu, M., Harada, Y., Nakayama, H., Takio, K., Hasegawa, Y., Endo, Y., Hirao, I., and Yokoyama, S. (2002) An engineered Escherichia coli tyrosyl-tRNA synthetase for site-specific incorporation of an unnatural amino acid into proteins in eukaryotic translation and its application in a wheat germ cell-free system. *Proc Natl Acad Sci U S A,* **99**(15), 9715–20.

[101] Hountondji, C., Lazennec, C., Beauvallet, C., Dessen, P., Pernollet, J., Plateau, P., and Blanquet, S. (2002) Crucial role of conserved lysine 277 in the fidelity of tRNA aminoacylation by Escherichia coli valyl-tRNA synthetase. *Biochemistry,* **41**(50), 14856–65.

[102] Tardif, K. and Horowitz, J. (2004) Functional group recognition at the aminoacylation and editing sites of E. coli valyl-tRNA synthetase. *RNA,* **10**(3), 493–503.
